# Additive Manufacturing of Biomaterials: Integrated Translational Ecosystems, Process–Structure–Property Interactions and Emerging Paradigms for Next-Generation Biomedical Engineering

**DOI:** 10.3390/jfb17080365

**Published:** 2026-07-30

**Authors:** André F. V. Pedroso, Luciana Silva, Marta L. S. Barbosa, Wenfeng Ding, Biao Zhao, Ning Qian, Francisco J. G. Silva

**Affiliations:** 1 CIDEM, ISEP, Polytechnic of Porto, Rua Dr. António Bernardino de Almeida, 4249-015 Porto, Portugal; 1190814@isep.ipp.pt (L.S.); martabarbosa8c@gmail.com (M.L.S.B.); fgs@isep.ipp.pt (F.J.G.S.); 2 Department of Mechanical Engineering, Faculty of Engineering, University of Porto, Rua Dr. Roberto Frias, 400, 4200-465 Porto, Portugal; 3 College of Mechanical and Electrical Engineering, Nanjing University of Aeronautics and Astronautics, Nanjing 210016, China; wfding@nuaa.edu.cn (W.D.); zhaobiao@nuaa.edu.cn (B.Z.); n.qian@nuaa.edu.cn (N.Q.); 4 Associate Laboratory for Energy, Transports and Aerospace, LAETA-INEGI, Campus FEUP, Rua Dr. Roberto Frias, 400, 4200-465 Porto, Portugal

**Keywords:** additive manufacturing, biomaterials, bioprinting, tissue engineering, process–structure–property relationships

## Abstract

Additive manufacturing (AM) has expanded the design space of biomaterials for biomedical engineering, enabling patient-specific geometries, controlled porosity, multi-material constructs and cell-compatible fabrication. However, clinical translation remains constrained by a mismatch between fabrication capability and biological, mechanical and regulatory performance. Unlike reviews focused on individual material families, isolated AM routes or specific applications, this review interprets AM of biomaterials as an integrated biomaterial–process–structure–property–translation ecosystem. It examines how material chemistry, feedstock state, printing route, architecture, post-processing and biological response jointly determine the reliability of acellular and cellular constructs, with particular emphasis on clinically relevant performance, reproducibility and long-term safety. Metallic, ceramic, polymeric, hydrogel-based and composite biomaterials are analysed alongside binder jetting (BJ), directed energy deposition (DED), material extrusion (ME) and jetting (MJ), powder bed fusion (PBF), VAT photopolymerisation and bioprinting. The review identifies recurring challenges across routes, including restricted material–process compatibility, limited prediction of process–structure–property relationships, post-processing-induced changes in biological performance, insufficient standardisation of printability and biofunctionality metrics, incomplete validation of cell-laden and vascularised constructs and weak transfer of laboratory protocols to clinically robust workflows. The main conclusion is that progress will depend less on expanding printable geometries alone and more on integrated optimisation of materials, processing windows, structural fidelity, biological validation, quality assurance and translational readiness. This ecosystem-level perspective provides a framework for evaluating limitations and defining future priorities in AM-based biomaterials for regenerative medicine, implants and precision biomedical engineering.

## 1. Introduction

Biomaterials are natural or synthetic substances that encounter biological systems and aid in repairing, replacing or enhancing any tissue or organ of the body over time [1]. Biomaterials possess immense potential for diverse applications across various fields. They demonstrate exceptional environmental stability, high stretchability and significant mechanical strength, making them highly suitable for applications in tissue regeneration, dental implants, biosensors and more. However, there is one fundamental property. Biocompatibility refers to a material’s ability to function in a specific application while inducing the desired response from the host. Biocompatibility is therefore application-specific and depends on material chemistry, surface condition, degradation behaviour, implantation site and host response. Under suitable conditions, biocompatible materials can interact with host tissues without inducing unacceptable immunological, allergic, inflammatory, carcinogenic or chronic adverse reactions [2,3].

The versatile properties of biomaterials contribute to their increasing relevance in both medical and non-medical applications. Examples include the incorporation of biocompatible ionic liquids in bioelectronic applications and the exploration of potential biomaterial combinations in regenerative medicine. In-depth studies are conducted to understand and retain the inherent properties of biomaterials, emphasising their characteristics for optimal utilisation.

Additive manufacturing (AM), commonly known as 3D printing, stands out as one of the most practical advanced manufacturing solutions for producing intricate structures. This technology finds widespread application in technology-driven industries, particularly in healthcare [4]. AM stands apart from other manufacturing methods, such as subtractive and formative techniques, by minimising material waste and enabling the creation of lightweight, intricate structures—often hollow or porous. This approach reduces the required material and energy inputs during both the fabrication process and the product’s use. Seven distinct categories of AM have been identified, including binder jetting (BJ) [5], directed energy deposition (DED), material extrusion (ME), material jetting (MJ), powder bed fusion (PBF), sheet lamination and VAT photopolymerisation (VPP). Emerging evidence indicates that the long-term sustainability of bioprinting depends not only on construct performance, but also on polymer circularity, recyclability and end-of-life management [6,7,8]. Fused deposition modelling (FDM), more formally classified as ME, fabricates components through the layer-wise deposition of thermoplastic or composite filaments. In biomaterial applications, its principal advantages include process simplicity, cost-effectiveness and architectural control; however, limited resolution, interlayer anisotropy and restricted biofabrication compatibility continue to constrain broader clinical deployment today. Sebbe et al. [9] addressed the challenge of overcoming the geometric, surface-quality and strength limitations of conventional planar FDM, while extending non-planar deposition to steeper unsupported angles. Optimised non-planar FDM improved tensile performance, markedly reduced surface roughness, enabled support-free suspended geometries and expanded printable inclinations up to 90°. Furthermore, Sebbe et al.’s [10] study addressed the challenge of converting a conventional three-axis FDM printer into a non-planar system capable of reducing stair-stepping, support dependence and geometric restriction. It was demonstrated that targeted mechanical redesign enabled non-planar deposition up to about 30°, increasing printable angle capability and extending the accessible non-planar build height.

In the context of this review, terminology is used according to the level of biological integration involved. AM is employed as the umbrella term for layer-wise fabrication technologies applied to biomaterials, including acellular and cellular approaches. 3D printing is used mainly as the widely adopted general synonym for AM, particularly when referring to the broader fabrication principle or when retained from the terminology used in the cited literature. Biofabrication denotes the broader production of biologically functional constructs through the controlled organisation of biomaterials, cells, bioactive molecules and supporting architectures. Bioprinting is used more specifically for AM-based biofabrication processes in which cell-laden or biologically active inks are deposited with spatial control to generate tissue-like constructs. Accordingly, the manuscript uses AM for the overall manufacturing field, biofabrication for the broader biological manufacturing context and bioprinting for cell-containing or bioink-based printing strategies.

To avoid ambiguity and ensure consistent use of terminology throughout the review, Table 1 defines the principal terms used to describe the manufacturing routes considered in this manuscript. The distinction is particularly important because *AM*, *3D printing*, *biofabrication* and *bioprinting* are related but not fully interchangeable concepts. The table therefore clarifies how each term is used in the present review, distinguishing between the general layer-wise fabrication of biomaterials, broader biologically oriented manufacturing strategies and cell-containing or bioink-based printing approaches.

This review begins by presenting how biomaterials can be classified based on the material that predominantly constitutes them. Next, the main AM methods are introduced. Finally, a multidisciplinary research approach will be essential to address these challenges and fully unlock the potential of AM in the future. Biomaterials are classified into four main categories based on their material type.

- Metals and Alloys:

Metallic biomaterials are widely used in load-bearing biomedical devices, including orthopaedic, dental, craniofacial and fixation applications, because they offer high strength, fracture resistance, fatigue performance and long-term mechanical stability [11,12]. Stainless steels, titanium and titanium alloys, cobalt–chromium alloys and, in specific contexts, biodegradable metallic systems based on magnesium, zinc or iron are among the most commonly investigated classes [13]. Their in vivo performance, however, cannot be generalised solely from their mechanical properties. Corrosion and degradation behaviour are strongly alloy-dependent and are governed by composition, passive-film stability, surface condition, microstructure, porosity, residual stress, manufacturing route and physiological environment [14]. Baakili et al. [15] examined the persistent challenge of mitigating biocorrosion in additively manufactured (AMed) porous metallic implants, while preserving mechanical reliability, interfacial stability and long-term biological performance. The authors critically reviewed linking AM, porous architecture and surface engineering to improved corrosion resistance, osseointegration potential and orthopaedic implant durability. Ti and CoCr-alloys, for example, are generally valued for their passive oxide films and corrosion resistance, whereas Mg-, Zn- and Fe-based systems are often investigated precisely because controlled degradation may be desirable in temporary implants [13]. In additively manufactured metallic biomaterials, surface roughness, unmelted particles, residual porosity, heat-treatment condition and post-processing route may further influence ion release, fatigue resistance, osseointegration and inflammatory response [15,16]. Therefore, corrosion should be interpreted as a material-, process- and environment-dependent phenomenon rather than as a universal behaviour of metallic biomaterials.

- Ceramics:

Bioceramics comprise a broad group of inorganic non-metallic biomaterials, including bioinert ceramics, such as alumina and zirconia, bioactive ceramics, such as bioactive glasses and hydroxyapatite and resorbable calcium-phosphate-based systems [17,18]. Their biomedical relevance is closely linked to high compressive strength, wear resistance, chemical stability or bioactivity, depending on composition and processing route [18]. However, their behaviour should not be described as uniformly inert, non-toxic or osteogenic. Bioactivity, degradation rate and tissue response depend on phase composition, crystallinity, porosity, surface chemistry, sintering conditions and the local biological environment [18]. In AM, these factors are especially important because slurry formulation, binder content, photopolymer content, thermal treatment and sintering shrinkage can alter pore architecture, mechanical reliability and biological response. Consequently, ceramic biomaterials are particularly attractive for bone repair, dental applications, coatings and mineralised-tissue scaffolds, but their brittleness, limited tensile toughness and processing-induced dimensional changes remain important constraints [18].

- Polymers:

Polymeric biomaterials are extensively used in biomedical applications because their chemistry, molecular architecture and processing conditions can be tailored to produce flexible, rigid, degradable or non-degradable systems [19,20,21]. They are employed in tissue-engineering scaffolds, drug-delivery systems, wound-care materials, temporary implants, prosthetic components, anatomical models and soft-tissue supports [20]. Nevertheless, biodegradability should not be presented as a general characteristic of synthetic polymers. Some polymers, such as poly(lactic acid), poly(glycolic acid), poly(lactic-co-glycolic acid) and polycaprolactone, are commonly investigated as biodegradable or bioresorbable materials, whereas others, such as polyether ether ketone, polymethyl methacrylate or ultra-high-molecular-weight polyethylene, are selected for longer-term stability [19,20,21,22]. Degradation behaviour depends on polymer chemistry, molecular weight, crystallinity, hydrophilicity, additives, sterilisation conditions, processing history and implantation environment [22]. In AM, thermal exposure, photopolymerisation, residual monomers, solvent residues, interlayer bonding and post-processing may further affect mechanical behaviour, cytocompatibility and biological performance [21,22].

- Composites:

Composite biomaterials combine two or more phases, such as polymer–ceramic, metal–ceramic, hydrogel–particle or fibre-reinforced systems, in order to tailor mechanical, biological, degradation or bioactive behaviour [23]. Their performance is not intrinsically superior to that of monolithic materials, but depends on the compatibility between matrix and reinforcement, interfacial bonding, particle dispersion, phase distribution, degradation mismatch and processing reproducibility [23,24]. In biomedical AM, composites are attractive because they may combine structural support with bioactivity, controlled degradation, improved osteoconductivity or tissue-mimetic behaviour [24,25]. However, they also introduce additional challenges, including heterogeneous microstructure, anisotropy, reinforcement agglomeration, interfacial failure, inconsistent degradation and more complex validation requirements. Their suitability must therefore be assessed in relation to the target tissue, intended biological function, manufacturing route and post-processing conditions [25].

To support a more direct comparison between the principal material families considered in this review, Table 2 summarises the main biomaterial classes used in AM for biomedical applications. The table compares metals and alloys, ceramics, polymers, hydrogels/bioinks and composites in terms of their mechanical behaviour, biocompatibility, printability, representative clinical applications and major limitations. This comparative overview highlights that no single biomaterial class is universally suitable for all AM-based biomedical constructs; instead, material selection must be aligned with the intended tissue environment, processing route, structural requirements, biological response and translational constraints.

Because the behaviour of each biomaterial class is strongly dependent on composition, processing route, architecture and biological environment, the following comparison should be interpreted as a class-level overview rather than as a universal ranking of material performance. Established material characteristics are distinguished from application-dependent observations wherever possible.

This process-oriented perspective is consistent with recent developments in AMed porous metallic implants, where manufacturing route, architectural design, surface engineering, corrosion behaviour and biological integration must be considered as mutually dependent variables. Bricha et al. [19] examined the challenge of reconciling porous metallic-implant design with mechanical reliability, osseointegration potential, surface functionalisation and long-term clinical durability. Their review reinforces the need to interpret biomaterial-based AM not as a set of isolated fabrication routes, but as a process–structure–property–translation framework in which material selection, architecture, surface condition, post-processing and biological response jointly determine clinical relevance.

Despite these advances, AM of biomaterials remains constrained by several cross-cutting challenges that affect both acellular and cellular fabrication routes. First, material–process compatibility remains difficult to establish because conditions that improve shape fidelity, resolution or mechanical stability may simultaneously compromise degradation behaviour, cytocompatibility or cell viability. Second, process–structure–property relationships are still insufficiently predictable, as powder morphology, feedstock rheology, thermal history, curing kinetics, layer orientation, pore architecture and post-processing can substantially modify surface condition, microstructure, porosity and biological response. Third, biological validation is often disconnected from manufacturing history, particularly in studies involving degradable, cell-laden or vascularised constructs. Fourth, the lack of standardised printability, biofunctionality and quality-assurance metrics limits reproducibility and makes comparison between studies difficult. Finally, translation from laboratory-scale protocols to clinically robust manufacturing workflows remains hindered by sterilisation compatibility, batch-to-batch variability, digital traceability, regulatory uncertainty, scale-up limitations and insufficient long-term in vivo evidence.

In response to these cross-cutting challenges, the distinctive contribution of this review is to move beyond a process-by-process description of AM techniques and interpret biomaterial-based AM as an integrated biomaterial–process–structure–property–translation ecosystem. Existing reviews commonly focus on individual material families, specific fabrication routes, isolated bioprinting strategies or selected biomedical applications. By contrast, the present review synthesises the field through the interdependence between biomaterial selection, material formulation, processing route, structure formation, post-processing, biological performance and clinical readiness. Rather than treating BJ, DED, ME, MJ, PBF, VAT photopolymerisation and bioprinting as independent process families, the review examines how each route mediates the relationship between printability, structural fidelity, mechanical competence, biological functionality, reproducibility and translational feasibility. This perspective is necessary because the major limitations of the field do not arise from fabrication capability alone, but from the difficulty of reconciling material–process compatibility, cell viability, biodegradation behaviour, regulatory compliance, standardised validation and long-term in vivo performance within the same construct. Accordingly, the review identifies cross-cutting knowledge gaps that remain insufficiently resolved across the literature, including the lack of standardised printability and biofunctionality metrics, the limited predictability of process–structure–property relationships, the incomplete validation of cell-laden and vascularised constructs, the insufficient integration of post-processing with biological outcomes and the weak transfer of laboratory-scale biofabrication protocols towards clinically robust manufacturing workflows.

The paper is organised into six sections. Section 1, Introduction, defines the scope of the study and establishes the rationale for reviewing AM of biomaterials from an integrated translational perspective. Section 2, Method of Research, describes the review design, including the search strategy, study-selection procedure and quality-assessment framework adopted for the analysis of the literature. Section 3, Literature Review, synthesises the principal additive-manufacturing routes applied to biomaterials, distinguishing between acellular techniques and cellular biofabrication approaches. Section 4, Discussion, critically evaluates the cross-route relationships, technical trade-offs and unresolved knowledge gaps associated with material–process compatibility, structural fidelity, post-processing, biological performance and clinical translation. Section 5, Conclusions, summarises the principal insights derived from the review. Finally, Section 6, Future Considerations, outlines prospective research directions related to smart biomaterials, artificial-intelligence-assisted optimisation, 4D printing, vascularisation strategies, standardisation, sustainability and translational validation.

Accordingly, the review is structured not only to describe AM routes, but to evaluate how material selection, processing conditions, post-processing, biological performance and translational constraints jointly determine the feasibility of AMed biomaterials for biomedical use. From a biomedical-engineering standpoint, quality assurance in AMed biomaterials should not be regarded merely as final-stage inspection. Instead, it must be embedded throughout the full development pathway, from biomaterial selection and feedstock preparation to process control, post-processing, biological validation and translational assessment. This is particularly important because construct performance depends not only on geometrical accuracy, but also on cell viability, degradation behaviour, mechanical competence, surface functionality, sterilisation compatibility and long-term biological response. Accordingly, the present review approaches quality assurance as an integrated requirement for clinically relevant biomaterial manufacturing, rather than as a separate verification activity performed after fabrication.

## 2. Method of Research

This review was conducted through a structured literature survey guided by the PRISMA 2020 framework [26,27], with the objective of ensuring transparency, traceability and reproducibility in the identification, screening and selection of relevant studies. Bibliographic records were compiled in January 2026, principally from CrossRef and complemented by Dimensions.ai to broaden retrieval and improve metadata completeness. The search focused on studies addressing the AM of biomaterials, including biomaterial classes, AM routes, bioprinting approaches, scaffold fabrication, post-processing, biological performance and translational applicability.

The search strategy was structured around three conceptual blocks: biomaterial systems, additive-manufacturing technologies and biomedical or translational performance criteria. The corrected Boolean logic retained “Biomaterials” as the anchoring term and followed the structure: “Biomaterials” AND (“Hydrogel Systems” OR “Metallic Implants” OR “Bioceramics” OR “Polymers” OR “Composites”) AND (“Additive Manufacturing” OR “3D Printing” OR “Bioprinting” OR “Binder Jetting” OR “Directed Energy Deposition” OR “Material Extrusion” OR “Material Jetting” OR “Powder Bed Fusion” OR “Vat Photopolymerisation”) AND (“Cell Viability” OR “Post-Processing” OR “Mechanical Performance” OR “Scaffold Fabrication” OR “Tissue Engineering” OR “Clinical Translation” OR “Precision Medicine”). Search queries were applied to title, abstract and keyword fields, and were limited to English-language peer-reviewed publications from 2010 to 2025.

Records were consolidated using a bespoke MATLAB^®^ R2024a routine for metadata standardisation, DOI-based duplicate detection, title/abstract/keyword-field harmonisation and preliminary eligibility filtering. Deduplication was performed primarily using DOI information, with secondary checks based on normalised title, abstract and keyword similarity where DOI information was unavailable, missing or inconsistently indexed. The MATLAB^®^ routine was used only to organise and reduce the database-level retrieval pool; it was not used to make final relevance, quality-assessment or inclusion decisions.

To improve reproducibility, the revised workflow distinguishes between four sequential stages: broad database retrieval, automated metadata-assisted filtering, manual title/abstract/keyword screening and full-text eligibility assessment. The largest number reported in the PRISMA workflow corresponds to the initial database-level output generated by the Boolean search, not to the number of records manually screened by the authors (Supplementary Table S1). Manual screening was performed only after deduplication, language restriction, document-type filtering, metadata cleaning and preliminary keyword-field eligibility checks.

Records were retained for manual screening when they: (i) were published between 2010 and 2025; (ii) were written in English; (iii) were classified as peer-reviewed journal articles or full review articles; (iv) contained sufficient title, abstract, keyword or DOI metadata for screening; and (v) addressed at least one biomaterial class in combination with an additive-manufacturing, 3D-printing, biofabrication or bioprinting process. Records were removed before manual screening when they were duplicates, incomplete metadata entries, non-English publications, conference abstracts without sufficient methodological detail, editorials, book chapters, patents, grey literature or records outside the biomedical and biomaterial scope of the review.

Exclusion criteria were applied sequentially during title/abstract/keyword screening and full-text eligibility assessment. Records were excluded when they met one or more of the following criteria:

(i) absence of a clear relationship between biomaterials and AM-based fabrication;
(ii) focus on conventional manufacturing without additive or biofabrication relevance;
(iii) absence of biomedical, tissue-engineering, implant, scaffold, regenerative-medicine or clinical-translation context;
(iv) insufficient information on material systems, processing route or biological/mechanical performance;
(v) Exclusive focus on non-biomedical industrial AM applications;
(vi) lack of accessible full text or inadequate methodological transparency;
(vii) duplicate or substantially overlapping records;
(viii) non-peer-reviewed publication type, abstract-only report, editorial, patent, book chapter or grey literature; and
(ix) studies outside the defined language or publication-year limits.

For additional information regarding the Method of Research, it is suggested to consult Appendix A.

**Figure 1:**
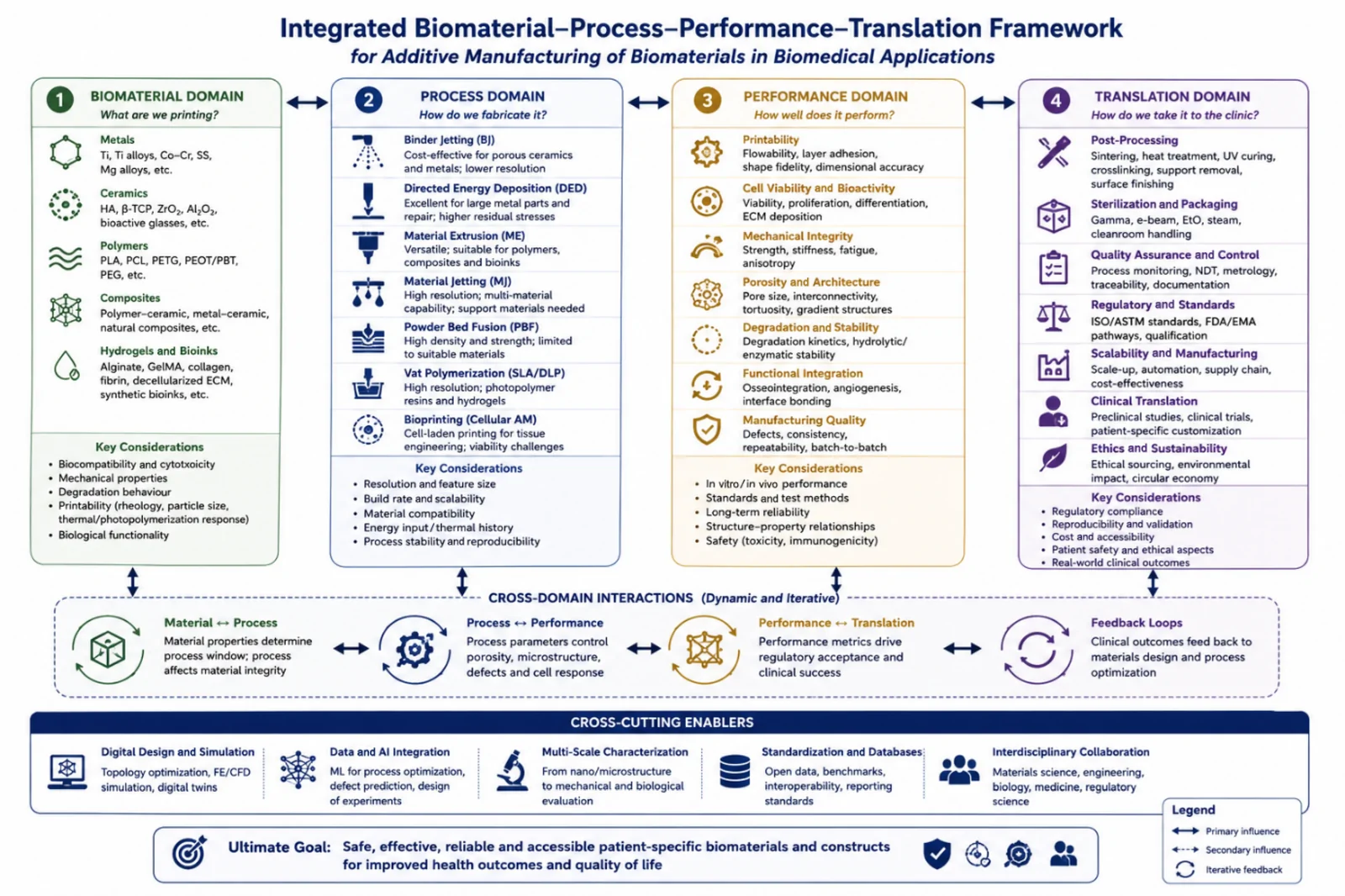
Integrated biomaterial–process–performance–translation framework illustrating the multidimensional interactions governing AM of biomaterials for biomedical applications (figure done in Microsoft^®^ Visio Pro 2021).

When multiple exclusion criteria applied, the record was assigned to the first applicable criterion in the screening sequence to avoid multiple counting. Based on the descriptive synthesis and recurring interdependencies identified throughout the reviewed literature, an integrated conceptual framework (Figure 1) was developed to consolidate the principal interactions between biomaterial selection, additive-manufacturing strategy, process performance and translational applicability. The framework aims to provide a systems-level interpretation of the multidimensional factors governing the successful development of AMed biomaterial constructs.

## 3. Literature Review

AM routes for biomaterials may be broadly distinguished according to whether they process acellular feedstocks or cell-containing biofabrication systems. However, this distinction should not be interpreted only as a technical classification. From a biomaterials perspective, the decisive issue is how each process route modifies the relationship between material chemistry, architecture, structural fidelity, post-processing exposure and biological response. Accordingly, the following sections do not aim to reproduce generic operational descriptions of established AM techniques. Instead, they evaluate the principal routes in terms of their relevance to biomedical constructs, with particular attention to material–process compatibility, cell viability, degradation behaviour, mechanical competence, surface functionality and translational suitability.

Accordingly, the literature is interpreted through recurring material–process–biology trade-offs rather than as a purely sequential catalogue of AM technologies. Particular attention is given to how biomaterial chemistry, feedstock state, rheology, crosslinking mechanism, thermal exposure, pore architecture, layer orientation and post-processing history determine structural fidelity, biological response and translational suitability.

To avoid treating the literature as a sequence of isolated studies, the following sections organise evidence according to recurring material–process–biology relationships. Individual studies are therefore discussed not only as examples of specific AM routes, but also as evidence of broader trends concerning printability, structural fidelity, post-processing dependence, biological performance, reproducibility and translational readiness.

**Figure 2:**
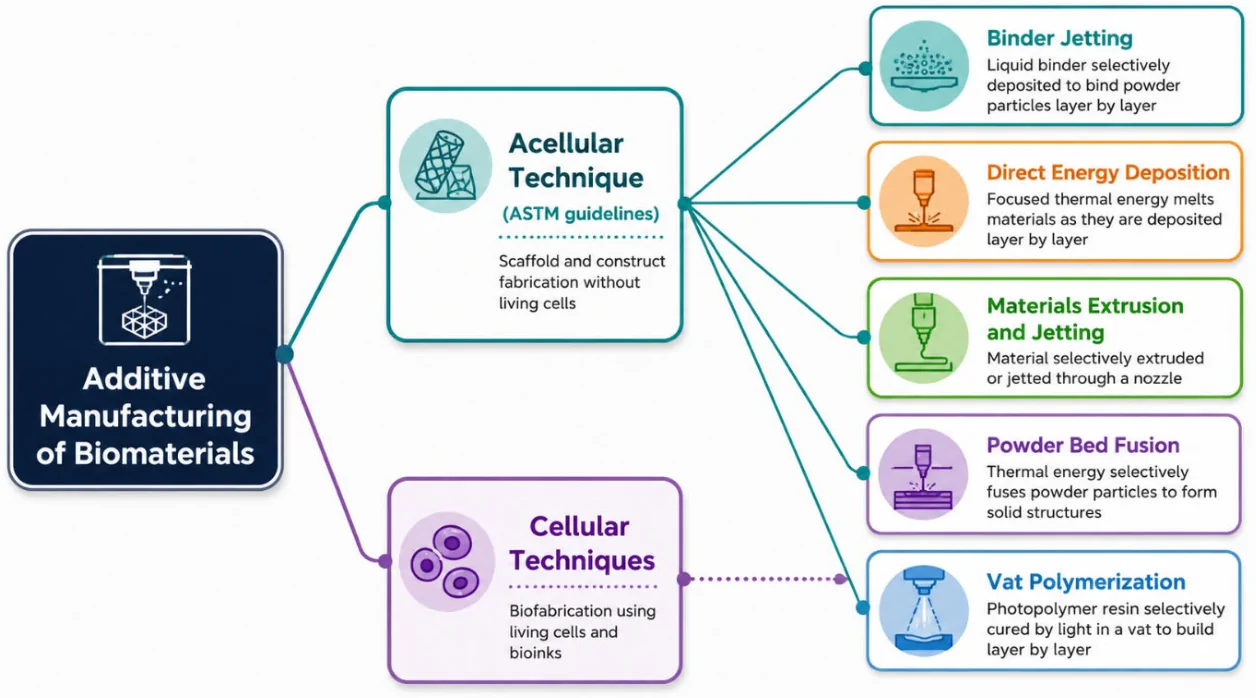
Classification of AM techniques for biomaterials (figure adapted in Microsoft^®^ Visio Pro 2021, from [28]).

Figure 2 and the schematic process figures onwards are therefore used as technical orientation aids rather than as standalone workflow illustrations. Their interpretation should be linked to the process-specific parameters discussed in the text, including feedstock state, energy or deposition mechanism, layer formation, thermal exposure, crosslinking or consolidation strategy, attainable resolution, post-processing requirements and biomaterial-specific consequences for structural fidelity, cytocompatibility, degradation behaviour and clinical translation.

There are two main categories of AM techniques for biomaterials: acellular and cellular approaches, as summarised in Figure 2. The acellular category involves printing materials without any live cells. The cellular category involves the printing of live cells along with other materials [28].

Because several AM technologies can be adapted to both acellular biomaterial fabrication and biologically oriented biofabrication, their fundamental principles are consolidated in Table 3 to avoid repetition across subsequent sections. The distinction adopted in this review is therefore not based solely on the machine architecture, but on whether the process is used to fabricate cell-free biomaterial constructs or to deposit cell-containing or biologically active formulations. Accordingly, Table 3 compares the main process families in terms of operating principle, material compatibility, suitability for hard-tissue and soft-tissue applications, clinical implant readiness, principal advantages and major limitations.

### 3.1. Acellular Techniques

Acellular AM routes remain the most technologically mature approaches for producing biomaterial constructs, particularly when the target application requires mechanically stable scaffolds, porous metallic implants, ceramic bone substitutes or patient-specific anatomical structures. Their principal advantage lies in the ability to process metals, ceramics, polymers and composites into geometrically controlled architectures without the additional constraints imposed by direct cell encapsulation. Table 3 adapted from [29] provides a comparative overview of the principal acellular AM routes, including compatible material classes, technical advantages, process limitations and representative biomedical applications. However, the table should be interpreted as a biomaterials-oriented synthesis rather than as a purely technological comparison: printing speed, attainable resolution, anisotropy, post-processing requirements, support needs and cost are relevant because they influence construct architecture, surface state, mechanical reliability, degradation behaviour and biological performance. For this reason, the following subsections focus on the biomedical implications of each acellular route rather than providing exhaustive descriptions of process operation.

Although the following subsections discuss each AM route separately for clarity, their interpretation should be comparative rather than sequential. Each process is therefore evaluated according to how it mediates the relationship between biomaterial compatibility, structural fidelity, mechanical performance, biological response, post-processing burden and translational readiness.

Table 3 highlights that the suitability of each AM technology is strongly application-dependent and cannot be assessed only through general advantages and disadvantages. PBF and BJ are particularly relevant for hard-tissue-oriented applications because they can process metallic, ceramic and calcium-phosphate-based materials into porous or mechanically resistant structures; however, both routes remain strongly dependent on post-processing to ensure dimensional stability, mechanical reliability and biological acceptability. DED is more appropriate for metallic repair, coatings and functionally graded biomaterial structures than for fine scaffold fabrication or cell-compatible processing, mainly because of its high thermal input and comparatively limited resolution. By contrast, ME, MJ and vat photopolymerisation are more closely aligned with polymeric, hydrogel-based and soft-tissue-oriented constructs, although their biomedical use depends on controlling rheology, curing conditions, cytocompatibility, cell damage and post-processing effects. Therefore, the selection of an AM route should be guided by the target tissue environment, required mechanical function, biological content, post-processing burden and expected translational pathway, rather than by process speed, resolution or material compatibility alone.

#### 3.1.1. Binder Jetting (BJ)

BJ is defined as an AM process in which a liquid bonding agent is selectively deposited onto successive powder-bed layers to join powder particles and form a green part, in accordance with the ASTM F2792-12 standard [30]. In biomaterial applications, BJ is particularly relevant for ceramic, calcium-phosphate and selected metallic powders because it can generate porous architectures without the high thermal gradients imposed by beam-based fusion routes. However, the resulting green parts usually require curing, infiltration, sintering or other post-processing steps to achieve adequate cohesion, dimensional stability and mechanical integrity.

In biomedical applications, the relevance of BJ lies primarily in its capacity to process ceramic, calcium-phosphate and selected metallic powders into porous constructs without imposing severe thermal gradients during initial shaping. However, its biological value depends strongly on post-processing. Low green strength, residual binder, pore interconnectivity, dimensional variation and sintering- or infiltration-induced microstructural changes may directly influence compressive behaviour, degradation rate, tissue ingrowth and inflammatory response. Consequently, BJ should be evaluated not only by build speed or geometrical freedom, but by the extent to which post-processing preserves clinically useful porosity while providing adequate mechanical stability and biological safety.

BJ can utilize a diverse array of materials like sand, ceramics and metals, which can be processed into suitable powders for binder interaction. Notably, this technique is applicable to both dry and wet powders, each having distinct requirements. Despite the variations, there are two crucial factors that can significantly impact the BJ printing process: powder size and shape. The size of powder particles significantly affects powder flowability. Generally, larger particles are preferred in dry BJ due to their excellent flowability and low surface area. Fine powder is typically applied as a slurry because it easily absorbs moisture, leading to agglomeration and impacting powder flowability [31]. Apart from its effects on powder flowability, powder size also plays a substantial role in determining the properties of the final parts. The use of fine powder in BJ can enhance the density of final parts after post-processing, such as sintering. Additionally, fine powder BJ tends to result in better surface finishing and lower surface roughness. Powder shape has a lesser influence on BJ compared to particle size. However, in dry BJ, spherical powders are preferred due to their superior flowability and lower friction compared to faceted or anisotropic powders [32]. Gharraei et al. [33] focused on the challenge of clarifying how bioink rheology, nozzle-flow mechanics, and process-induced stresses jointly govern printability, extrusion stability and cell damage. The authors delivered a comprehensive fluid-mechanics-based review linking viscoelasticity, thixotropy, extensional stresses and modelling approaches to improved extrusion-bioprinting control and scaffold quality.

Although Table 3 summarises the principal advantages and limitations of the main acellular AM routes, the relevance of these limitations depends on how process outputs translate into biomedical performance. In BJ, the absence of a high-temperature energy source is advantageous for brittle ceramic or composite powders, but the resulting green bodies typically require curing, infiltration or sintering to improve cohesion and mechanical integrity. Consequently, porosity, dimensional change and residual binder content become decisive parameters for implant reliability and biological response. In PBF, dense metallic structures can be obtained with high mechanical strength, but thermal gradients, residual stresses, unmelted particles and rough surfaces may influence fatigue performance, ion release, osseointegration and inflammatory behaviour. In extrusion-based systems, process accessibility and material versatility are counterbalanced by relatively low resolution, interlayer anisotropy and shear-induced damage when cell-laden or hydrogel-based feedstocks are used. Similarly, vat photopolymerisation and MJ provide high resolution and architectural control, but residual photoinitiators, unreacted monomers, ultraviolet exposure and post-curing conditions may compromise cytocompatibility. Therefore, the evaluation of AM routes for biomaterials requires simultaneous consideration of processing accuracy, structural integrity, post-processing burden and biological safety, rather than a purely technological comparison of fabrication principles.

There are two categories of binders—organic and inorganic. An organic binder connects the powder during the curing process, while the inorganic binder forms a bond through colloid gel formation [34]. Binders can also be categorised as acid–base binders, metal salts binders and solvent binders. Acid–base binders regulate powder bonding through acid–base reactions. Metal salts binders typically create bonds with the powder through salt recrystallisation, reduction of salt crystallisation, or salt displacement reactions. Solvent binders are designed for polymeric powders, dissolving the deposited area, and forming a designed structure after solvent evaporation. Additionally, based on various binding mechanisms, there are in-bed binders, phase-changing binders and sintering inhibition binders. In-bed binders differ from regular liquid binders in their source, as they mostly mix with the powder in the bed and then bind with the powder through liquid jetting from the nozzle. Examples include plasters and cement, where binding occurs via hydration-activated bonds when liquid is jetted onto the powder bed [20]. Phase-changing binders work by solidifying to hold the powder together. Lastly, sintering inhibition binders can control the sintering area by selectively jetting heat-isolating material [35,36].

The printing process involves injecting the binder from the jetting head to the build stage for selective binding. The machine consists of four parts: feed stage, levelling roller, build stage and BJ head [37]. The feed stage handles dry powder or slurry for powder feeding, with dry powder requiring compactness for smooth and uniform layer spreading. Levelling rollers transfer the powder from the feed stage to the build stage, with various types available for different applications. Liquid slurry, spread by slip casting, has higher packing density but poses a longer build time and a risk of cracking [31].

**Figure 3:**
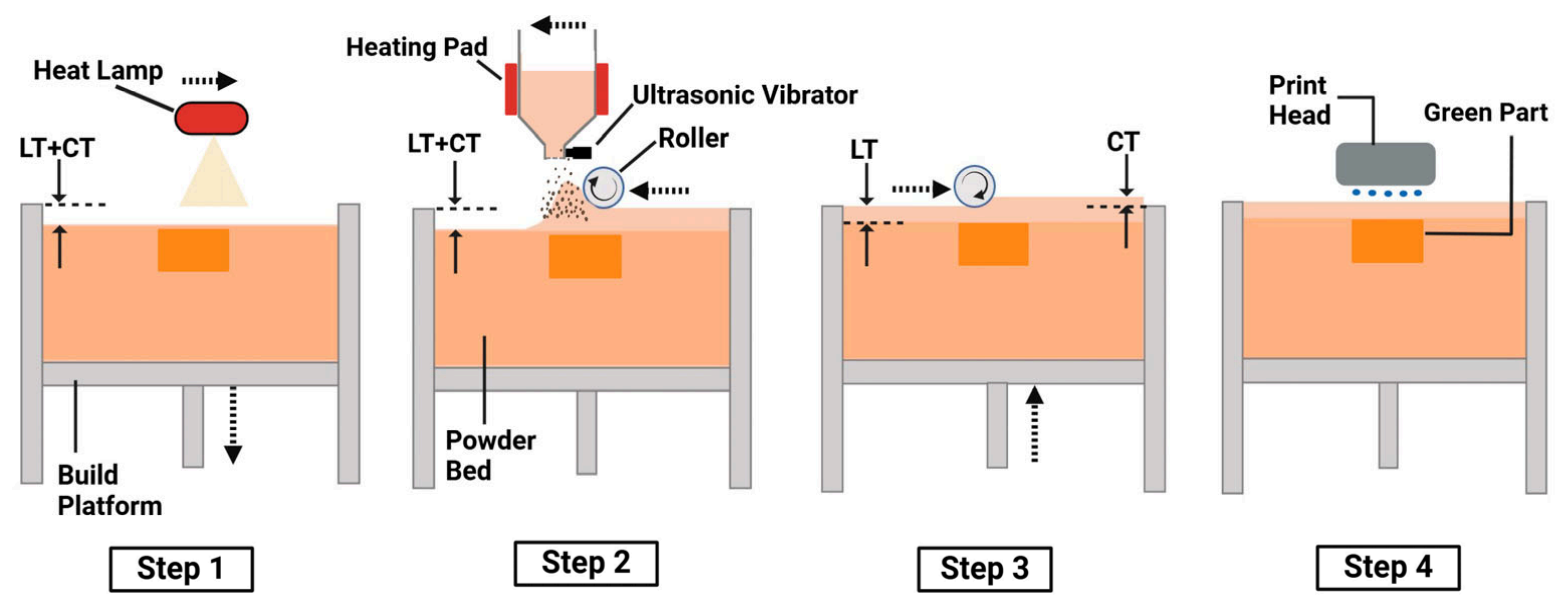
The schematic of the BJ machine (Reprinted from [38]).

The printing of the designed structure utilizes two types of print heads: drop-on-demand (DoD) and continuous-jet (CJ). DoD print heads, such as thermal ink jet and piezoelectric, dispense liquid drops on demand, while CJ print heads enable continuous ink jetting. The build stage descends for each layer of printing, and binder saturation level, influenced by droplet spacing, size, bed packing density and layer thickness, must be balanced to avoid uneven surfaces or insufficient binder penetration. Adequate drying time is crucial to prevent cracking, and the preferred layer thickness should be at least three times the powder particle size [31]. The visual representation of the printing process can be seen in Figure 3.

The critical technical variables represented by this workflow include powder particle size and morphology, layer thickness, binder saturation, droplet spacing, drying time, depowdering strategy and post-print consolidation, all of which influence dimensional fidelity, residual porosity, green strength and biological suitability.

After 3D printing, the post-printing process is crucial for enhancing the bonding between powder and binder to improve the green strength of parts. For polymer binders, curing through further polymerisation without affecting the loose powder is a viable solution [28]. Drying is another method, particularly effective for cement and plaster-based materials, as it eliminates excess liquid in the part [32].

Huang et al. [39] identified the principal challenge as overcoming the inherently low strength of highly porous binder-jetted hydroxyapatite parts, while avoiding post-sintering shrinkage, deformation and loss of bone-relevant organic functionality. Using 5 wt% citric-acid post-processing markedly thickened the PVA interparticle film, raising compressive strength and modulus to trabecular-bone-relevant levels through a simple room-temperature route. Martínez et al. [40] examined the challenge of controlling dimensional variation during resin infiltration of binder-jetted parts, particularly the effects of bed position, density heterogeneity, drying history and immersion time. It was demonstrated that pre-drying and appropriate bed placement improve resin uptake while limiting geometric deviation, with dimensional changes remaining modest and greatest along the build direction.

Other options include selective sintering with sintering inhibitors, reduction of a salt-based binder, and pre-ceramic polymer conversion [31]. Once the part attains sufficient mechanical strength, depowderisation is carried out using methods such as vibration, vacuuming, and wet depowderisation [41]. Additional processes like microwave boiling, carbon dioxide bubbling, and ultrasonication can assist with wet depowderisation. Following depowderisation, parts undergo heat treatment or sintering to enhance density and mechanical properties. Conventional and microwave sintering are common methodologies, with microwave sintering showing significant improvements in density and compressive strength for porous tricalcium phosphate (TCP) parts compared to conventional sintering [42]. Some sintered parts undergo infiltration with a second material to further enhance mechanical properties, categorised as low-temperature (polymers like epoxy and polycaprolactone) and high-temperature (alloys) processes. For instance, SiC–Si composite parts are prepared by carbon powder printing followed by Si infiltration at 1450 °C with vacuum assistance. In addition to mechanical improvement, infiltration offers a new approach to fabricate composite parts [32].

Salehi et al. [43] addressed the difficulty of producing biodegradable magnesium scaffolds with bone-mimetic interconnected microporosity while retaining the mechanical stiffness and strength required for load-bearing hard-tissue applications. Binder-jetted Mg–Zn–Zr scaffolds achieved cortical-bone-relevant mechanical properties, >95% pore interconnectivity, and superior cytocompatibility and osteoblastic response relative to cast alloy. Salehi et al.’s [44] study addressed the absence of a robust end-to-end digital workflow for personalised magnesium implants, particularly the combined control of sintering shrinkage, machining safety, dimensional fidelity and surface quality. A hybrid binder-jetting and automated dry-machining route was established, producing personalised porous Mg implants with controlled shrinkage, preserved internal porosity and defect-free surfaces. Kuah et al. [45] addressed the difficulty of mitigating corrosion in binder-jetted porous magnesium scaffolds, where high surface area, constrained pore geometry and localised electrochemical heterogeneity undermine coating effectiveness. Vacuum-assisted hydroxyapatite and phosphate coatings penetrated porous scaffolds, with hydroxyapatite delivering the most pronounced reduction in hydrogen-evolution-based corrosion rate. Zhou et al. [46] study focused on the poor reactivity of hydroxyapatite with standard water-based binders, while seeking to reconcile printability, geometrical fidelity, de-powdering behaviour and green mechanical strength. The authors demonstrated that 30 wt% high-molecular-weight poly(vinyl alcohol) (PVOH) enabled hydroxyapatite-rich scaffolds with >85% geometrical accuracy and a green compressive strength of 5.63 MPa.

Taken together, these studies show that the biomedical relevance of BJ is governed less by the printing step alone than by the interaction between powder characteristics, binder chemistry and post-processing strategy. The reviewed evidence consistently indicates that BJ is attractive for porous ceramic, calcium-phosphate and biodegradable metallic constructs because it enables architectural complexity without direct thermal fusion during shaping. However, the same studies also show that mechanical reliability, dimensional stability, corrosion behaviour and biological response remain strongly dependent on curing, sintering, infiltration, coating or post-treatment conditions. Therefore, BJ should be interpreted as a powder–binder–post-processing route in which clinical suitability depends on preserving biologically useful porosity while achieving adequate strength, degradation control and cytocompatibility.

***Critical Technological Limitations***—BJ

Despite its remarkable capability for fabricating highly porous and geometrically complex biomaterial architectures, BJ still presents several critical technological limitations that constrain its broader translational applicability in advanced biomedical engineering. One of the most significant challenges concerns the inherently low green strength of printed components prior to sintering or infiltration, making constructs highly susceptible to cracking, deformation, and handling-induced damage during post-processing operations. Furthermore, the absence of localised melting mechanisms frequently results in incomplete particle bonding, elevated residual porosity, and limited mechanical reliability, particularly in load-bearing biomedical applications.

Dimensional instability during debinding and sintering also remains a major concern, as nonuniform shrinkage may compromise geometrical fidelity, pore interconnectivity, and implant reproducibility. In addition, the dependence on powder characteristics, binder saturation, particle-size distribution, and spreading homogeneity introduces substantial process variability, reducing manufacturing consistency and batch-to-batch reproducibility. The depowdering stage constitutes another important bottleneck, especially for highly intricate porous structures where trapped residual powder may obstruct internal channels or compromise biological functionality.

From a translational perspective, BJ additionally faces important limitations regarding process standardisation, real-time quality monitoring, and regulatory validation, particularly for patient-specific biomedical devices requiring strict dimensional precision and long-term structural reliability. Consequently, although BJ remains highly attractive for porous scaffold fabrication and bioinspired architectures, its future clinical scalability will strongly depend on advances in powder engineering, binder chemistry, process monitoring, densification strategies, and integrated quality-control methodologies.

From a biomedical standpoint, the main limitation of BJ is not only the low strength of as-printed parts, but the difficulty of improving mechanical performance without compromising dimensional fidelity, porosity architecture or biological acceptability. In ceramic and calcium-phosphate systems, post-processing may increase compressive strength and stiffness, but excessive sintering or infiltration can reduce interconnected porosity, alter degradation behaviour and affect the surface features required for bone ingrowth. Conversely, insufficient post-processing may leave fragile constructs with inadequate load-bearing capacity. Binder selection is also biologically relevant, since residual organic phases or incomplete removal of processing additives may influence cytocompatibility, local inflammatory response and early tissue integration. For this reason, binder-jetted biomaterial constructs should be assessed through coupled mechanical, microstructural and biological metrics, including porosity distribution, compressive behaviour, surface chemistry, degradation response and cell compatibility.

#### 3.1.2. Direct Energy Deposition (DED) Techniques

In techniques based on direct energy, a primary energy source, typically a laser or electron beam, is employed to deposit, melt, and solidify the material. This material is introduced either in powder form or as a filament or wire [47]. The process relies on the continuous supply of the initial material, whether in powder or filament form, which is then melted by the energy source and deposited layer by layer. A computer-controlled XYZ system oversees the part’s geometry and construction pattern in these methods. While these processes are primarily used for metals, there have been instances where they have been applied to polymeric, ceramic and composite materials as well. To enhance the categorisation of different methods within this category, diverse approaches are classified either as techniques for surface modification or for 3D part fabrication. Surface modification can be accomplished through AM processes to enhance the material’s surface performance, while 3D part fabrication refers to the manufacturing of near-net-shaped functional components [28]. Sorkhi et al. [48] investigated the post-processing challenge in LPDED WE43 magnesium alloy, namely reducing residual porosity while avoiding abnormal grain growth, heterogeneous precipitate dissolution and non-uniform heat-treatment response. It was demonstrated that HIP reduced porosity from roughly 2% to below 0.1%, while clarifying precipitate evolution and preserving an overall equiaxed, randomly oriented microstructure. Mani et al. [49] addressed the difficulty of developing a biocompatible, printable titanium–diamond feedstock capable of delivering structurally sound parts despite demanding flowability, integrity and parameter-optimisation constraints. Optimised laser metal deposition produced high-integrity TiD parts, with 30% diamond enhancing osteoblast viability and reducing bacterial burden relative to titanium.

**Figure 4:**
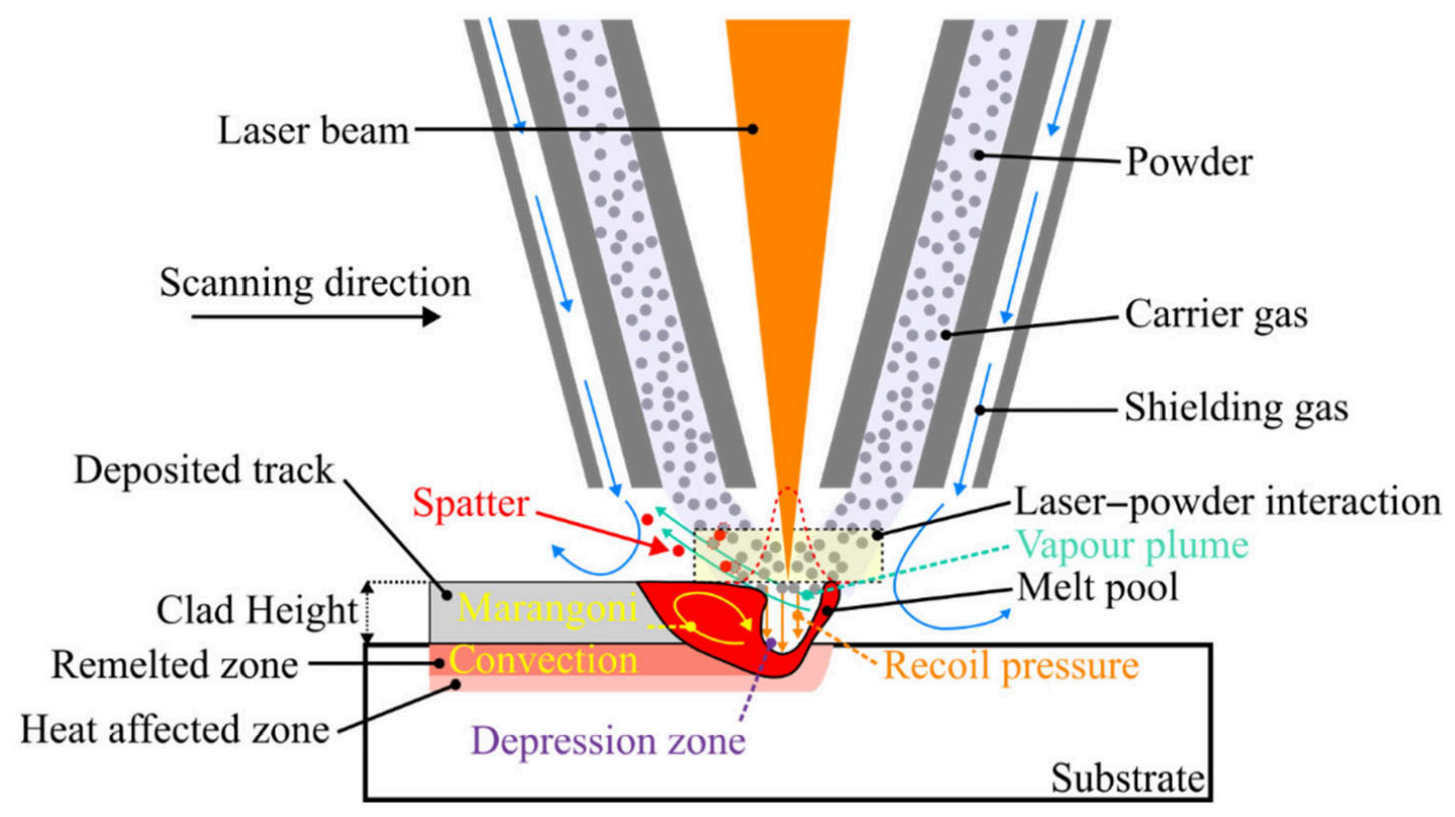
Schematic diagram of the laser cladding process with powder injection (Reprinted from [50]).

Laser melt particle injection represents an early precursor to modern powder-based direct energy AM. Ayers et al. demonstrated the micro-melting of the surface of a metallic substrate, allowing fine ceramic particles to be injected into the resulting micro-melt pool. Like laser melt injection, where powder is injected into the micro-melt pool, laser cladding can also be performed [28]. Both laser melt injection and laser cladding can utilize material feed in the form of powder, wire, or filament. Figure 4 illustrates typical laser cladding using a powder feed too. Employing computerised motion control, material deposition can occur along the X, Y, and Z axes, as well as on rotating parts, both internally and externally.

The key process features represented in this schematic include feedstock delivery, beam–material interaction, melt-pool geometry, thermal gradients, shielding conditions and layer-by-layer solidification, which collectively determine residual stress, surface roughness, microstructural heterogeneity and post-processing demand. Direct laser deposition techniques involve using a material feed, either in powder form or as a wire/filament, to build a three-dimensional part. These methods can also selectively modify the surface of materials, commonly known as laser powder forming or laser-based metal deposition (LBMD) [51].

Various powder feeders are available, allowing a blend of powders to be delivered through Argon pressurised nozzles. The powder is directed through multiple nozzles to a converging point, which coincides with the laser’s focal point. Upon delivery, the powder undergoes melting, creating a micro-melt pool. This melt pool adheres to a moving substrate or base plate along the X and Y axes. As the base plate moves, the system forms a line of molten material that rapidly solidifies, producing a cross-sectional layer. After completing a layer, the nozzle and laser assembly move upward along the Z direction by one layer thickness, depositing another layer atop the previous one. This layer-by-layer deposition process repeats, resulting in the creation of a three-dimensional (3D) component based on the original CAD file [52].

Electron Beam Direct Manufacturing (EBDM) involves the use of an electron beam to melt materials and deposit them in a 3D pattern, like the direct laser deposition technique. In the EBDM process, materials, provided in the form of a wire or filament, are melted by the direct energy of the electron beam. This process is conducted in a high vacuum environment to prevent the ionisation of atmospheric gases. Unlike laser-based methods that often use inert gases like argon, the vacuum in EBDM eliminates contaminants such as oxygen or nitrogen from entering the material [28].

Various processes falling under the direct energy deposition technique in AM have been successfully demonstrated for a range of metallic materials, including steels, superalloys, Co-based alloys, Ti-based alloys and bulk metallic glasses. Additionally, sensitive materials like pure Ti, Si, Ta, W and Zr, ceramic materials such as alumina, zirconia, different carbides and metal–ceramic composites have also been effectively showcased [53]. Beyond the diverse material systems these techniques can process, they have been employed for creating structurally graded materials with varying porosity in different component parts [54], compositionally graded materials and the manufacturing of multi-material systems [55,56].

Overall, the studies reviewed for DED indicate that this technique is most relevant for metallic biomaterials, surface modification, repair strategies and functionally graded implant structures, rather than for delicate cell-compatible or hydrogel-based systems. Its main biomedical value lies in the ability to process high-performance metallic materials, introduce compositional gradients and modify implant surfaces. Nevertheless, the evidence also highlights recurring limitations associated with high thermal input, residual stress, microstructural heterogeneity, surface roughness and the need for subsequent finishing. Consequently, DED should be considered a specialised route for metallic biomaterial reinforcement and functionalisation, rather than a universal scaffold-fabrication process.

***Critical Technological Limitations***—DED

Despite its exceptional flexibility for fabricating large-scale metallic structures, repairing high-value components and producing functionally graded biomaterials, DED still exhibits several critical technological limitations that significantly constrain its precision, reproducibility and translational reliability in advanced biomedical applications. One of the most critical challenges arises from the highly localised and unstable thermal cycles inherent to the process, which frequently generate steep thermal gradients, residual stresses, anisotropic microstructures and distortion phenomena capable of compromising dimensional accuracy and long-term structural integrity.

The complex melt-pool dynamics associated with powder- or wire-fed deposition additionally introduce significant variability in layer morphology, dilution behaviour and interlayer bonding quality. Such instability frequently results in heterogeneous microstructural evolution, nonuniform grain growth, elemental segregation, and localised porosity, particularly in complex geometries or multi-material constructs. Furthermore, the relatively coarse deposition resolution compared with PBF technologies substantially limits the fabrication of fine-feature biomedical architectures requiring highly controlled pore morphology and intricate internal geometries.

Surface quality also remains a major technological bottleneck in DED-produced biomaterials. The elevated surface roughness generated during deposition often necessitates extensive post-processing operations, including machining, polishing, thermal treatment and stress-relief procedures, thereby increasing manufacturing complexity, production time, and overall cost. In biomedical environments, these surface irregularities may additionally influence cell adhesion behaviour, osseointegration performance, corrosion resistance and fatigue reliability.

From a translational perspective, DED also faces important challenges regarding process monitoring, closed-loop control, repeatability, and certification for patient-specific medical devices. The strong sensitivity of the process to deposition parameters, feedstock characteristics, shielding conditions and thermal accumulation effects complicates standardisation and regulatory validation. Consequently, although DED remains highly promising for customised metallic implants, repair applications and functionally graded biomaterials, its broader clinical implementation will strongly depend on advances in real-time monitoring systems, predictive thermal modelling, adaptive process control and multiscale microstructural optimisation strategies.

From a biomaterial’s perspective, the main limitation of DED is its high thermal input and relatively low geometrical resolution compared with other AM routes. These characteristics restrict its use for delicate polymeric, ceramic or cell-compatible systems, but make it relevant for metallic biomaterials, graded structures, coatings and repair-oriented strategies. In implant-related contexts, residual stresses, microstructural heterogeneity, dilution effects, surface roughness and the frequent need for machining or surface finishing may affect fatigue resistance, corrosion behaviour, ion release and osseointegration. Therefore, DED should be interpreted less as a general scaffold-fabrication method and more as a specialised route for metallic biomaterial modification, reinforcement or functionally graded implant architectures.

#### 3.1.3. Material Extrusion and Jetting (ME and MJ)

**Figure 5:**
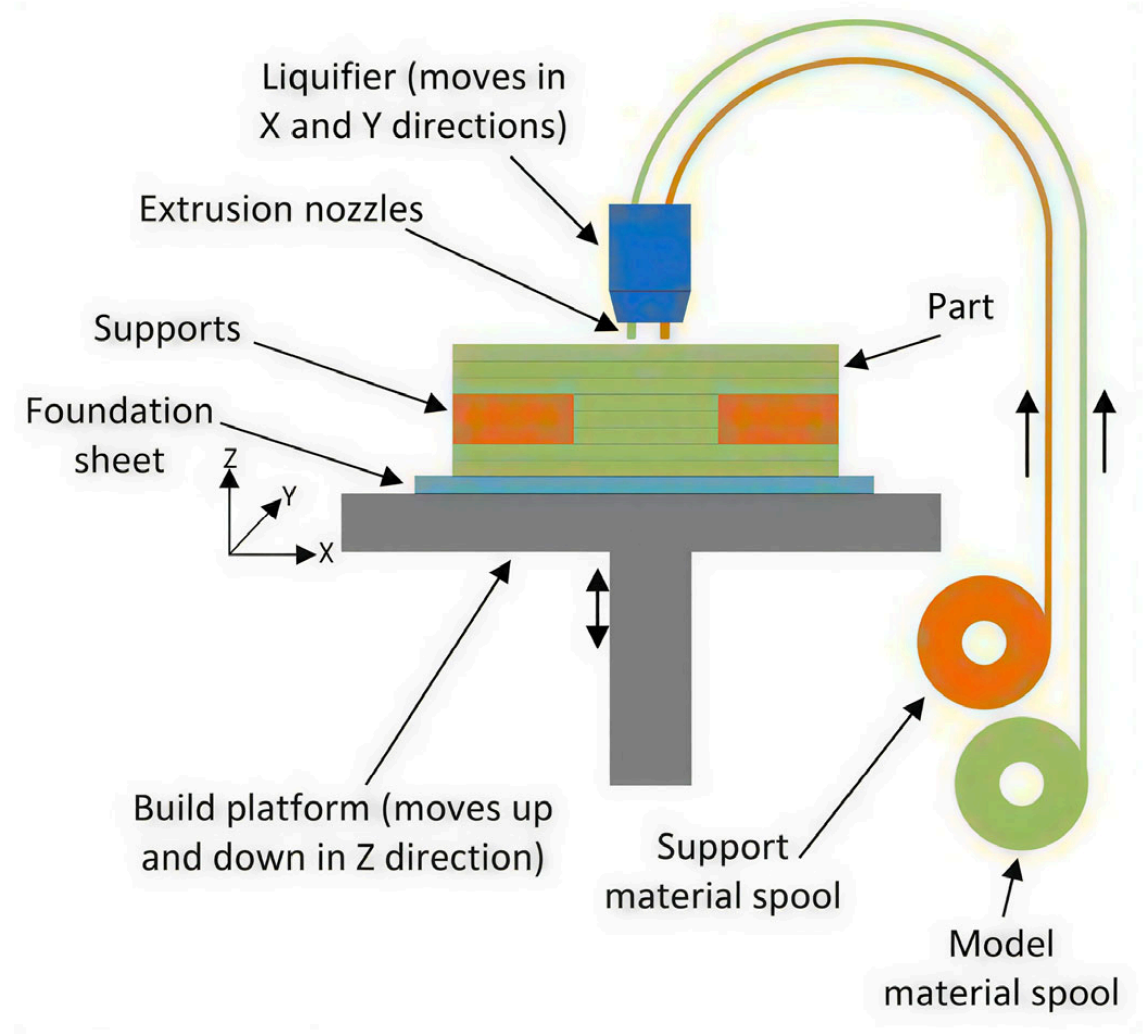
FDM process schematic(Reprinted from [64]).

In this method, a 3D part is created by utilising ME, commonly known as FDM [57]. FDM technology is primarily used for direct 3D printing of plastics or polymeric materials, given the comparatively lower softening temperature of polymeric materials compared to metallic or ceramic ones [58]. FDM involves heating the material until it becomes fluid, particularly in thermoplastic polymers, where the material softens during heating and can be extruded through a nozzle. The extrusion rate and resolution are adjusted for different builds using a predetermined nozzle. By extruding the material layer by layer in consecutive raster scans, a 3D part based on a CAD design is formed. Figure 5 illustrates a schematic of the FDM process. A modified version of FDM, known as fused deposition of ceramic (FDC), has also been applied to process green ceramic structures [59]. Due to material limitations, FDM technology is restricted to fabricating polymer parts. However, indirect FDM can be used to prepare ceramic constructs, as reported by [60]. Initially, a polymer mould with a honeycomb structure is created using FDM. Subsequently, the mould is infiltrated with ceramic slurry, followed by sintering to remove the mould.

McCauley et al. [61] explored the persistent difficulty of predicting cell damage during extrusion bioprinting, where process parameters, bioink rheology and cell mechanics interact in a highly coupled manner. A critical-strain-based viability model was developed integrating nozzle geometry, flow conditions, rheology and single-cell mechanics, thereby providing data-driven guidance for bioprinting optimisation. Malekpour and Chen [62] examined the fundamental challenge of jointly optimising printability and cell viability in extrusion bioprinting, given the competing influences of rheology, pressure, nozzle geometry and process conditions. The authors delivered an integrated experimental, computational and machine-learning review that clarified the principal governing parameters and highlighted data-driven optimisation as a promising route for improved bioprinting performance. Mendoza-Cerezo et al. [63] investigated the central challenge of reconciling GelMA printability and rheological stability with the incorporation of culture medium, without unduly compromising extrusion fidelity or cell viability. Culture-medium-modified GelMA formulations, particularly GelMA 75%, retained acceptable printability while supporting viable cell-laden bioprinting for tissue-regeneration-oriented scaffold fabrication.

**Figure 6:**
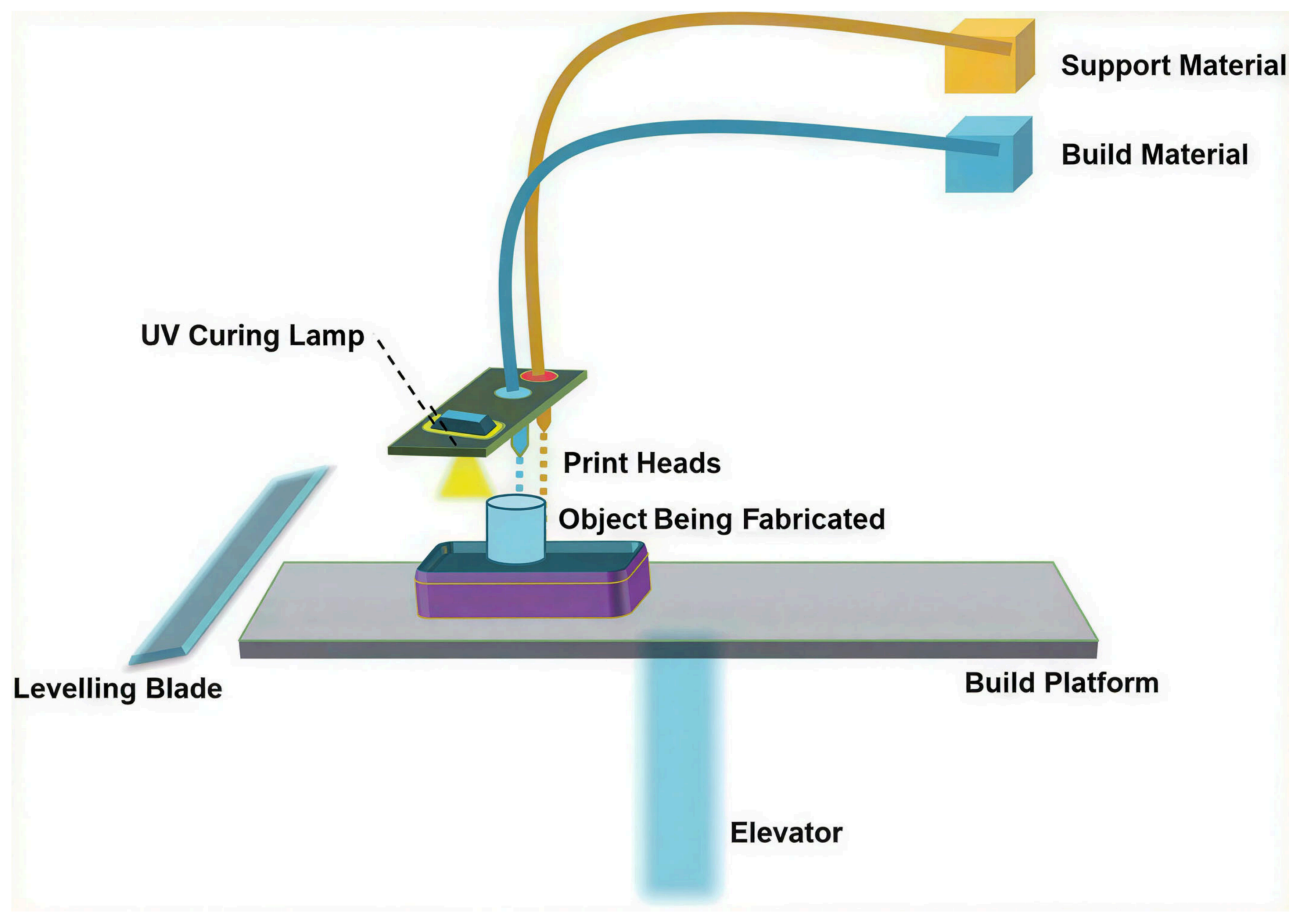
Schematic representation of MJ (MultiJet or PolyJet) process (reprinted from [65]).

The main parameters associated with this workflow include nozzle diameter, extrusion temperature or pressure, feed rate, layer height, raster strategy, cooling or gelation behaviour and interlayer bonding, which influence anisotropy, shape fidelity, shear exposure and scaffold reproducibility. Diverging from AM through ME, the process of MJ comes into play. In MJ, polymeric material droplets are dispensed onto a powder bed to create a 2D pattern. These deposited droplets are promptly cured using ultraviolet radiation. This method allows for the formation of fine layers, and the process is iterated for numerous layers to produce a complete 3D part. Stratasys Inc. introduced this technology in 2007 under the name PolyJet. Additional nozzles are incorporated for jetting droplets of secondary materials to serve as support, akin to the support structures generated in FDM processes. Notably, PolyJet technology (see Figure 6) has demonstrated the ability to produce combinations of different materials, a feat previously unattainable with FDM technology [65].

Kotlarz et al. [66] addressed the challenge of manufacturing scalable cell–hydrogel–microfibre composites, while preserving homogeneous cell incorporation, strong cell–biomaterial interactions and sufficient structural functionality for laminar tissues. They demonstrated that reactive jet impingement bioprinting produced highly viable fibroblast-laden hydrogel–microfibre composites, promoting proliferation, organisation, infiltration and tissue-precursor formation for wound-healing applications. Takagi et al. [67] addressed the challenge of achieving stable, high-precision inkjet bioprinting of living cells, while preventing sedimentation, nozzle clogging, viability loss and poor three-dimensional structuring. The authors developed a dedicated live-cell inkjet printhead enabling reliable drop-on-demand ejection, preserved cell functionality, precise multicellular patterning and finely stratified three-dimensional constructs. Hendriks et al. [68] investigated the difficulty of controlling post-impact cell viability in droplet-based deposition, where droplet size, impact velocity, liquid viscosity and substrate stiffness interact unfavourably. An analytical model was developed and validated linking membrane deformation to survival, thereby enabling rational optimisation of spraying and broader droplet-based biofabrication processes.

The technical relevance of this workflow lies in droplet formation, jetting frequency, viscosity range, impact behaviour, curing conditions, support-material removal and multi-material alignment, which affect resolution, surface quality, cell compatibility and construct size. Calore et al. [69] review identified persistent limitations in melt-based scaffold fabrication, notably restricted process control, inconsistent construct quality, limited biomimicry and barriers to standardisation and industrial upscaling. The article provided a focused critical synthesis of AM for hard-tissue scaffolds, clarifying key workflow bottlenecks and proposing strategies to improve control, reproducibility and biomimetic fidelity. Dixit et al. [70] addressed the difficulty of simultaneously achieving rheologically stable extrusion, adequate scaffold interconnectivity, enhanced mechanical competence and cytocompatibility in graphene-reinforced bioactive glass systems. It demonstrated that graphene oxide reinforcement markedly improved compressive strength, preserved bone-relevant porous architecture, and supported favourable myoblast viability in AMed scaffolds. Zarei et al.’s [71] study addressed the difficulty of combining printability, dimensional fidelity, mechanical reinforcement, and osteogenic functionality within PLA-based scaffolds intended for bone repair. Ti6Al4V at calcium phosphate core–shell reinforcement markedly improved scaffold mechanics, cell response, and osteogenic differentiation in AMed constructs. Cámara-Torres et al. [72] addressed the challenge of fabricating highly loaded nanohydroxyapatite–polymer scaffolds with retained printability and mechanics, while clarifying whether high nHA content alone can induce osteogenesis. It was found that 45 wt% nHA scaffolds enhanced compressive properties and promoted calcium phosphate layer formation, indicating promising long-term bioactivity and osteoconductive potential.

The literature on ME and MJ reveals a common trade-off between process accessibility, material versatility and biological sensitivity. Extrusion-based systems are attractive because they can process thermoplastics, hydrogels, composites and bioinks using relatively accessible equipment, but their performance is strongly constrained by nozzle diameter, extrusion pressure, rheology, interlayer bonding and shear-induced cell damage. MJ offers higher patterning precision and multi-material capability, but remains limited by viscosity constraints, droplet impact effects, material availability and construct scale. Collectively, these studies indicate that extrusion and jetting routes should be assessed through coupled printability and biological metrics, rather than by resolution or deposition accuracy alone.

***Critical Technological Limitations***—ME

Despite its widespread accessibility, low operational cost and remarkable versatility for processing thermoplastics, composites, hydrogels and bioactive materials, ME still presents several critical technological limitations that restrict its applicability in high-performance biomedical engineering. One of the most significant challenges concerns the inherently anisotropic nature of layer-by-layer deposition, which frequently generates weak interlayer adhesion, heterogeneous mechanical behaviour, and reduced structural reliability under complex loading conditions. The dependence on thermal fusion and extrusion rheology additionally promotes variability in filament continuity, dimensional fidelity, and pore reproducibility, particularly in highly porous scaffold architectures.

The relatively limited spatial resolution associated with nozzle-based deposition also constrains the fabrication of micro-scale features and highly intricate geometries required for advanced tissue-engineering applications. Furthermore, thermal accumulation effects, nonuniform cooling rates, and extrusion instabilities may induce warping phenomena, residual stresses and localised dimensional distortion, compromising geometric accuracy and reproducibility. In extrusion-based bioprinting systems, additional challenges emerge regarding bioink rheology, nozzle-induced shear stress, cell sedimentation and limited shape-retention capacity immediately after deposition.

Another important limitation involves the restricted surface quality commonly associated with extrusion-based systems. Layer stair-stepping effects and surface roughness may negatively influence cellular adhesion, biological integration, fatigue resistance and long-term functional performance of biomedical constructs. Moreover, the strong dependence of process stability on material viscosity, extrusion temperature, deposition speed and environmental conditions frequently complicates process standardisation and reproducibility across different manufacturing platforms.

From a translational perspective, ME also faces important barriers regarding sterilisation compatibility, process validation, long-term dimensional stability, and regulatory reproducibility for patient-specific medical devices. Consequently, although ME remains highly attractive for low-cost scaffold fabrication, customised prosthetic devices, and biofabrication applications, its future clinical scalability will strongly depend on advances in rheological control, multi-material integration, in situ monitoring, adaptive extrusion systems and high-precision process optimisation strategies.

***Critical Technological Limitations***—MJ

Despite its exceptional capability for producing high-resolution, multi-material and geometrically complex biomedical structures with excellent surface quality, MJ still exhibits several critical technological limitations that constrain its broader translational implementation in advanced biomaterial engineering. One of the principal challenges concerns the restricted range of printable biomaterials, as the process remains strongly dependent on low-viscosity photopolymeric formulations exhibiting highly specific rheological and curing characteristics. This limitation substantially reduces material diversity and frequently compromises the biological, mechanical and degradation properties required for functional biomedical applications.

The reliance on photopolymerisation mechanisms additionally introduces important concerns regarding residual monomers, incomplete curing, cytotoxicity and long-term material stability, particularly in implantable or cell-contact environments. Furthermore, photopolymeric materials commonly exhibit relatively limited mechanical robustness, brittleness, and susceptibility to aging or hydrolytic degradation, restricting their suitability for load-bearing biomedical structures or long-term physiological exposure.

Another critical limitation involves the sensitivity of droplet formation and deposition stability to environmental conditions, nozzle performance and fluid rheology. Minor fluctuations in viscosity, temperature, or jetting frequency may generate deposition irregularities, droplet misalignment, and dimensional inaccuracies capable of compromising reproducibility and structural fidelity. Nozzle clogging, satellite droplet formation, and support-material removal also remain important operational bottlenecks, particularly in highly detailed or internally complex architectures.

From a biomedical perspective, MJ additionally faces challenges regarding sterilisation compatibility, regulatory approval of photopolymeric materials, long-term biocompatibility validation, and scalable manufacturing reproducibility. Although technology offers remarkable advantages for anatomical modelling, surgical planning, and multi-material prototyping, its broader clinical translation toward functional implantable biomaterials will strongly depend on the development of advanced biocompatible resins, improved curing strategies, multimaterial integration approaches, and robust quality-assurance methodologies capable of ensuring long-term biological safety and structural.

The biological performance of extrusion- and jetting-based approaches is strongly governed by the interaction between feedstock rheology and process-induced stresses. High-viscosity bioinks may improve shape retention after deposition, but they can increase extrusion pressure and shear stress, thereby reducing cell viability or altering cell phenotype. Conversely, low-viscosity formulations are generally more favourable for cell survival but may lack the mechanical stability required to preserve scaffold geometry. Similar trade-offs apply to droplet-based jetting, where nozzle actuation, droplet formation and deposition dynamics can affect membrane integrity, cell distribution and post-print proliferation. Therefore, optimisation of these processes should not be limited to printability or dimensional fidelity; it must also consider cell viability, metabolic activity, immunomodulatory behaviour, matrix remodelling and the capacity of the construct to support tissue-specific maturation.

#### 3.1.4. Powder Bed Fusion (PBF)

PBF involves using thermal energy to fuse specific areas on a powder bed. Its advantages include high dimensional accuracy for creating small complex parts, the ability to fabricate without the need for supports and a wide range of printable powders. This technique is akin to dry powder BJ, with the key difference being the utilisation of thermal energy to bind the powder instead of a jetting binder. PBF stands out as one of the initial commercially adopted AM processes. Limón et al. [73] examined the challenge of translating LPBF-processed biodegradable Fe and Zn alloys into reliable implants, particularly regarding corrosion control, localised degradation, cytotoxicity, and long-term performance. The authors comprehensively reviewed that LPBF enables dense and porous Fe- and Zn-based implant architectures with promising mechanical performance, biodegradation tuning and biomedical applicability. Aufa et al. [74] highlighted persistent limitations of SLM-fabricated metallic implants, notably stress shielding, inadequate osseointegration, corrosion-related degradation and insufficient antibacterial performance in anatomical environments. The authors delivered a comprehensive review mapping current SLM trends, material-specific limitations, modification strategies, and future opportunities for improved biomedical implant performance. Wu et al. [75] addressed the challenge of processing biodegradable ZK60 by SLM while controlling pore formation, magnesium evaporation, dimensional accuracy, and excessively rapid corrosion. Optimised SLM produced fine-grained ZK60 with enhanced hardness, reduced hydrogen evolution and markedly improved corrosion resistance relative to cast alloy.

Within PBF, selective laser sintering (SLS) emerged as the first developed and commercialised PBF process. Another PBF process is selective laser melting (SLM) [28]. Sing et al. [76] reviewed the principal challenges in powder-bed AM of metallic implants, particularly controlled porosity, stress-shielding mitigation, dimensional accuracy, trapped powder, and restricted multi-material capability. It was found that SLM and EBM offer a highly promising route to patient-specific metallic implants, enabling complex geometries, tailored porosity, reduced lead times, and strong biomechanical potential. Behjat et al. [77] addressed the persistent challenge of developing load-bearing titanium implants with intrinsic antibacterial functionality while preserving corrosion resistance and cytocompatibility in AMed conditions. Findings highlight that EB-PBF-fabricated Ti536 achieved strong antibacterial performance through Ti_2_Cu precipitates and Cu-ion release without sacrificing biocompatibility or corrosion behaviour.

PBF has emerged as a key route for the fabrication of metallic implants, particularly where patient-specific geometries, controlled porosity, and high structural integrity are required. Its relevance is especially pronounced in orthopaedics and dentistry, where implant performance depends on the precise interplay between design, material response and biological integration.

Zhang et al. [78] studied the challenge of designing patient-specific femoral implants that improve anatomical fit, minimise bone resection and satisfy SLM manufacturability requirements. The authors accomplished a parametric CoCrMo SLM femoral implant with favourable bone conformity, interconnected porous truss architecture, high bearing capacity and limited powder adhesion. Gracheva et al. [79] addressed the challenge of translating SLM-processed biodegradable metals into clinically viable implants, particularly through degradation control, parameter optimisation, testing standardisation and regulatory alignment. It was demonstrated that SLM enables personalised biodegradable metallic implants with material-specific property tailoring, integrating controlled porosity, bone-matched stiffness and application-oriented performance across Mg, Fe and Zn systems. Rehman et al. [80] addressed the challenge of jointly optimising LPBF processing parameters to achieve high compressive strength, bone-comparable stiffness, low porosity and acceptable surface integrity in Ti6Al4V implants. Simulation-assisted multi-objective optimisation successfully identified optimal LPBF settings, delivering enhanced strength, near-cortical-bone modulus, limited porosity, and improved surface quality for load-bearing implants. Nadammal et al. [81] addressed the challenge of manufacturing a dense, low-modulus β-titanium alloy by L-PBF while minimising defects and preserving corrosion resistance and cytocompatibility. Near-full densification, low elastic modulus, high ductility, tensile strength of about 660 MPa and osteoblast response comparable to commercially pure titanium.

**Figure 7:**
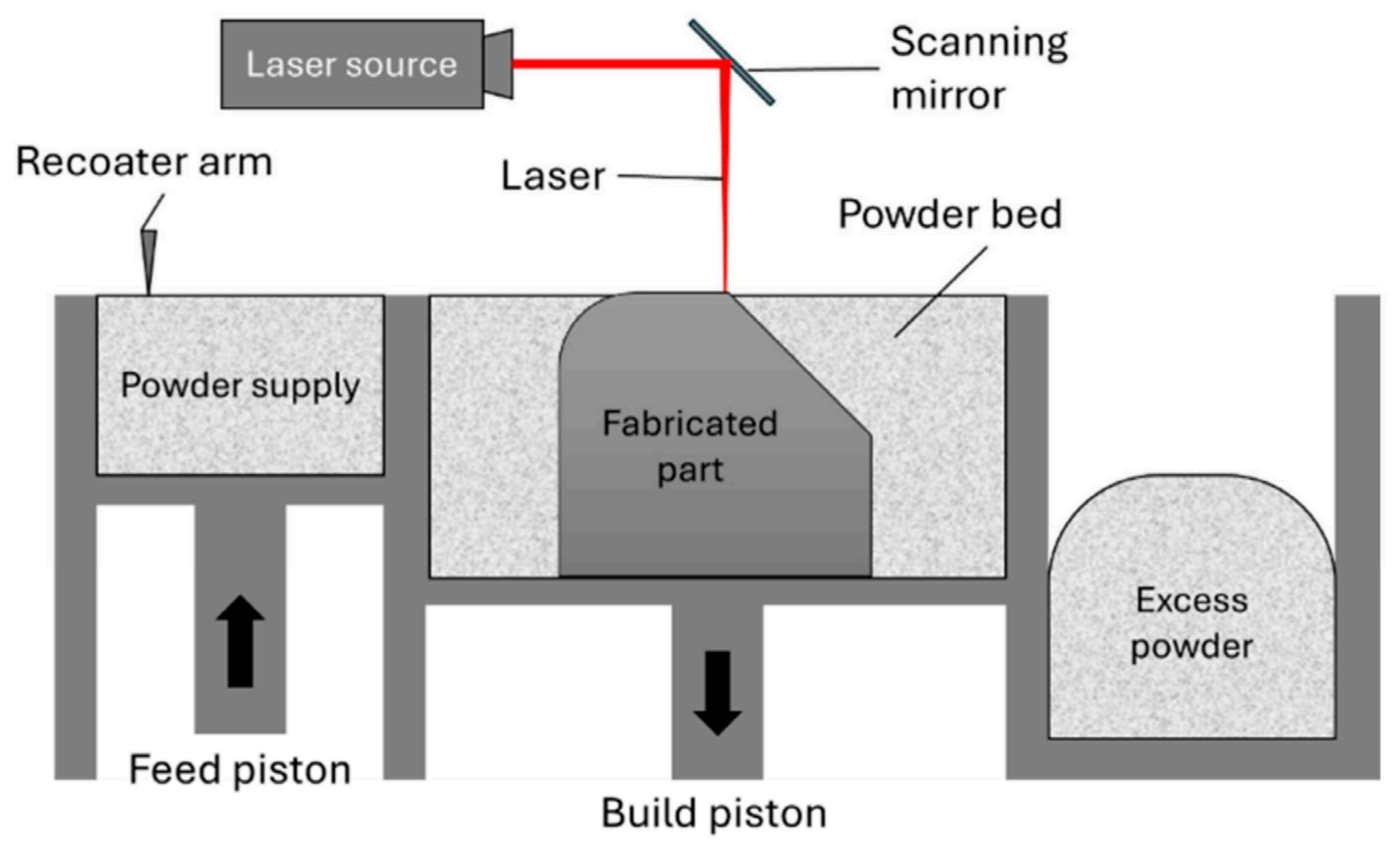
PBF process (Reprinted from [82]).

The PBF printing process follows similar steps to BJ. A schematic of the process is depicted in Figure 7. In brief, the energy source melts the powder after each layer is fed, leading to the construction of the 3D structure through layer-by-layer deposition. However, there are notable differences compared to BJ during the printing process. Prior to printing, a preheating process is essential until it reaches a temperature slightly below the glass transition or melting point of the powder. This preheating step reduces the power requirement of the energy source and facilitates powder fusion. Initial heating is also advantageous in decreasing the temperature gradient and minimising thermal distortion in the final part.

The governing parameters associated with this workflow include powder morphology, layer thickness, energy density, scan strategy, build orientation, thermal history and post-processing, which influence density, residual stress, surface roughness, fatigue behaviour and osseointegration potential.

Another significant distinction lies in the printing conditions. In contrast to the open setting used in BJ, an oxygen-free environment is essential for PBF to avoid oxidation of the feedstock powder. Various protective gas atmospheres, such as nitrogen for non-reactive powder, argon for reactive powder and vacuum for electron beam sources, are commonly employed in PBF [83,84] Depending on the thermal energy sources employed, PBF can be subcategorised into laser-based and electron beam-based PBF. Jamshidi et al. [85] examined the challenge of balancing mechanical reliability and biological response in SLMed Ti6Al4V, particularly across build anisotropy, residual porosity and surface-defect-sensitive post-processing routes. HIP markedly improved ductility, while chemical etching after HIP delivered the best fatigue performance and enhanced cellular affinity for bone implants. Valencia-Cadena et al. [86] addressed the challenge of reducing the high as-built roughness of EBM Ti6Al4V while preserving micromechanical integrity and ensuring reproducible surface-sensitive characterisation. It was exhibited that dry electropolishing markedly lowered surface roughness, stabilised hardness and reduced-modulus measurements, and improved surface uniformity without degrading the alloy’s intrinsic properties.

In laser PBF, there are two main techniques: selective laser sintering (SLS) and selective laser melting (SLM). These methods differ in their powder fusing mechanisms and material options. SLM involves the complete melting of the material to create a homogeneous part. It is a single-material process because different materials have varying melting points, although alloyed powders can still be utilised. On the other hand, in SLS, the laser heats the powder to a level where it can molecularly fuse at the surface [28].

Electron beam melting (EBM) employs an electron beam as the heat source to selectively meld powders. This method closely resembles Selective Laser Melting (SLM), with the key distinction that EBM utilizes a vacuum environment during the building process [87]. EBM is known for its swifter processing speed in comparison to SLM. The primary objective of employing a vacuum environment is to enhance the electron beam’s quality and mitigate the risk of powder or built material contamination caused by atmospheric gases [88].

Critical for PBF is the primary post-processing step of gradual cooling. Slowing down the cooling process to reach room temperature is essential to prevent issues like warping and cracking. While reducing the cooling rate is an effective strategy to minimize thermal distortion, it does extend the overall processing time. Additionally, various other post-processing techniques, such as polishing, machining, hot isostatic pressing, shot peening and heat treatment, are employed as needed based on the specific applications of the parts produced through AM (AM) [83].

Collectively, the reviewed PBF studies show that this route has the highest translational maturity for load-bearing metallic implants and patient-specific porous structures. Its value derives from the ability to fabricate dense or architecturally controlled metallic constructs with clinically relevant mechanical performance. However, the literature also shows that fatigue behaviour, residual stress, unmelted particles, surface roughness, corrosion response and osseointegration are strongly influenced by powder quality, energy density, build orientation, thermal history and post-processing. Thus, the biomedical performance of PBF components cannot be inferred only from density or strength; it requires integrated assessment of microstructure, surface state, fatigue resistance, ion release and biological response.

***Critical Technological Limitations***—PBF

Despite its exceptional capability for producing highly complex, high-density, and mechanically robust biomaterial structures with outstanding geometrical precision, PBF still presents several critical technological limitations that constrain its broader scalability, reproducibility, and translational reliability within advanced biomedical applications. One of the most significant challenges arises from the extreme thermal gradients and rapid solidification kinetics inherent to laser- or electron-beam-based fusion mechanisms, which frequently generate substantial residual stresses, anisotropic microstructures, thermal distortion and localised defect formation [89].

The repeated thermal cycling experienced during layer-by-layer fabrication additionally promotes heterogeneous grain evolution, microstructural instability, and nonuniform phase transformations, particularly in metallic biomaterials such as titanium alloys and cobalt–chromium systems. These phenomena may significantly influence fatigue resistance, corrosion behaviour, fracture toughness and long-term biomechanical reliability under physiological loading conditions. Furthermore, partially melted particles, lack-of-fusion defects, keyhole porosity, and powder contamination remain persistent challenges capable of compromising both structural integrity and biological performance.

Another major limitation concerns the strong dependence of process stability on powder characteristics, including particle morphology, size distribution, flowability, oxidation state and recycling history [90]. Variations in powder quality may directly affect energy absorption, melt-pool dynamics, layer uniformity, and dimensional reproducibility, complicating large-scale manufacturing consistency and quality assurance. Powder handling additionally introduces important occupational and environmental concerns related to particle inhalation, contamination control and reactive-metal safety management.

Surface quality also remains a critical issue in PBF-manufactured biomaterials. Although the process enables remarkable geometrical complexity, the resulting surface roughness and partially adhered particles frequently require extensive post-processing operations, including machining, polishing, heat treatment and surface functionalisation. In biomedical applications, these surface characteristics may substantially influence osseointegration behaviour, bacterial adhesion, wear resistance and cellular response.

From a translational perspective, PBF additionally faces important barriers regarding process standardisation, in situ monitoring, certification pathways, and regulatory reproducibility for patient-specific medical devices. The intrinsic complexity of melt-pool phenomena and thermal history evolution complicates predictive quality control and long-term validation, particularly for highly customised biomedical constructs. Consequently, although PBF remains one of the most promising AM technologies for advanced metallic implants and precision biomedical devices, its future clinical scalability will strongly depend on advances in real-time sensing, closed-loop process control, powder qualification methodologies, multiscale simulation and AI-assisted defect prediction strategies.

For metallic biomaterials, PBF provides a particularly strong example of process–structure–property coupling. Laser or electron-beam parameters determine melt-pool stability, porosity, surface roughness, residual stress and microstructure, all of which influence fatigue resistance, corrosion behaviour and biological integration. In porous orthopaedic implants, controlled porosity can reduce stiffness mismatch and promote bone ingrowth, but excessive or poorly connected porosity may compromise load-bearing reliability. Surface roughness can enhance cell attachment and osseointegration, yet it may also increase local corrosion susceptibility, ion release or bacterial adhesion if not properly controlled. Accordingly, post-processing operations such as heat treatment, hot isostatic pressing, machining, polishing or surface modification should be interpreted not as secondary finishing steps, but as determinants of both mechanical safety and biological response.

#### 3.1.5. VAT Photpolymerisation (VPP)

**Figure 8:**
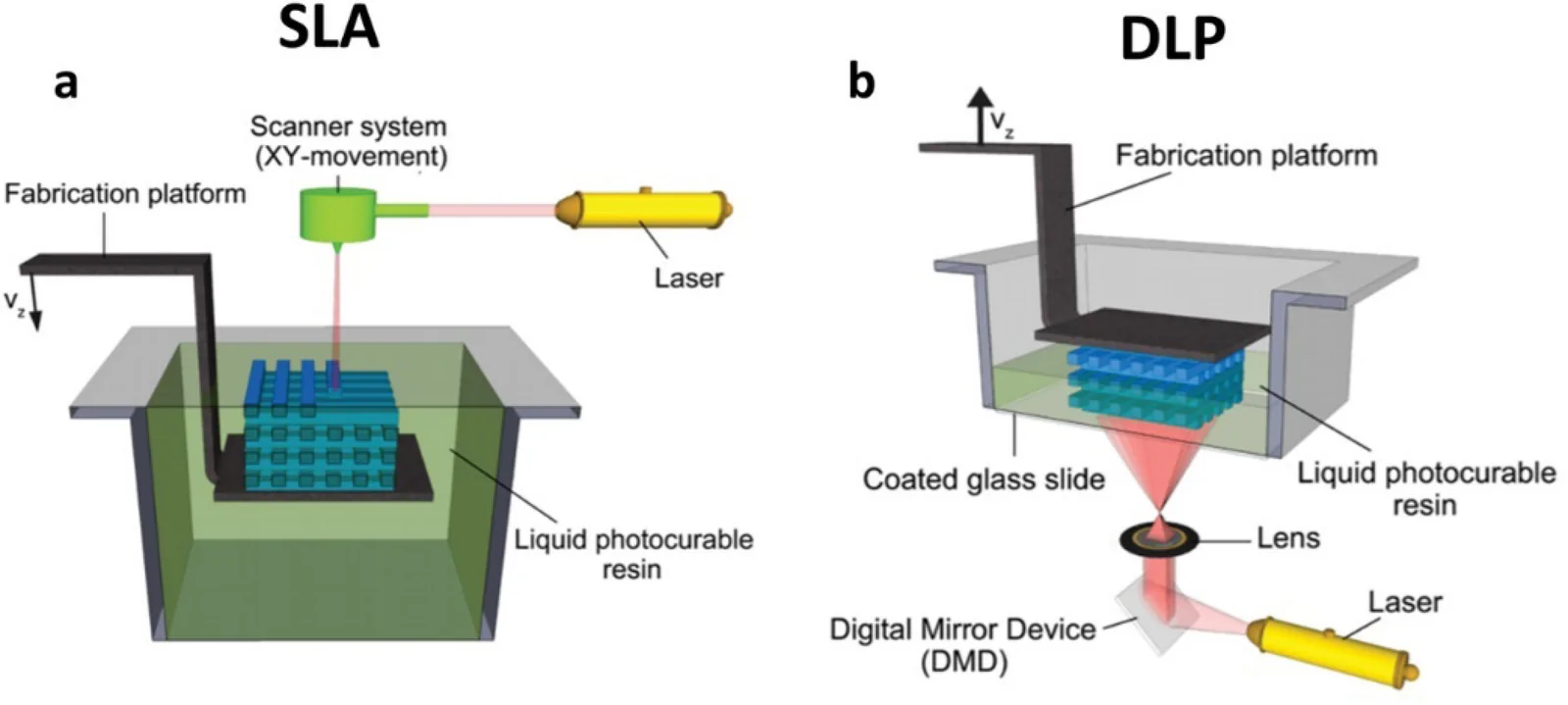
(**a**) Schematic showing the operation of a typical stereolithography (SLA) and (**b**) bottom-up Digital Light Processing (DLP) (Reprinted from [24]).

VPP, the initial AM method introduced for commercial use, manufactures components by selectively curing liquid photo-reactive polymers, often referred to as stereolithography (SLA, Figure 8). This technique is characterised by its high precision in building, rapid building speed, and outstanding part quality. Furthermore, it provides the flexibility to produce large-sized parts using various materials, including polymers, metals, and ceramics [28].

The overall process begins with formulating a solution in the vat, sometimes incorporating dispersants along with other essential components to ensure optimal part quality. Following the CAD file, the radiation pattern is reflected onto the solution surface to initiate the photopolymerisation of photo-reactive materials. After curing one layer, the platform descends for the photopolymerisation of the next layer until the entire process is completed. Two crucial factors, cure depth and cure width, must be controlled to achieve high-quality parts [91,92]. Three configurations of radiation sources are noteworthy for understanding differences in the photopolymerisation process.

- Vector scan: Like the early vat polymerisation process, a single laser beam acts as the radiation source, utilising optics and a scanning galvanometer for photopolymerisation into the vat. This method offers high resolution and excellent surface finish.
- Mask projection: Unlike the vector scan method, mask projection generates a large pattern using a digital micromirror device (DMD) and cures one layer at a time. The significant advantage is the high building speed.
- Two-photon VPP: In this method, two laser beams are involved, and curing occurs only at the intersections of the beams due to their high energy requirement. Unlike other methods, two-photon photopolymerisation does not require a build platform and can be accomplished within a solution. This approach enhances process resolution as photopolymerisation activates only at the centre of the beam intersection, where there is sufficient radiation energy [93].

Within the broader context of VPP, bioprinting has emerged as a particularly promising route for the fabrication of cell-laden constructs with high geometrical fidelity. Its relevance lies in the capacity to process photosensitive bioinks under comparatively mild conditions, enabling the production of architecturally complex scaffolds for advanced tissue-engineering applications. Chand et al. [94] addressed the challenge of fabricating a biomimetic corneal stroma equivalent that simultaneously achieves high print fidelity, native-like mechanics, optical transparency, and robust keratocyte viability. It was demonstrated that DLP-printed GelMA–OxiCMC interpenetrating-network hydrogels yielded reproducible corneal constructs with native-like curvature, matched compressive modulus, restored transparency, and >93% cell viability. Alparslan and Bayraktar [95] examined the persistent challenges of limited biomaterial diversity, scale-up constraints, and the optimisation of DLP bioprinting parameters for reliable biomedical fabrication. The authors provided a comprehensive review showing how smart biomaterials expand DLP bioprinting potential across tissue engineering, regenerative medicine, drug delivery, and biosensing. Mahdavi et al. [96] addressed the challenge of overcoming collagen’s limited mechanical strength and poor printability while preserving cytocompatibility in photocrosslinkable bioinks for corneal stromal applications. collagen methacrylate–poly(ethylene glycol) diacrylate (ColMA–PEGDA) bioinks supported human corneal stromal cell (hCSC) viability, geometry-dependent cell organisation and favourable corneal-marker expression, with 1-vinyl-2-pyrrolidinone (NVP) content effectively tuning biological performance.

The key process variables represented by this workflow include resin chemistry, light dose, exposure time, penetration depth, photoinitiator concentration, conversion degree, washing and post-curing, which determine resolution, residual monomer content, cytocompatibility and degradation behaviour.

Furthermore, Afridi et al. [97] reviewed the challenge of advancing stereolithography across biomedical, mechanical and electrical domains while overcoming material constraints, curing complexity, shrinkage, and process-specific limitations. Additionally, they demonstrated that stereolithography offers exceptional resolution, surface quality, precision and application versatility, with continuous developments in materials, systems and multifunctional manufacturing capabilities. Guerra Silva et al. [98] addressed the challenge of fabricating miniature lattice structures while reconciling the lower geometric fidelity of ME with the material limitations of VPP. VPP achieved superior geometric accuracy, whereas ME delivered higher stiffness and energy absorption, yielding broadly comparable compressive performance overall.

There are two primary types of polymerisations in VPP: free-radical and cationic polymerisation. In free-radical polymerisation, photo-reactive polymers initially form long chains and then come close to each other, followed by a crosslinking reaction. Acrylates are the most common photo-reactive materials used in free-radical polymerisation, offering high reaction speed. However, challenges include high shrinkage and mechanical issues like curling and warping. Cationic polymerisation typically begins with a polymer having a ring structure, such as epoxies. In this process, the rings open, creating active positions for the formation of other chemical bonds. Because the number and structure of the new bonds closely resemble the original chemical bond, there is minimal curling, warping, and shrinkage. Combining these two polymerisation methods is sometimes done to achieve faster builds with excellent part quality. In addition to photo-reactive materials, photoinitiators play a crucial role in reducing the need for thermal energy in polymerisation, functioning similarly to catalysts in chemical reactions [99]. Xu et al. [100] addressed the difficulty of fabricating hydrogel scaffolds with simultaneously high DLP print fidelity and a broadly tunable modulus, without repeated reformulation or compromised manufacturability. A two-step DLP–ion-crosslinking strategy enabling 10 μm-resolution scaffolds with adjustable modulus across 15.8–345 kPa, successfully guiding cardiac and vascular tissue organisation. Xu et al. [101] investigated the challenge of predicting cell viability in dynamic optical projection stereolithography, where UV exposure, hydrogel composition and layer geometry interact nonlinearly. The authors developed an ensemble machine-learning model that predicted post-printing cell viability with high accuracy and identified UV exposure time as the dominant process variable. Liang et al. [102] addressed the challenge of establishing a rigorous benchmark for vat-photopolymerised hydroxyapatite, reconciling printability, dimensional fidelity, mechanical reliability and biological performance. VPP-processed hydroxyapatite achieved near-full density, sub-1% dimensional error, exceptional bending strength, and favourable osteogenic activity for bone-repair applications.

Collectively, recent studies published in 2024–2025 illustrate a clear transition from process feasibility towards biomaterials-specific validation, particularly in DLP bioprinting, photoreactive collagen-based bioinks, hydrogel scaffolds with tunable modulus and vat-photopolymerised hydroxyapatite systems. These studies reinforce the need to evaluate light-based AM not only in terms of resolution and shape fidelity, but also through cytocompatibility, conversion degree, degradation products, mechanical adaptability and tissue-specific biological response.

Vat polymerisation cytotoxicity primarily arises from residual monomers, photoinitiators, photoabsorbers and incomplete curing, which may impair cell adhesion, viability, proliferation, and long-term functionality after fabrication. Männel et al. [103] addressed the challenge of developing µSL-compatible resins that avoid cytotoxic leaching while retaining suitable wettability, swelling behaviour, surface properties and HUVEC compatibility. It was identified PEGDA/PEGMEMA-based formulations and post-processing conditions that yielded non-cytotoxic 3D-printed substrates capable of supporting HUVEC viability and proliferation for cell-culture applications. Brooks and Yadavalli [104] addressed the challenge of reducing cytotoxic leachates from light-cured photopolymer resins without compromising optical clarity, practicality, or accessibility for biomedical use. A short post-cure combined with double IPA washing markedly reduced cytotoxicity, preserving near-complete cell viability in clear resins. Jeršovaitė et al. [105] addressed the challenge of reducing residual-monomer toxicity in optically printed microporous scaffolds while clarifying how post-processing and degree of conversion govern biocompatibility. UV–solvent post-processing, including Soxhlet extraction, enhanced scaffold biocompatibility, supported DPSC adhesion and osteogenic differentiation, and linked viability with conversion.

The evidence reviewed for vat photopolymerisation indicates that this route is particularly valuable when high resolution, architectural definition and controlled scaffold geometry are required. However, its biomedical applicability is limited by the chemistry of the photopolymer system. Residual monomers, photoinitiator toxicity, light exposure, conversion degree, washing, post-curing and degradation products may all influence cytocompatibility and long-term biological safety. Therefore, recent progress in vat-based biomaterial AM should be interpreted as a transition from geometrical feasibility towards biomaterials-specific validation, where print resolution must be balanced against cytocompatibility, mechanical adaptability and tissue-specific function.

***Critical Technological Limitations—VPP***

Despite its outstanding capability for fabricating highly detailed and geometrically complex biomaterial constructs with exceptional surface quality and dimensional fidelity, VPP still exhibits several critical technological limitations that constrain its broader translational applicability in advanced biomedical engineering. One of the most significant challenges concerns the strong dependence of the process on photopolymeric materials exhibiting highly specific optical, rheological and curing characteristics. This restricted material compatibility substantially limits the diversity of biomaterials available for clinically relevant applications, particularly when simultaneous mechanical robustness, biodegradability and biological functionality are required.

The photopolymerisation process itself additionally introduces important concerns regarding incomplete curing, residual monomer retention and photoinitiator-related cytotoxicity. These factors may negatively affect long-term biocompatibility, cellular response, inflammatory behaviour and degradation stability, especially in implantable biomedical environments. Furthermore, many photocurable materials still exhibit relatively brittle mechanical behaviour, limited fatigue resistance and susceptibility to hydrolytic or ultraviolet-induced degradation, restricting their applicability in load-bearing or long-term physiological conditions.

Another major limitation involves the high sensitivity of the process to optical scattering, light penetration depth, resin viscosity and exposure parameters. Minor variations in curing conditions may significantly influence polymerisation kinetics, dimensional accuracy, internal defect formation and interlayer bonding quality. In ceramic-loaded or particle-reinforced suspensions, sedimentation phenomena, light attenuation and heterogeneous curing further complicate process stability and reproducibility. Additionally, support-structure generation and removal frequently remain challenging for highly intricate geometries, potentially affecting surface integrity and dimensional precision.

From a translational perspective, VPP also faces substantial challenges regarding sterilisation compatibility, process validation, long-term material characterisation and regulatory approval of photocurable biomaterials intended for direct clinical use. The limited availability of clinically certified photopolymers and the complexity associated with ensuring reproducible curing behaviour across different manufacturing platforms continue to hinder large-scale biomedical implementation. Consequently, although VPP remains one of the most promising technologies for high-resolution biomedical fabrication, microarchitectured scaffolds and patient-specific medical devices, its future clinical scalability will strongly depend on advances in biocompatible resin development, multimaterial photopolymerisation, real-time curing control and integrated quality-assurance methodologies capable of ensuring both structural reliability and long-term biological safety.

In vat photopolymerisation, the principal biological concern arises from the chemistry of the photo-crosslinking system. Although these processes offer high resolution and excellent architectural control, residual monomers, photoinitiators, degradation products and incomplete post-curing can induce cytotoxic effects and alter cell adhesion or proliferation. Light exposure may also affect encapsulated cells or bioactive molecules, depending on wavelength, intensity and exposure time. Consequently, the suitability of vat-polymerised biomaterials cannot be judged by resolution alone. It requires evaluation of conversion degree, leachable residues, sterilisation compatibility, degradation products, cell response and inflammatory potential, particularly when the printed construct is intended for direct contact with living tissue.

Recent work published in 2024–2025 further illustrates this transition from process feasibility towards biomaterials-specific validation, particularly in DLP bioprinting, photoreactive collagen-based bioinks, hydrogel scaffolds with tunable modulus and vat-photopolymerised hydroxyapatite systems. These studies reinforce the need to evaluate light-based AM not only in terms of resolution and shape fidelity, but also through cytocompatibility, conversion degree, degradation products, mechanical adaptability and tissue-specific biological response.

### 3.2. Cellular Techniques—Bioprinting

The same AM process families discussed in the acellular context may also inform biofabrication; however, in 3D bioprinting their evaluation is governed primarily by cell viability, bioink rheology, crosslinking kinetics, nutrient transport, tissue maturation and biological function. Therefore, this section does not repeat the generic operating principles of extrusion, jetting or light-based AM, but focuses on the additional constraints introduced by cell-containing and biologically active formulations.

**Figure 9:**
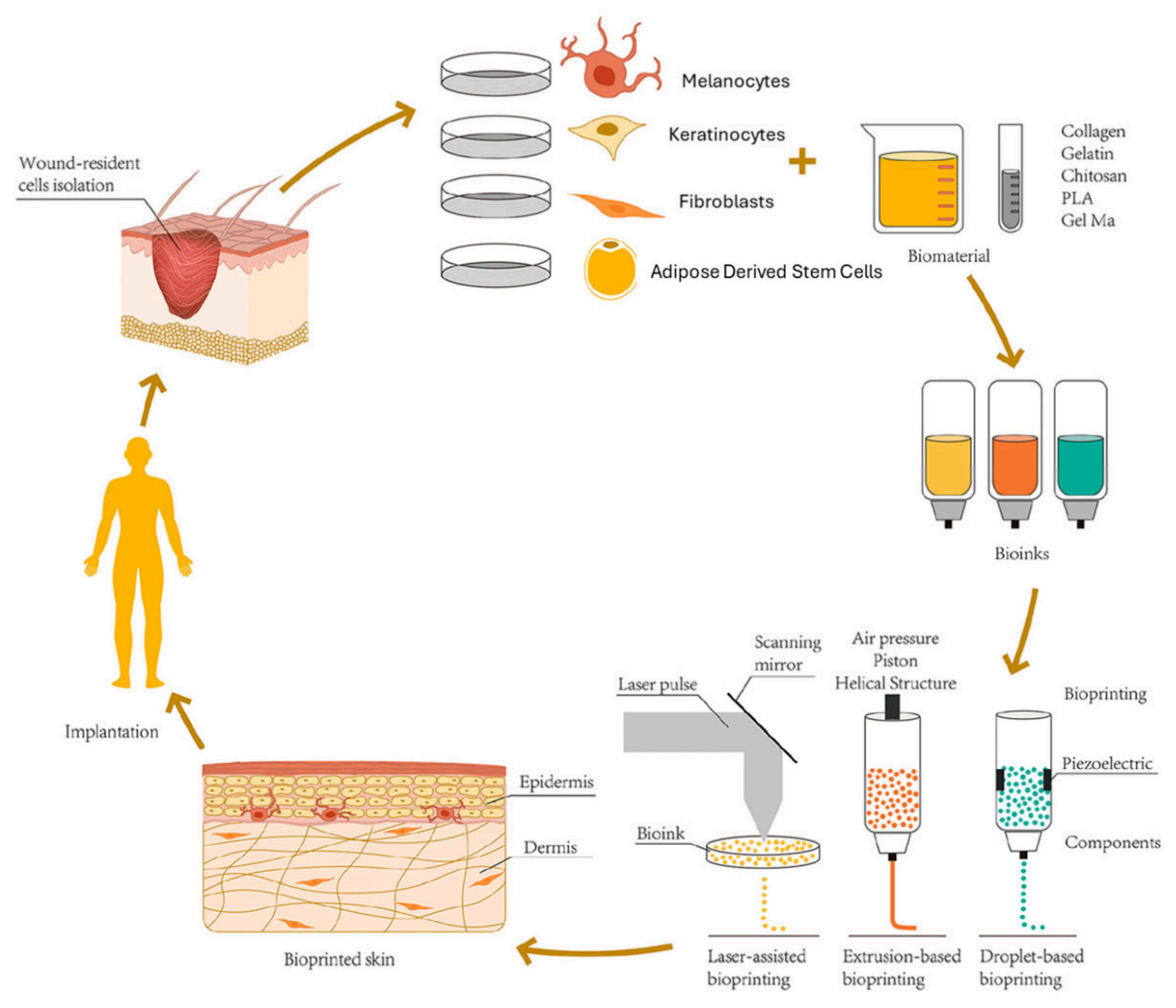
Schematic representation of skin 3D bioprinting (Reprinted from [114]).

3D bioprinting, which combines 3D printing with biology, represents an advanced technique in AM and the deposition of biomaterials layer by layer onto substrates when placed in compatible biomaterials (Figure 9) [106]. In simpler terms, the process involves the printing and patterning of cells on a substrate or tissue using an automated dispensing system [107,108]. The choice of biomaterials in 3D bioprinting is closely tied to the intended application of the final product, and there is a diversity in the technology employed for 3D bioprinting.

Material–process compatibility is particularly critical in hydrogel-based bioprinting because printability and biological performance are governed by competing requirements. Hydrogels intended for extrusion bioprinting must possess sufficient viscosity and shear-thinning behaviour to maintain filament continuity and shape fidelity after deposition, while avoiding excessive extrusion pressure that may damage encapsulated cells. Formulations with inadequate viscosity tend to collapse, spread or lose pore definition, whereas overly viscous systems can increase shear stress, reduce cell viability and impair homogeneous cell distribution. Therefore, the relevant processing window is not defined by viscosity alone, but by the combined balance between rheology, nozzle diameter, extrusion pressure, printing speed, crosslinking kinetics and cell density.

Crosslinking strategy further determines the stability and biological suitability of printed hydrogel constructs. Ionic crosslinking provides rapid gelation and is useful for maintaining early shape fidelity, but may offer limited long-term mechanical stability. Photocrosslinking enables improved spatial control and structural reinforcement, although photoinitiator concentration, light dose and exposure time must be controlled to avoid cytotoxic effects. Thermal gelation and enzymatic crosslinking can improve biological compatibility, but may be slower or more sensitive to processing conditions. In many biofabrication systems, dual or sequential crosslinking approaches are therefore advantageous because they combine immediate post-deposition stability with subsequent mechanical reinforcement, improving printability without completely sacrificing cell viability or tissue-specific maturation.

Madem et al. [109] examined the persistent challenge of jointly optimising bioink printability, scaffold mechanical integrity, and cell viability across diverse bioprinting platforms and applications. A comprehensive simulation-informed review was given showing that integrating CFD and FEA can refine printing parameters, improve scaffold performance, and enhance cellular outcomes. Manzoli et al. [110] addressed the persistent challenge of preserving cell viability during and after 3D bioprinting, particularly under extrusion-induced shear stress and nutrient-limited construct conditions. A focused review was provided linking damage assessment, computational fluid-dynamics-based analysis, bioink optimisation, and micro-encapsulation strategies to improved cellular protection in bioprinting. Bożek et al. [111] tackled the challenge of adapting inkjet bioprinting to living-cell processing while minimising shear-induced damage, extending printable bioink options, and preserving post-printing viability. Pneumatic conveying inkjet bioprinting achieved approximately 93% cell viability, low impact velocity and gentle fibrin-based droplet deposition for bioprinting.

Rothbauer et al. [112] examined the difficulty of integrating bioprinting with organ-on-a-chip systems, particularly regarding bioink limitations, sealing compatibility, cell viability, resolution, and system cost. The authors praised how AM and 3D bioprinting enhance organ-on-a-chip architectures, enabling improved spatial control, vascularisation, biomimicry and microphysiological functionality. Khan et al. [113] addressed the persistent difficulty of bioprinting complex soft-matter constructs, where highly compliant bioinks compromise shape fidelity and conventional post-curing may threaten cell viability. An SLA-fabricated elastic mould combined with extrusion bioprinting markedly improved geometric fidelity, enabled human ear fabrication and preserved high cellular viability.

Various bioprinting approaches exist, like the ones explained above, each rooted in distinct fundamental principles for constructing functional tissue structures. These include inkjet-based bioprinting, laser-assisted bioprinting (LAB), pressure-assisted (extrusion) bioprinting, acoustic bioprinting, stereolithography (SLA)-based bioprinting and magnetic bioprinting. These strategies can be employed individually or in combination to attain specific goals in AM and tissue fabrication [107].

For bioprinting purposes, biomaterials or polymers are available in the form of gels, powders and/or particles, and they need to be prepared in a solution by using water or other solvents. Polymers fall into two categories: water-soluble and non-water soluble. Water-soluble materials can be directly dissolved in water or water-based solutions. Biomaterials commonly used for printing, such as alginate and gelatin, belong to the water-soluble category and may take minutes to hours to dissolve. While many materials exhibit slight solubility in water under neutral pH and room temperature conditions, they can be easily dissolved at non-neutral pH or elevated temperatures [115]. Non-water-soluble biomaterials used in extrusion-based bioprinting for scaffold fabrication need to be transformed into solution-like phases before application. To achieve this, these non-soluble materials are either dissolved in special organic solvents or thermally melted by carefully controlling the temperature during ME.

To create structures that emulate native tissues or organs, it may be necessary to formulate biomaterial solutions containing living cells (referred to as bioinks) for the process of bioprinting. Kanokova et al. [116] investigated the challenge of sustaining viable cell culture within highly concentrated collagen bioinks, where static cultivation restricts nutrient transport, metabolite removal and effective remodelling. It was demonstrated that active medium perfusion markedly enhanced cell viability, metabolic activity and substrate remodelling, thereby strengthening the translational potential of collagen bioprinting. Gharacheh and Guvendiren [117] addressed the challenge of formulating a cell-laden composite bioink that reconciles printability, photocrosslinkable stability and robust osteogenic support in bone-mimetic scaffolds. Human bone allograft particles enhanced rheology, stiffness and hMSC osteogenic differentiation while maintaining high long-term viability in bioprinted scaffolds. Chen et al. [118] examined the challenge of reconciling hydrogel printability, structural stability and post-printing cell survival, while identifying concentration–needle combinations suitable for scaffold fabrication. GRGD-modified Pluronic F127 enabled stable three-dimensional constructs and supported fibroblast viability and proliferation, indicating strong promise as a bioink.

Consequently, the prepared solutions must establish an aqueous environment conducive to cell survival and suitable for the printing process [119]. Currently, polymers, gelled polymers or hydrogels are the most utilised biomaterials for cell bioprinting. Ku et al. [120] focused on the challenge of developing thermosensitive chitosan bioinks that combine favourable gelation behaviour, low cytotoxicity, nozzle-processability and sustained cell viability for bioprinting. It was demonstrated that NaHCO_3_-gelled chitosan bioinks, particularly in acetic acid, offered superior printability, reduced clogging tendency and maintained high viability in PDLSC-laden constructs. Khatun et al. [121] addressed the challenge of developing a bioresponsive, moderately stable hydrogel that simultaneously supports high cell-density bioprinting, controlled degradation, and robust post-printing fidelity. It was found that genipin-crosslinked gelatin–hyaluronic acid hydrogel enabled high-fidelity complex printing, excellent cytocompatibility and enhanced cell–cell interaction following scaffold disintegration.

These materials offer a soft environment that ensures the viability of cells within the solutions, and the resulting hydrogel is an aqueous solution. The hydrogel, formed after solidification, contains a significant water content, and exhibits properties akin to natural tissues. Natural hydrogels are preferred due to their inherent biocompatibility, while synthetic hydrogels provide more consistent and predictable properties. Commonly employed hydrogels include alginate, chitosan, agarose, hyaluronic acid (HA), collagen, gelatin, fibrin, polyethylene glycol (PEG), and polyethylene oxide (PEO). Decellularised extracellular matrices (dECM) have also been developed as biomaterials for bioprinting scaffolds along with cells [122]. Particularly, a significant obstacle in constructing structures from natural hydrogels lies in the limitation of their mechanical properties [123]. To address this challenge, extrusion bioprinting often incorporates non-water-soluble polymers with robust mechanical strength in conjunction with hydrogels to create hybrid structures. These polymers are typically dissolved in a solvent or melted within the bioprinter by carefully controlling the temperature during the printing process. Subsequently, they are extruded in a layer-by-layer fashion to generate scaffold structures. However, the use of solvent or the elevated temperature environment can pose serious issues concerning the survival of cells and tissues, preventing their addition or integration into these materials [122].

Across the bioprinting literature, the main challenge is the simultaneous optimisation of printability, cell viability and tissue-specific functionality. The reviewed studies collectively show that bioink performance cannot be reduced to a single property such as viscosity or gelation rate. Instead, successful bioprinting depends on the coordinated control of rheology, shear-thinning behaviour, nozzle geometry, extrusion pressure, crosslinking kinetics, cell density, nutrient diffusion, matrix remodelling and post-print maturation. This explains why many promising laboratory-scale bioinks remain difficult to translate: constructs that are printable may not provide sufficient biological function, whereas highly cell-compatible systems may lack the mechanical stability required for reproducible fabrication and handling.

***Critical Technological Limitations—Bioprinting***

Despite its revolutionary potential for fabricating biologically functional, cell-laden and patient-specific tissue constructs, bioprinting still faces several critical technological limitations that substantially constrain its large-scale translational implementation in regenerative medicine and advanced biomedical engineering. One of the most fundamental challenges concerns the simultaneous optimisation of printability, biological viability and structural integrity, as these requirements frequently exhibit conflicting rheological and biological demands. Bioinks with sufficient viscosity to preserve geometrical fidelity often generate elevated shear stress during extrusion, compromising cell viability, proliferation and differentiation potential.

The intrinsic fragility and low mechanical strength of most hydrogel-based bioinks additionally limit the fabrication of mechanically stable three-dimensional structures, particularly for load-bearing tissues such as bone, cartilage or musculoskeletal interfaces. Furthermore, maintaining homogeneous cell distribution, controlled nutrient diffusion and long-term cellular functionality throughout complex volumetric constructs remains a major challenge, especially in larger tissue architectures where oxygen and nutrient transport become diffusion-limited.

One of the most critical translational bottlenecks in bioprinting concerns vascularisation. Although substantial progress has been achieved in microchannel fabrication and angiogenic biofunctionalisation, the generation of fully functional vascular networks capable of supporting clinically relevant tissue thicknesses remains largely unresolved. Insufficient vascular integration frequently results in necrotic core formation, poor tissue maturation and limited long-term viability after implantation. Additionally, the biological complexity associated with reproducing native extracellular matrix organisation, multicellular interactions, and tissue-specific microenvironments further complicates the development of fully functional engineered tissues.

Another important limitation involves the extreme sensitivity of bioprinting processes to environmental conditions, sterility control, bioink rheology, crosslinking kinetics and process parameters. Minor fluctuations in temperature, pH, nozzle geometry, printing speed or crosslinking conditions may significantly affect deposition stability, cell survival, structural fidelity and biological reproducibility. Sterile manufacturing environments, contamination prevention and long-term storage stability also remain major operational and logistical challenges for clinical-scale implementation.

From a regulatory and translational perspective, bioprinting faces substantial barriers regarding process standardisation, quality assurance, ethical governance, reproducibility and long-term in vivo validation. The intrinsic biological variability associated with living-cell systems complicates process certification and regulatory approval pathways, particularly for patient-specific therapies involving autologous cells or personalised tissue constructs. Consequently, although bioprinting represents one of the most transformative technologies in modern regenerative medicine, its future clinical scalability will strongly depend on advances in vascularisation strategies, smart bioinks, multi-material integration, real-time bioprocess monitoring, AI-assisted process optimisation and robust translational frameworks capable of ensuring biological reliability, manufacturing consistency and long-term therapeutic safety.

**Figure 10:**
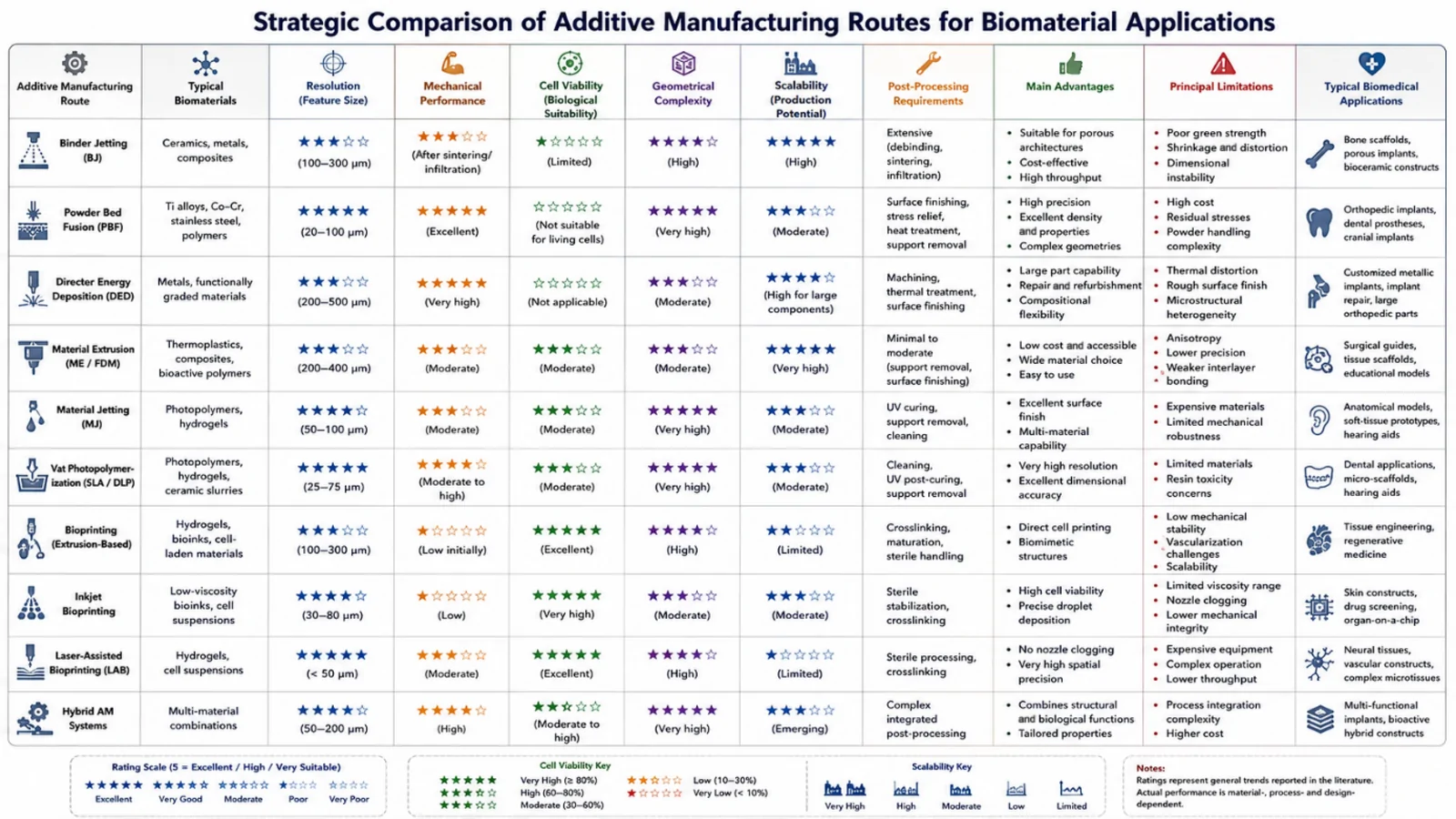
Strategic comparison of AM routes for biomaterial applications, considering biomaterial compatibility, geometrical resolution, mechanical performance, biological suitability, scalability, post-processing requirements, technological advantages, operational limitations and representative biomedical applications associated with the principal AM technologies (figure done with Microsoft^®^ Excel and Microsoft^®^ Visio Pro 2021).

Given the wide diversity of AM technologies currently explored for biomaterial fabrication, a systematic comparative assessment becomes essential for understanding the multidimensional trade-offs governing process selection and translational applicability. Different AM routes exhibit markedly distinct capabilities in terms of geometrical resolution, biological compatibility, mechanical integrity, scalability and post-processing requirements, directly influencing their suitability for specific biomedical applications. The strategic comparison presented in Figure 10 synthesizes the principal technological characteristics, operational advantages, performance limitations and biomedical applicability associated with the main AM approaches currently employed in biomaterials engineering. This comparative perspective provides a more integrated understanding of the complex relationships existing between biomaterial selection, manufacturing strategy, structural performance and clinical translation potential.

The comparative analysis of AM technologies for biomaterial applications clearly demonstrates that process selection cannot rely exclusively on isolated criteria such as geometrical resolution, manufacturing speed or material compatibility alone. In practice, the successful implementation of AM within biomedical environments requires the simultaneous consideration of multiple interdependent factors involving biological performance, mechanical integrity, process reproducibility, regulatory readiness, scalability, post-processing complexity and long-term clinical applicability. Furthermore, different biomedical applications frequently impose conflicting requirements regarding porosity, structural strength, biodegradation behaviour, patient specificity and translational feasibility, making technology selection a highly multidimensional decision-making process. Consequently, the increasing complexity of biomaterial engineering is progressively driving the development of integrated decision-support methodologies capable of systematically connecting clinical requirements, biomaterial characteristics, manufacturing constraints and translational objectives.

AM technology selection should not be interpreted as a purely technical or material-driven decision, but rather as a multidimensional optimisation problem involving continuous interaction between engineering performance, biological functionality, manufacturing maturity, regulatory feasibility and clinical objectives. Importantly, the figure also demonstrates that the optimal manufacturing strategy frequently depends on balancing competing priorities, including precision, scalability, cost-efficiency, structural reliability, patient specificity and translational readiness. However, although decision-support frameworks can significantly improve process selection and technological alignment, successful clinical implementation ultimately requires a broader translational infrastructure capable of integrating digital design, biomaterial development, manufacturing ecosystems, quality assurance, regulatory governance and long-term clinical validation. Within this context, the following section introduces the concept of an integrated translational ecosystem for AM of biomaterials, emphasising the dynamic interactions required to transform laboratory-scale innovations into clinically reliable and commercially sustainable biomedical solutions.

The comparative analysis previously presented for the principal AM routes highlighted that no single technology currently satisfies all the mechanical, biological, manufacturing, regulatory and translational requirements associated with advanced biomaterial applications. Instead, each manufacturing strategy exhibits specific advantages, limitations and performance trade-offs directly influenced by process parameters, material compatibility, structural complexity, scalability, post-processing requirements and clinical applicability. As AM technologies progressively evolve from laboratory-scale experimentation toward clinically oriented biomedical ecosystems, the successful development of next-generation biomaterial solutions increasingly depends on the integration of digital design, biomaterial engineering, process optimisation, translational validation, regulatory alignment and continuous clinical feedback within unified innovation frameworks. Consequently, AM can no longer be interpreted as an isolated fabrication stage, but rather as a highly interconnected translational ecosystem linking materials science, computational engineering, manufacturing technologies, quality assurance, regulatory science and patient-centred healthcare solutions.

Future advances in AM of biomaterials will depend not only on incremental improvements in fabrication technologies, but also on the ability to establish integrated and adaptive translational infrastructures capable of connecting scientific innovation, industrial scalability, regulatory robustness and clinical reliability. Importantly, the figure highlights the continuous feedback mechanisms existing between design optimisation, material characterisation, manufacturing performance, biological validation, post-market surveillance and real-world clinical outcomes. Such interdependencies reinforce the necessity for multidisciplinary collaboration involving engineers, clinicians, material scientists, manufacturers, regulatory agencies and data scientists throughout the entire translational lifecycle. Within this context, the following discussion critically examines the major scientific, technological, organisational and translational challenges that continue to limit the large-scale implementation of AM technologies in biomedical applications, while simultaneously identifying the emerging paradigms expected to shape the future evolution of this rapidly expanding field.

## 4. Discussion

The reviewed literature indicates that the central challenge in AM of biomaterials is no longer the mere demonstration that complex constructs can be fabricated. Rather, the critical issue is the translation of printable biomaterial systems into reproducible, biologically functional and clinically meaningful constructs. Across the different additive-manufacturing routes considered in this review, recurring tensions are evident between geometrical resolution and cell viability, mechanical reinforcement and biodegradability, architectural complexity and manufacturability, post-processing and biological preservation and experimental innovation and regulatory standardisation. These tensions explain why many published studies remain at the level of process feasibility or proof-of-concept demonstration, while fewer provide robust evidence of long-term biological performance, scalable manufacturing control or clinically relevant validation. The discussion therefore focuses on the cross-route relationships and unresolved knowledge gaps that connect the reviewed studies, rather than treating BJ, DED, ME, MJ, PBF, vat photopolymerisation and bioprinting as independent technological categories.

A further implication of the reviewed literature is that process limitations should be interpreted through their biological consequences. Low resolution, residual porosity, anisotropy and surface roughness are not merely manufacturing constraints; in biomedical constructs, they may alter cell attachment, nutrient diffusion, degradation rate, vascularisation potential, mechanical load transfer and host response. Similarly, post-processing cannot be assessed only by its ability to improve strength or dimensional stability, because thermal exposure, chemical infiltration, solvent removal, photopolymerisation and surface modification may also affect cytocompatibility, immunogenicity and degradation behaviour. This is particularly relevant for degradable polymers, calcium-phosphate ceramics, magnesium-based alloys, hydrogels and cell-laden bioinks, where biological performance depends on the interaction between material chemistry, architecture and the local physiological environment. Therefore, a critical comparison of AM routes for biomaterials must integrate quantitative manufacturing indicators with biological endpoints, including cell viability, proliferation, inflammatory response, degradation kinetics and tissue-specific functional maturation.

### 4.1. Cross-Technology Comparison and Translational Implications

To address the comparative implications of the reviewed literature more explicitly, the principal AM routes considered in this review were reassessed according to their relative technological strengths, material compatibility, biological constraints and translational potential. This comparison is important because the suitability of a given AM route for biomaterial fabrication cannot be determined from process capability alone. Instead, technology selection must consider the target tissue, required mechanical function, acceptable post-processing burden, intended biological response, scalability and likely regulatory pathway. Table 4 therefore summarises the principal material–process limitations and trade-offs associated with the AM routes considered in this review, linking them to their expected biological and translational consequences.

This comparison shows that AM-route selection cannot be based only on resolution, speed or material compatibility. For biomaterials, each processing route must also be assessed according to its effects on biological response, post-processing burden, reproducibility, scale-up and clinical readiness.

At the same time, the review highlights a marked contrast between the relative maturity of acellular manufacturing and the still-developing status of cellular and biofabrication-based approaches. Hydrogel systems and cell-laden bioinks expand the functional scope of AM far beyond the fabrication of inert scaffolds, enabling the production of constructs more closely aligned with the biological complexity of soft tissues. However, this transition from structural manufacture to biologically active fabrication introduces a different class of constraints. In cellular systems, printability can no longer be judged solely by dimensional accuracy or geometric resolution; it must also account for rheological behaviour, shear exposure, nutrient transport, cytocompatibility, curing-induced toxicity and post-printing cell function. Consequently, the field is increasingly defined by the need to reconcile competing requirements that are often difficult to satisfy simultaneously: structural fidelity, mechanical sufficiency, process stability and biological viability.

Beyond these process-level effects, the biological relevance of AMed biomaterials depends on how manufacturing decisions influence cellular, molecular and immunological responses. Surface chemistry, pore interconnectivity, stiffness gradients, degradation products, residual processing agents and post-processing history may regulate protein adsorption, cell adhesion, macrophage polarisation, osteogenic or chondrogenic differentiation, extracellular-matrix deposition and local inflammatory signalling. For metallic systems, ion release, corrosion behaviour and surface oxide stability are central to long-term tissue compatibility. For degradable polymers and hydrogels, degradation kinetics, swelling behaviour, leachable residues and by-product acidity may affect both cytocompatibility and immune response. For cell-laden systems, shear exposure, crosslinking chemistry, oxygen and nutrient transport and post-print maturation conditions directly influence viability, phenotype maintenance and tissue-specific function. Therefore, biological performance should be treated as an intrinsic design variable within AM of biomaterials, rather than as a validation step performed only after fabrication.

A further point of significance concerns the role of post-processing, which the literature consistently shows to be far more than a secondary finishing stage. In many biomaterial systems, especially metallic and binder-jetted constructs, post-processing decisively governs final performance through its influence on porosity, dimensional stability, residual stress, surface state, corrosion response, interfacial behaviour and biological interaction. In this regard, recent work already cited in the manuscript reinforces the argument advanced throughout this review. Zhu et al. [124] for instance, show that post-processing in binder-jetted materials must be understood as a geometrically and microstructurally transformative stage rather than a merely corrective one, while Kumar et al. [125] emphasise that surface modification, hybrid bioactive coatings and post-processing strategies remain indispensable for improving the clinical suitability of biomedical implants. These observations support the broader conclusion that AM should be treated as an integrated process chain in which fabrication and post-fabrication are inseparable from one another.

The discussion also indicates that the main unresolved challenge lies not in the production of biomaterial constructs per se, but in the reliable translation from printable formulations to functionally meaningful biological systems. This is especially evident in bioprinting, where the progression from bioinks to viable tissue analogues remains constrained by limited standardisation, incomplete biological validation, variable printability criteria and the difficulty of reproducing native tissue hierarchy. In this context, Ullah et al. [126] are particularly relevant, as they underscore that the path from printable bioinks to functional tissues and organs is still mediated by substantial scientific and translational gaps, especially regarding vascularisation, maturation, multicellular organisation and long-term functionality. The present review supports that position. While current bioprinting systems can produce increasingly sophisticated constructs, the fabrication of clinically relevant tissue substitutes still requires more robust control over microenvironmental cues, bioink composition, scaffold architecture and post-printing development.

A related limitation across the literature is the relatively weak connection between in vitro construct performance and in vivo clinical behaviour. Many studies report short-term cell viability, printability or preliminary mechanical stability, but fewer examine host response, immune modulation, vascular integration, degradation under physiological loading, infection susceptibility or long-term functional recovery. This gap is particularly important because constructs that perform adequately in static in vitro assays may behave differently under perfusion, cyclic loading, enzymatic degradation, immune–cell interaction or patient-specific pathological conditions. Consequently, future studies should place greater emphasis on longitudinal in vivo assessment, clinically relevant animal models, tissue-specific functional outcomes and, where available, carefully reported clinical case studies. Such evidence is necessary to determine whether AM biomaterials provide durable biological benefit beyond geometrical customisation or scaffold fabrication.

Another important trend emerging from the literature is the growing relevance of computational and data-driven strategies in overcoming these limitations. The inclusion of Grira et al. [127] is therefore particularly pertinent, because it reflects a broader shift towards artificial-intelligence-assisted optimisation of natural-material bioinks and printability behaviour. This development is not merely methodological; it signals a change in how the field may address its intrinsic complexity. Biomaterial-based AM involves a large number of coupled variables—rheology, concentration, thermal history, curing parameters, geometry, cell loading and degradation response—which are difficult to optimise through conventional trial-and-error approaches alone. AI-assisted modelling, predictive analytics and process-aware optimisation may therefore become essential tools for improving reproducibility, reducing empirical uncertainty and accelerating formulation-to-application development, particularly in biofabrication systems based on natural polymers and biologically sensitive materials.

From a broader engineering and translational standpoint, the present review therefore suggests that the future of AM with biomaterials will depend less on isolated advances in any single material or technique, and more on the coordinated integration of material science, process engineering, biological validation and application-specific design. The field is already moving beyond proof-of-concept fabrication towards the more demanding objective of producing constructs that are not only printable, but also biologically competent, mechanically reliable, clinically relevant and manufacturing-consistent. For this reason, future progress will depend on the establishment of more rigorous validation frameworks, clearer printability and biocompatibility criteria, and stronger convergence between post-processing science, surface engineering, bioink design and computational optimisation.

### 4.2. Process–Structure–Property Relationships

**Figure 11:**
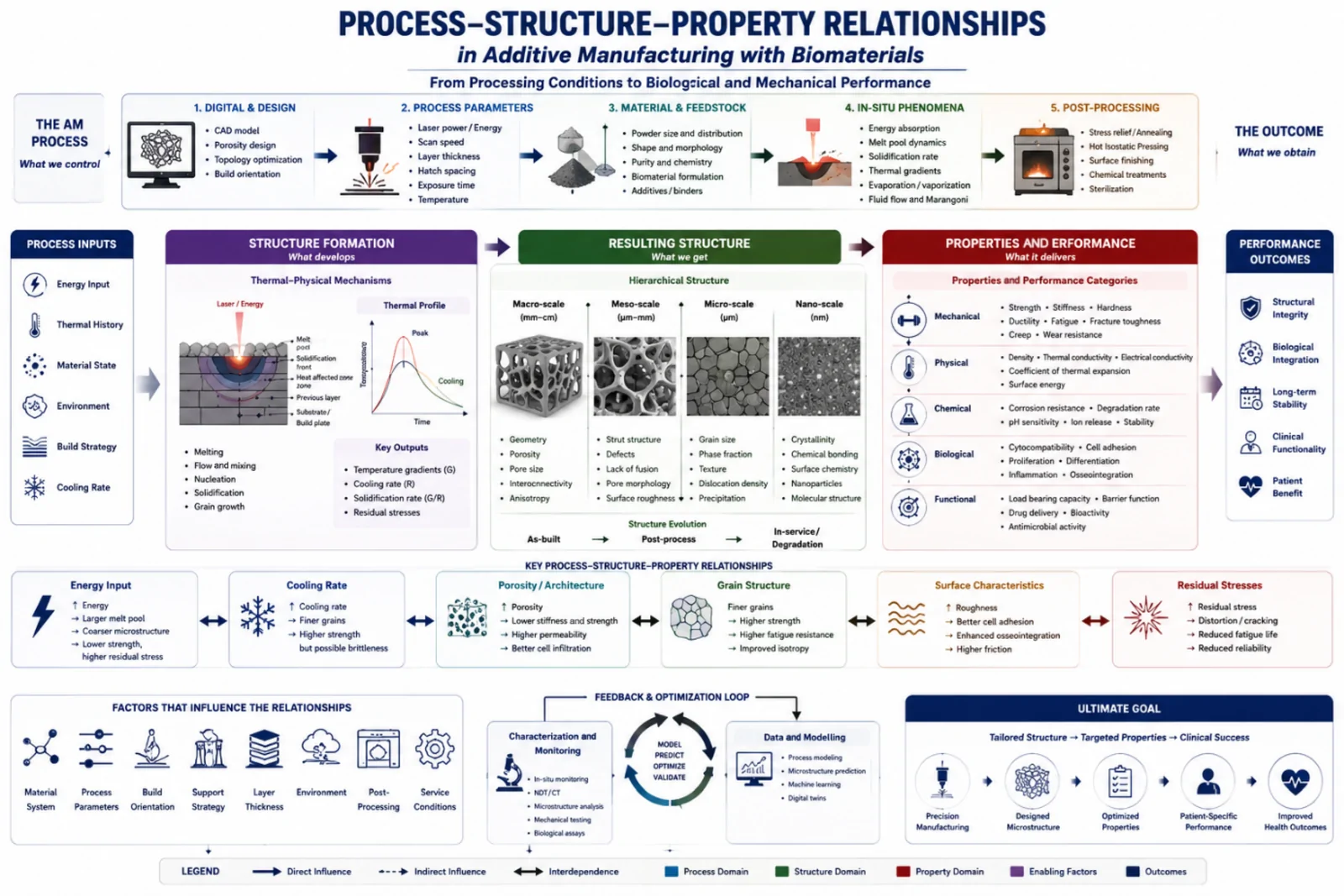
Process–structure–property relationships in AM of biomaterials, illustrating the multiscale interactions between process parameters, thermal and physicochemical phenomena, hierarchical structural evolution, and the resulting mechanical, biological, chemical and functional properties governing biomedical performance and clinical applicability (figure done in Microsoft^®^ Visio Pro 2021).

One of the most fundamental scientific challenges in AM of biomaterials concerns the complex interdependence between processing conditions, microstructural evolution, and the resulting functional performance of the fabricated constructs. Unlike conventional manufacturing routes, AM technologies generate highly localised and dynamic thermal, mechanical and physicochemical conditions capable of profoundly influencing material organisation across multiple length scales. Consequently, the final biological, mechanical, chemical, and functional properties of AMed biomaterials are not determined solely by material composition, but rather emerge from intricate process–structure–property interactions involving process parameters, feedstock characteristics, thermal history, solidification behaviour, defect formation and post-processing strategies. Understanding these multidimensional relationships is therefore essential for achieving reliable process optimisation, reproducibility, long-term structural integrity and clinically relevant biological performance. Figure 11 summarizes the principal process–structure–property relationships governing AM of biomaterials, highlighting the dynamic pathways linking manufacturing conditions to hierarchical structural development and final biomedical functionality.

The framework presented in Figure 11 demonstrates that AM performance cannot be interpreted through isolated processing variables or single-property evaluations alone. Instead, the final behaviour of biomaterial constructs emerges from highly interconnected multiscale interactions involving thermal gradients, cooling kinetics, porosity evolution, grain morphology, surface characteristics, residual stresses, and post-processing transformations. Importantly, many of these interactions exhibit nonlinear and process-dependent behaviour, making predictive optimisation particularly challenging within complex biomedical manufacturing environments. The increasing integration of real-time monitoring systems, digital twins, machine learning, and advanced characterisation techniques is therefore expected to play a decisive role in improving process predictability and structure–property control.

Zhang et al. [128] investigated the challenge of improving impact toughness in SLM-fabricated CoCrMo components by relating annealing temperature, fracture morphology and fractal dimension. It was determined that 1200 °C annealing produced the highest fractal dimension and impact toughness, establishing fractal analysis as a useful performance indicator. Furthermore, a deeper understanding of these relationships will be essential for enabling the development of next-generation biomaterials exhibiting simultaneously optimised mechanical reliability, biological functionality, degradation behaviour and patient-specific performance within translational biomedical applications.

Process–structure relationships represent a central mechanism through which AM decisions are translated into biomaterial performance. In scaffold-based systems, pore size, pore interconnectivity, strut thickness and architectural regularity influence not only stiffness and compressive strength, but also nutrient diffusion, vascular ingrowth, degradation behaviour and tissue infiltration. Highly porous architectures may reduce stiffness mismatch and improve biological integration, particularly in bone-oriented constructs, but excessive porosity or poorly connected pore networks can reduce load-bearing capacity and increase the risk of premature failure. Similarly, layer orientation and deposition path affect anisotropy, interfacial bonding and crack propagation, which are especially relevant for extrusion-based polymeric scaffolds and load-bearing metallic implants.

In metal AM, thermal history governs melt-pool geometry, cooling rate, residual stress, grain morphology, porosity and surface roughness, thereby influencing fatigue behaviour, corrosion response, ion release and osseointegration. In contrast, light-based processes are governed by photopolymerisation kinetics, where light penetration, conversion degree, exposure time, photoinitiator chemistry and post-curing determine resolution, network formation, residual monomer content and cytocompatibility. These examples show that process parameters should not be interpreted as manufacturing settings alone; they are upstream determinants of construct architecture, mechanical reliability and biological performance.

### 4.3. Emerging Paradigms and Future Directions

**Figure 12:**
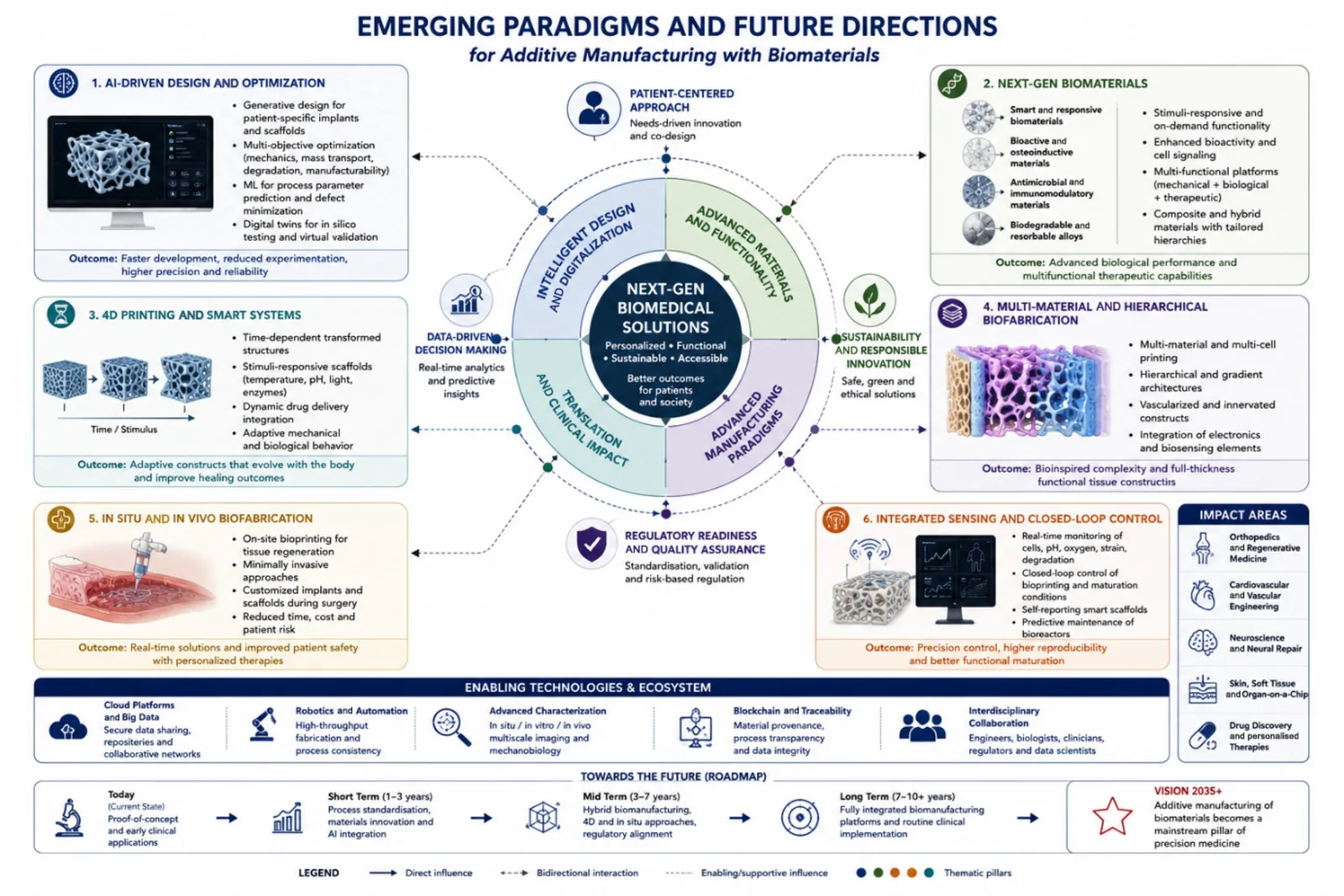
Emerging paradigms and future directions in AM for biomaterial applications, illustrating the convergence between AI-driven design, advanced biomaterials, 4D printing, in situ biofabrication, integrated sensing systems, translational medicine, sustainability-oriented innovation and next-generation biomedical manufacturing ecosystems (figure done in Microsoft^®^ Visio Pro 2021).

The rapid evolution of AM for biomaterial applications is progressively reshaping the boundaries of biomedical engineering, regenerative medicine and patient-specific healthcare solutions. Beyond conventional scaffold fabrication, emerging paradigms increasingly integrate artificial intelligence, smart biomaterials, digital twins, multi-material architectures, real-time sensing and advanced biofabrication strategies into highly interconnected translational ecosystems. These developments are driving a transition from static manufacturing approaches toward adaptive, intelligent and functionally responsive biomedical systems capable of dynamically interacting with biological environments and clinical requirements. Simultaneously, growing demands for regulatory robustness, process reproducibility, sustainability and scalable clinical implementation are accelerating the convergence between materials science, computational engineering, biotechnology and precision medicine. Figure 12 summarizes the principal technological paradigms and future research directions expected to shape the next generation of AM platforms for advanced biomaterial engineering and biomedical applications.

The emerging paradigms illustrated in Figure 12 demonstrate that the future of AM in biomaterials will likely depend on the integration of intelligent design methodologies, multifunctional biomaterials, predictive digital infrastructures and clinically oriented translational frameworks. In particular, the increasing incorporation of AI-assisted optimisation, in situ biofabrication, 4D printing concepts and closed-loop monitoring systems is expected to substantially improve manufacturing precision, biological functionality and patient-specific adaptability. Nevertheless, significant challenges remain regarding process standardisation, long-term biological validation, regulatory harmonisation, ethical considerations and large-scale manufacturing reproducibility. Consequently, future advances in this field will require highly interdisciplinary collaboration capable of simultaneously addressing engineering, biological, computational, clinical and regulatory dimensions within a unified translational ecosystem.

### 4.4. Regulatory and Translational Bottlenecks

**Figure 13:**
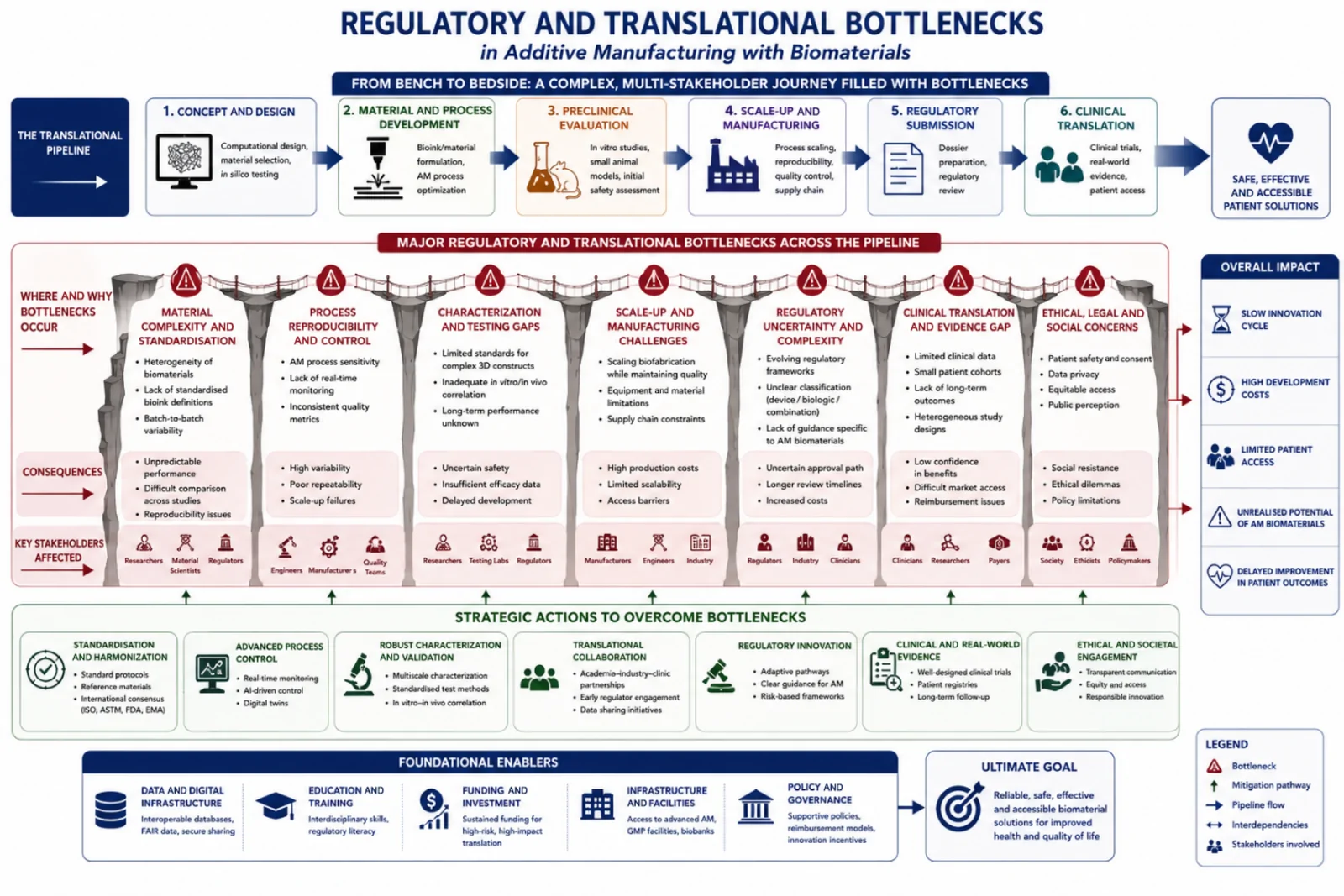
Regulatory and translational bottlenecks associated with AM of biomaterials, illustrating the principal scientific, technological, manufacturing, regulatory, clinical, ethical and organisational barriers affecting the successful transition from laboratory-scale research to clinically validated and commercially scalable biomedical solutions (figure done in Microsoft^®^ Visio Pro 2021).

Despite the remarkable scientific and technological advances achieved in AM for biomaterial applications, the effective translation of laboratory-scale developments into clinically validated and commercially viable healthcare solutions remains constrained by numerous regulatory, technical, economic and organisational barriers. While substantial progress has been achieved regarding scaffold design, biomaterial engineering, process precision and biological functionality, many emerging technologies still face significant difficulties associated with process reproducibility, quality assurance, large-scale manufacturing, long-term validation, and regulatory acceptance. Furthermore, the highly interdisciplinary nature of AM in biomedical environments introduces additional complexity involving material characterisation, in vitro and in vivo correlation, patient safety, ethical considerations and compliance with evolving international standards. Figure 13 summarizes the principal regulatory and translational bottlenecks currently limiting the large-scale clinical implementation of AMed biomaterial systems, while simultaneously identifying the strategic pathways required to accelerate safe, reliable and sustainable translational adoption.

Ethical and regulatory issues are particularly relevant when AM biomaterials incorporate patient-specific imaging data, autologous or allogeneic cells, bioactive molecules, degradable implants or personalised device geometries. In these cases, clinical translation requires not only demonstration of material safety and manufacturing reproducibility, but also traceability of digital design files, control of batch-to-batch variability, sterility assurance, informed consent for patient-derived data or cells and clear definition of responsibility across the design–manufacture–clinical-use chain. These requirements become even more demanding for bioprinted or living constructs, where cell sourcing, donor variability, long-term biological behaviour and post-implantation monitoring raise additional ethical and regulatory questions.

The framework presented in Figure 13 highlights that the successful clinical translation of AM technologies depends not only on scientific innovation, but also on the establishment of integrated translational ecosystems capable of connecting engineering development, regulatory science, industrial scalability, clinical validation and societal acceptance. In particular, the literature increasingly demonstrates that process standardisation, robust quality-control systems, advanced characterisation methodologies, real-time monitoring infrastructures and early regulatory alignment constitute critical enabling factors for reducing translational uncertainty. Simultaneously, stronger collaboration between academia, industry, clinicians, regulatory agencies and policymakers will be essential for overcoming fragmentation across the translational pipeline. Consequently, future progress in biomaterial AM will likely depend on the development of adaptive and interdisciplinary governance models capable of balancing technological innovation, patient safety, manufacturing reproducibility and long-term clinical reliability within rapidly evolving biomedical ecosystems.

Translation of AM biomaterials also depends on practical manufacturing constraints that are frequently underdeveloped in the literature. Sterilisation is a major example, because heat, radiation, ethylene oxide, plasma or chemical sterilisation may alter polymer degradation, hydrogel swelling, ceramic surface chemistry, metallic oxide layers, residual monomers, mechanical properties or cell-compatible functionality. Consequently, sterilisation should be validated as part of the material–process–post-processing chain rather than treated as a final administrative requirement. Similarly, manufacturing reproducibility remains difficult because small variations in powder morphology, bioink formulation, crosslinking kinetics, humidity, temperature, build orientation or post-curing can modify construct architecture and biological response.

Scale-up introduces further barriers. Laboratory-scale constructs are often produced under tightly controlled conditions, using small batches and short validation windows, whereas clinical translation requires batch-to-batch consistency, traceable digital workflows, validated quality-control procedures, robust sterility assurance, reproducible biological performance and compliance with regulatory expectations for patient-specific or biologically active devices. These requirements are particularly demanding for cell-laden constructs, where donor variability, cell sourcing, phenotype stability, maturation time and post-implantation monitoring complicate conventional device-approval pathways. Therefore, translational readiness requires integrated evidence across manufacturing repeatability, biological safety, long-term function and regulatory acceptability.

## 5. Conclusions

AM has progressively evolved from a prototyping-oriented fabrication approach into a transformative technological paradigm capable of fundamentally reshaping biomaterials engineering, regenerative medicine, and patient-specific healthcare solutions. The convergence between advanced manufacturing technologies, biomaterial science, digital engineering, and translational medicine has enabled the fabrication of highly complex, biologically functional, and structurally optimised constructs exhibiting unprecedented levels of geometrical customisation and functional adaptability. Across the different AM routes analysed throughout this review, significant advances have been achieved regarding scaffold architecture control, microstructural tailoring, multi-material integration, surface functionalisation and biomimetic tissue fabrication, substantially expanding the application potential of biomaterials within orthopaedic, dental, cardiovascular and tissue-engineering domains.

Nevertheless, this review also demonstrates that no individual AM technology currently satisfies all the mechanical, biological, manufacturing, economic, and translational requirements simultaneously demanded by advanced biomedical applications. Each manufacturing route presents highly specific advantages, limitations, and process-dependent trade-offs involving dimensional precision, surface quality, process scalability, biological compatibility, thermal stability, structural reliability, and regulatory feasibility. Consequently, the successful implementation of AM in biomaterial engineering increasingly depends on multidimensional decision-support strategies capable of integrating biomaterial characteristics, process capabilities, clinical requirements and translational objectives within unified engineering frameworks. In this context, the decision-support and translational ecosystem frameworks proposed throughout this work contribute toward a more systematic understanding of the complex interactions governing AM technology selection and biomedical applicability.

The analysis of process–structure–property relationships further highlights that the final performance of AMed biomaterials emerges from highly interconnected multiscale phenomena involving thermal history, material feedstock characteristics, solidification behaviour, porosity evolution, residual stresses, surface morphology, degradation kinetics and biological interactions. Such complexity reinforces the importance of integrated process optimisation strategies capable of simultaneously controlling mechanical integrity, biological functionality, long-term reliability and manufacturing reproducibility. The growing incorporation of artificial intelligence, machine learning, digital twins, in situ sensing systems and predictive modelling methodologies is expected to play a decisive role in improving process stability, adaptive manufacturing control, defect prediction and structure-function optimisation within future biomedical manufacturing ecosystems.

Despite the extraordinary technological progress observed over the last decade, the large-scale clinical translation of AM for biomaterial applications remains constrained by several critical bottlenecks involving regulatory approval, standardisation, reproducibility, quality assurance, sterilisation compatibility, scalability and long-term biological validation. In particular, the increasing complexity associated with patient-specific devices, cell-laden constructs, multifunctional biomaterials and hybrid manufacturing platforms introduces substantial challenges regarding certification pathways, ethical governance and translational reliability. Therefore, future progress in this field will strongly depend on the establishment of integrated translational infrastructures capable of connecting academic research, industrial manufacturing, regulatory science, clinical validation and post-market surveillance within adaptive and multidisciplinary biomedical ecosystems.

Simultaneously, emerging paradigms such as 4D printing, smart biomaterials, bioresponsive systems, autonomous manufacturing platforms, AI-assisted design and in situ bioprinting are progressively redefining the future boundaries of biomedical manufacturing. These technologies are expected to enable the development of dynamically responsive biomaterial systems capable of adapting to physiological environments, promoting tissue regeneration and supporting highly personalised therapeutic strategies aligned with the principles of precision medicine. Furthermore, the integration of sustainability-oriented manufacturing approaches, circular material strategies and resource-efficient fabrication methodologies will likely become increasingly important as AM technologies continue to expand toward industrial-scale biomedical implementation.

Overall, this review demonstrates that the future of AM of biomaterials will not be defined solely by incremental improvements in fabrication technologies, but rather by the successful convergence of intelligent design methodologies, advanced biomaterials, translational reliability, regulatory robustness and patient-centred clinical integration within highly interconnected biomedical innovation ecosystems. The transition from experimental biofabrication toward clinically validated and commercially sustainable healthcare solutions will therefore require not only technological excellence, but also the establishment of collaborative and multidisciplinary translational frameworks capable of harmonising engineering innovation, biological complexity, manufacturing reproducibility and long-term therapeutic safety.

The core consensus emerging from this review is that AMed biomaterials should be understood as integrated material–process–structure–property–biology–translation systems, in which clinical value depends not only on printability or geometrical complexity, but also on reproducible manufacturing, controlled post-processing, validated biological performance and regulatory readiness.

## 6. Future Directions

The future evolution of AM for biomaterial applications is expected to extend far beyond the current paradigm of geometrical customisation and layer-by-layer fabrication, progressively transitioning toward highly intelligent, adaptive, multifunctional and clinically integrated biomedical manufacturing ecosystems. Although substantial advances have already been achieved regarding scaffold engineering, biofabrication strategies and patient-specific device manufacturing, future research efforts will increasingly focus on establishing deeper convergence between materials science, artificial intelligence, computational engineering, regenerative medicine and translational healthcare infrastructures.

To consolidate the future-oriented synthesis, Table 5 summarises the principal unresolved challenges identified across the reviewed literature and proposes corresponding research or translational strategies.

The future directions identified in Table 5 can be organised into three complementary development horizons. In the short term, progress is expected to depend on technological optimisation, including better biomaterial-specific processing windows, improved bioink rheology, more reliable pore-architecture design, reduced post-processing-induced variability and stronger in-process monitoring. These advances are required to improve printability, structural fidelity, cytocompatibility and reproducibility across existing AM platforms. In the medium to long term, the field must move from proof-of-concept constructs towards clinically validated biomedical systems, particularly through improved vascularisation strategies, maturation protocols for cell-laden constructs, longitudinal in vivo validation, patient-specific design workflows, scalable manufacturing routes and integration with digital twins or AI-assisted optimisation. In parallel, standardisation must evolve as a distinct development pathway, including harmonised reporting of material formulations, processing parameters, biological endpoints, post-processing conditions, sterilisation procedures, quality-assurance metrics and regulatory evidence. These three horizons indicate that the future of AMed biomaterials will depend not only on new printing capabilities, but also on coordinated progress in technological robustness, clinical validation and standardised translational governance.

One of the most promising future directions involves the integration of artificial intelligence and machine learning into AM workflows. AI-assisted systems are expected to significantly improve process optimisation, defect prediction, adaptive parameter control, topology optimisation and predictive maintenance of manufacturing platforms. Simultaneously, machine learning algorithms capable of correlating process parameters, thermal history, microstructural evolution, and biological performance may enable the development of highly predictive manufacturing environments with enhanced reproducibility, quality assurance, and translational reliability. The emergence of digital twins and real-time process monitoring systems will further strengthen this transition toward intelligent and autonomous biomedical manufacturing ecosystems.

Another major future direction concerns the rapid development of smart biomaterials and 4D printing technologies capable of dynamically responding to physiological, mechanical, thermal, magnetic or biochemical stimuli. Unlike conventional static biomaterials, next-generation smart constructs are expected to exhibit adaptive behaviour, self-healing capability, controlled drug release, shape-memory effects and environment-responsive functionality, substantially expanding the potential applications of AM within tissue engineering and precision medicine. In particular, the integration of bioresponsive materials with programmable architectures may enable the fabrication of dynamic biomedical systems capable of evolving after implantation according to patient-specific biological conditions [129,130].

Future advances in bioprinting are also expected to focus strongly on vascularisation strategies, multicellular integration and functional tissue maturation. Although current bioprinting approaches already demonstrate remarkable potential for tissue engineering applications, the fabrication of clinically relevant large-scale tissues and fully functional organs remains one of the greatest unresolved scientific challenges in regenerative medicine. Consequently, future investigations will likely emphasise the development of hierarchical vascular networks, biomimetic extracellular matrices, advanced bioinks, stem-cell engineering strategies and hybrid biofabrication platforms capable of improving long-term cellular viability, tissue integration and physiological functionality.

At the manufacturing level, future AM systems will likely become increasingly hybridised, combining multiple fabrication principles within integrated platforms capable of simultaneously processing metals, ceramics, polymers, hydrogels and living cells. Such hybrid manufacturing environments may substantially improve multifunctionality, interfacial control, mechanical performance and biological integration, enabling the fabrication of highly complex biomedical constructs exhibiting graded architectures and multifunctional behaviour. Moreover, the integration of nanotechnology, bioactive surface engineering and advanced coating strategies is expected to further enhance osseointegration, antimicrobial performance, cellular adhesion and long-term implant reliability.

Regulatory science and translational validation will also constitute critical future research priorities. As AM technologies continue progressing toward clinical implementation, increasing emphasis will be placed on standardisation methodologies, reproducibility assessment, in situ quality monitoring, long-term biological validation, sterilisation compatibility and regulatory harmonisation across international healthcare systems. The development of globally accepted certification frameworks specifically adapted to patient-specific and biofabricated medical devices will likely become essential for accelerating safe and scalable clinical adoption.

Sustainability will additionally emerge as a progressively important research direction within AM of biomaterials. Future efforts are expected to prioritize resource-efficient manufacturing strategies, recyclable biomaterials, powder reuse optimisation, energy-efficient processing routes, and circular-economy-oriented biomedical production systems. The combination of sustainable manufacturing principles with advanced biomaterial engineering may contribute toward reducing environmental impact while simultaneously improving manufacturing efficiency and healthcare accessibility.

Ultimately, the future of AM for biomaterial applications will likely be characterised by the convergence of intelligent manufacturing, adaptive biomaterials, precision medicine, and digitally interconnected healthcare ecosystems. The successful realisation of this vision will require highly interdisciplinary collaboration involving engineers, clinicians, material scientists, computational researchers, regulatory agencies and industrial stakeholders working collectively toward the development of safer, smarter, more personalised and clinically reliable biomedical solutions. In this context, AM is expected to progressively evolve from an advanced fabrication technology into a foundational pillar of next-generation translational medicine and patient-centred healthcare innovation.

## Figures and Tables

**Table 1:** Terminology adopted in this review for additive manufacturing, 3D printing, biofabrication and bioprinting, with emphasis on their scope, biological content and use within biomaterial-based manufacturing.

Term	Use in This Review
AM	Umbrella term for layer-wise fabrication of biomaterials, including acellular and cellular routes.
3D printing	General synonym for AM, used mainly for broader or non-specialist references and when retained from cited literature.
Biofabrication	Broader biological manufacturing of constructs containing biomaterials, cells, bioactive molecules or tissue-like architectures.
Bioprinting	Cell-containing or bioink-based AM process involving controlled deposition of living cells and/or biologically active materials.

**Table 2:** Comparative overview of principal biomaterial classes used in AM for biomedical applications.

Biomaterial Class	Mechanical Properties	Biocompatibility	Printability	Clinical Applications	Main Limitations
Metals and alloys	High strength, stiffness, fatigue resistance and load-bearing capacity; elastic modulus often higher than bone	Generally suitable when alloy chemistry, passive-film stability, corrosion behaviour, ion release and surface condition are controlled	Highly compatible with PBF, DED and BJ; requires powder quality control and thermal-process optimisation	Orthopaedic implants, dental implants, craniofacial devices, porous fixation structures and load-bearing scaffolds	Stress shielding, corrosion, ion release, fatigue sensitivity, high processing temperature and demanding post-processing
Ceramics and bioceramics	High compressive strength and stiffness; brittle behaviour and low tensile toughness	Often bioinert or bioactive; calcium phosphates and hydroxyapatite support bone bonding and osteoconduction	Printable by BJ, vat photopolymerisation, extrusion-based slurry systems and indirect approaches	Bone substitutes, dental components, coatings, porous scaffolds and mineralised-tissue repair	Brittleness, shrinkage during sintering, limited damage tolerance, difficult densification and risk of pore-architecture distortion
Polymers	Broad range from flexible to rigid; generally lower stiffness and strength than metals and ceramics	Many biodegradable and bioresorbable systems show favourable tissue compatibility	Highly compatible with ME, vat photopolymerisation, MJ and bioprinting-compatible formulations	Tissue-engineering scaffolds, drug delivery, anatomical models, temporary implants and soft-tissue supports	Limited load-bearing capacity for many systems, thermal or curing sensitivity, degradation-control challenges, sterilisation effects, and possible residual monomer, solvent or additive toxicity
Hydrogels and bioinks	Soft, hydrated and tissue-like; low intrinsic mechanical strength unless reinforced or crosslinked	High potential for cell encapsulation, nutrient diffusion and tissue-mimetic environments	Suitable for extrusion, inkjet, laser-assisted and vat-based bioprinting, depending on rheology and crosslinking	Cell-laden constructs, soft-tissue models, organ-mimetic systems, wound healing and regenerative medicine	Poor mechanical stability, narrow rheological window, limited vascularisation, cell sensitivity to shear/light exposure and maturation challenges
Composites and biocomposites	Tailorable behaviour through reinforcement, fillers or hybrid phases; can combine stiffness, toughness and bioactivity	Depends on matrix–reinforcement compatibility, degradation products and surface response	Printable by extrusion, BJ, PBF, vat photopolymerisation and hybrid routes, depending on formulation	Bone scaffolds, dental materials, tissue-engineering matrices, bioactive implants and patient-specific constructs	Interfacial compatibility, heterogeneous degradation, particle dispersion, anisotropy, processing complexity and reproducibility

**Table 3:** Comparative overview of AM technologies used for biomaterial fabrication, including resolution, processing speed, cost, cell compatibility, post-processing requirements and clinical readiness [29].

Tech	Materials	Pros	Cons	Biomedical Application
ME	Hydrogels, thermoplastics, ceramics, bioinks	+ low cost + accessible + composite materials + open-source design	- slow - anisotropy in printed part - low resolution - nozzles impart high shear forces on cells	Bioprinting of scaffolds for cell culture Tissue and organ development Production of rigid and soft anatomical models for surgical planning
DED	Metal	+ fast + composite materials + dense part	- expensive - low resolution - requires post-processing/machining	Limited use in biomedical application
MJ (polyjet)	Photopolymer, bioinks	+ good resolution + good cell viability + multiple cell/material deposition	- slow - material waste - limited material selection - limited fabrication size	Bioprinting of scaffolds for cell culture tissue and organ development (soft tissue)
PBF	Thermoplastics, metal powders, ceramic powders	+ high strength and dense parts + fast + no solvents required + no support required	- most-expensive - post-processing required	Metallic implants Dental craniofacial and orthopaedic Temporary and degradable rigid implants
BJ	Metal, polymer, ceramics	+ low-cost + fast + multi-color printing + no support needed + large objects	- low strength - requires post-curing and post-processing - powder poses a respiratory hazard	Degradable metallic implants Generally used for hard, mineralised tissues
SLA	Photopolymer, bio-resin, ceramic resins	+ high resolution + fast + good cell viability + nozzle free	- raw material toxicity - limited material selection - possible harm to DNA by UV	Bioprinting of scaffolds for cell culture Tissue and organ development can be used for both soft and hard tissues
DLP	Photopolymer, bio-resin, ceramic resins			

**Table 4:** Comparative synthesis of material–process limitations and biological implications of AM routes for biomaterials.

AM Route	Principal Material–Process Limitation or Trade-Off	Biological or Translational Implication
BJ	Powder packing, binder distribution, low green strength and curing/infiltration/sintering determine porosity and dimensional stability.	Porosity preservation, residual binder removal and mechanical reinforcement govern degradation behaviour, tissue ingrowth and implant reliability.
DED	High thermal input, melt-pool history, low resolution and frequent need for machining or surface finishing affect microstructure and surface state.	Mainly relevant for metallic implants, coatings and repair strategies; residual stress, fatigue, corrosion and finishing remain critical for clinical use.
ME	Feedstock rheology, nozzle diameter, extrusion pressure and cooling or crosslinking determine filament stability, anisotropy and shear exposure.	Shape fidelity must be balanced against cell viability, heterogeneous cell distribution, post-print maturation and tissue-specific function.
MJ	Droplet generation, viscosity limits, deposition accuracy and restricted material range constrain resolution, construct thickness and multi-material deposition.	Useful for soft constructs and patterned deposition, but limited by cell-damage risk, restricted bioink applicability and reduced scalability towards clinically relevant volumes.
PBF	Energy density, powder morphology, thermal gradients, unmelted particles, residual stress and surface roughness determine density, fatigue behaviour and surface condition.	Strong potential for metallic implants, but fatigue sensitivity, ion release, corrosion risk, bacterial adhesion and osseointegration variability must be controlled.
VPP	Photopolymer chemistry, light exposure, conversion degree, residual monomers, photoinitiator toxicity and post-curing dependency determine resolution and cytocompatibility.	High-resolution scaffolds require control of cytotoxicity, degradation products, sterilisation compatibility, altered cell response and long-term biological safety.
Bioprinting	Bioink rheology, crosslinking strategy, cell density, nutrient transport, vascularisation and maturation conditions determine construct viability and function.	Translation depends on phenotype retention, immune response, vascular integration, long-term in vivo performance and reproducible clinically compliant workflows.

**Table 5:** Outstanding challenges and proposed solutions for AM of biomaterials.

Outstanding Challenge	Scientific or Translational Consequence	Proposed Solution or Research Direction
Limited material–process compatibility	Restricted printability, poor construct stability and inconsistent biological performance	Develop biomaterial-specific process windows linking rheology, powder behaviour, curing kinetics, thermal sensitivity and degradation profile
Narrow rheological window for bioinks	Collapse of low-viscosity constructs or shear-induced cell damage in highly viscous systems	Optimise shear-thinning behaviour, extrusion pressure, nozzle geometry, cell density and crosslinking sequence
Insufficient mechanical reliability of porous scaffolds	Poor load-bearing capacity, fatigue sensitivity or premature failure	Couple pore architecture design with mechanical testing, fatigue evaluation and patient-specific loading requirements
Incomplete biological validation	Weak correlation between in vitro performance and in vivo tissue response	Expand validation to include immune response, vascularisation, degradation kinetics, tissue-specific maturation and long-term animal studies
Post-processing effects on biological performance	Sintering, curing, sterilisation or chemical treatment may alter surface chemistry, porosity or cytocompatibility	Treat post-processing as part of the biomaterial design chain and validate its biological as well as mechanical consequences
Limited reproducibility and quality control	Batch-to-batch variability and uncertain regulatory acceptance	Implement standardised reporting, in-process monitoring, digital traceability, validated QA protocols and reproducible material specifications
Scale-up of cell-laden constructs	Laboratory constructs may not translate to clinically relevant dimensions or maturation states	Develop perfusion systems, vascularisation strategies, modular construct designs and controlled bioreactor maturation workflows
Regulatory and ethical uncertainty	Delayed translation of patient-specific, degradable or living constructs	Establish clearer pathways for patient-specific data, cell sourcing, sterility assurance, long-term monitoring and responsibility across the digital–clinical workflow
Sustainability and material circularity	Increased waste, limited lifecycle control and uncertain environmental performance	Integrate recyclable polymers, powder/feedstock reuse strategies, life-cycle assessment and environmentally responsible post-processing

**Figure A1:**
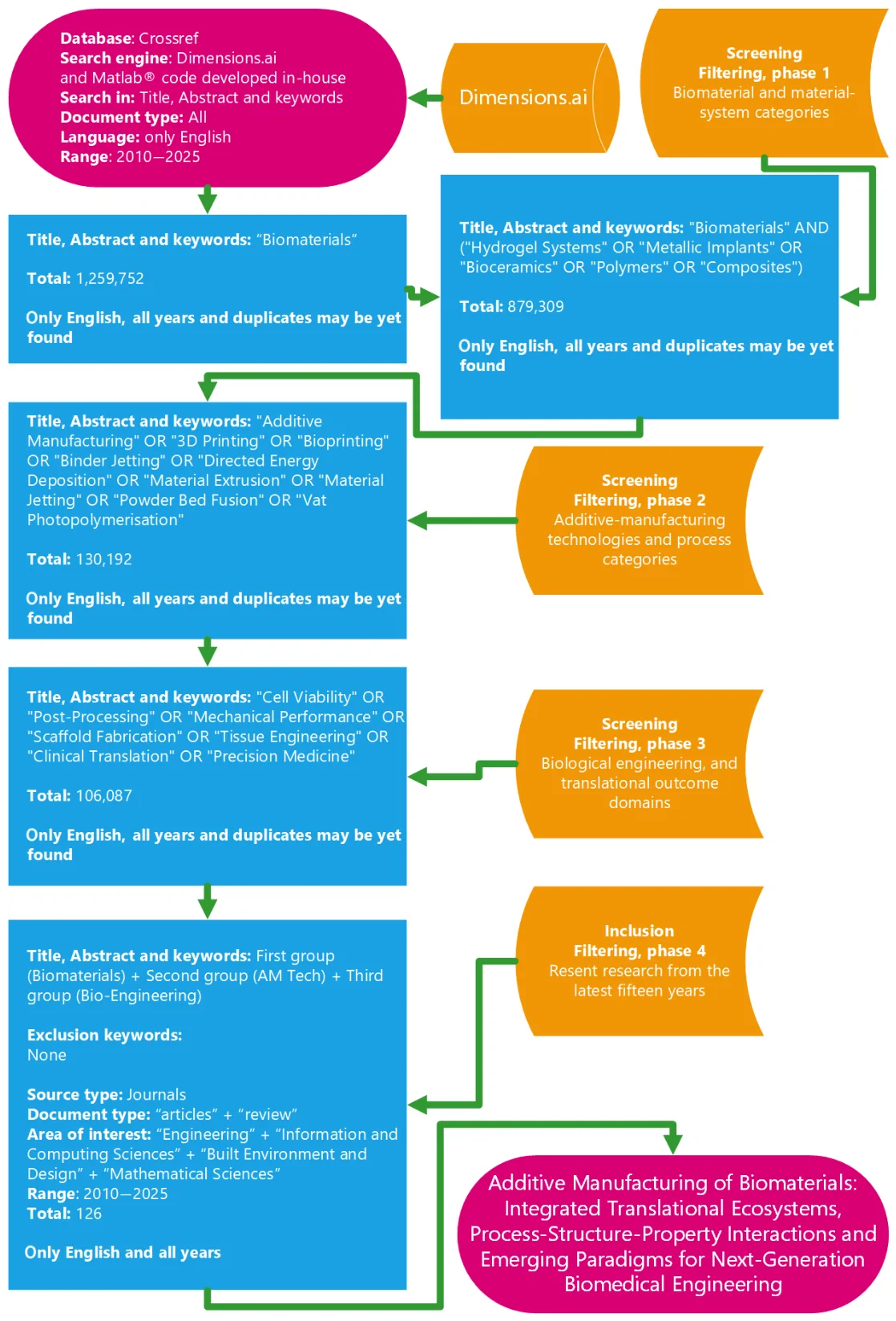
PRISMA-based multi-stage screening and study-selection procedure applied to the literature on biomaterials processed through additive-manufacturing routes, with particular emphasis on material classes, fabrication techniques, bioprinting strategies, post-processing requirements and the biomedical and translational relevance of the resulting constructs (figure done in Microsoft^®^ Visio Pro 2021).

**Figure A2:**
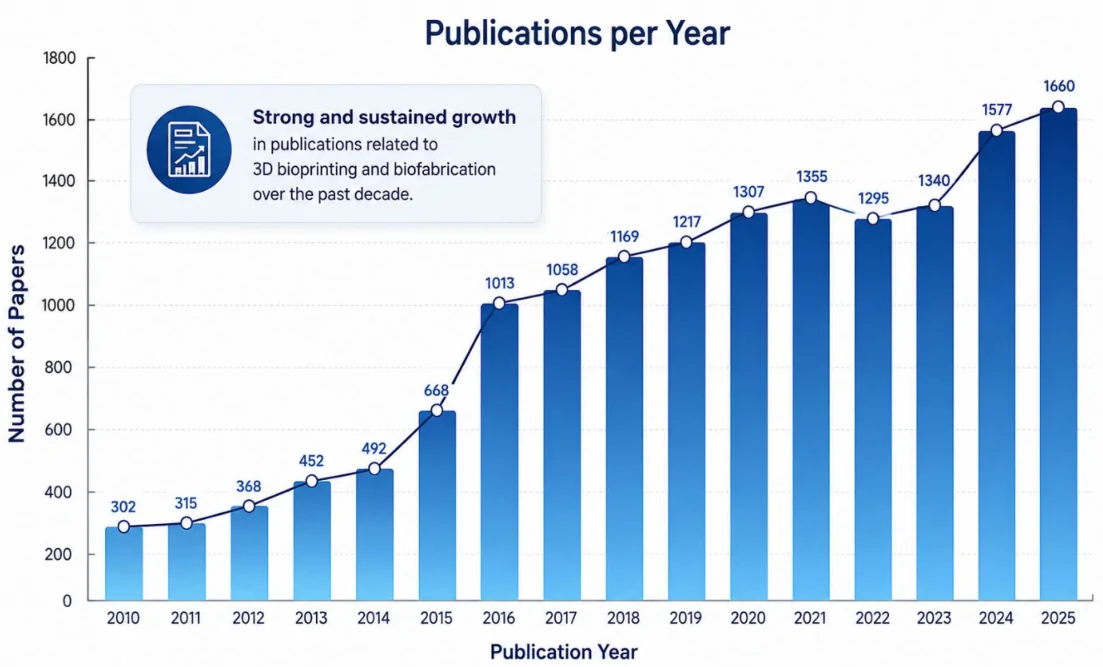
Annual publication trend for peer-reviewed records concerning AM of biomaterials and biomedical applications retrieved from the cleaned CrossRef dataset. The *x*-axis represents publication year from 2010 to 2025, and the *y*-axis represents the number of records retained after DOI-based deduplication, metadata harmonisation and publication-year normalization (figure done with Microsoft^®^ Excel and Microsoft^®^Visio Pro 2021).

**Figure A3:**
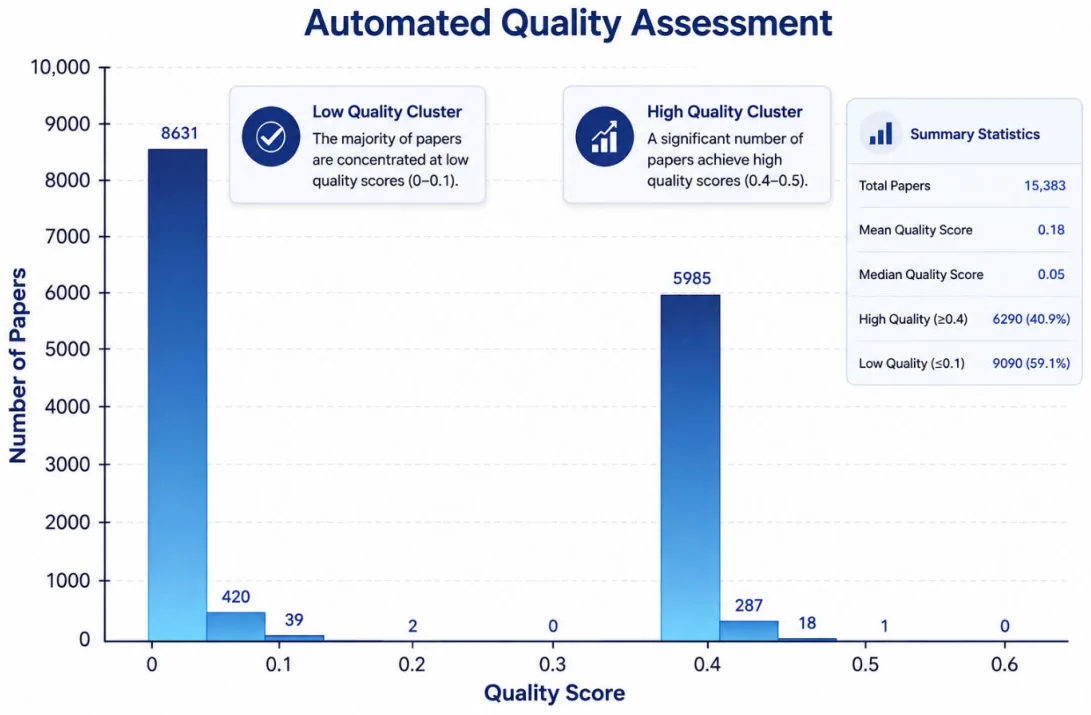
Preliminary quality-screening distribution obtained during metadata-assisted filtering of records related to additively manufactured biomaterials. The automated procedure was used only for field standardisation, duplicate detection and keyword-based prioritisation; final study-level QA decisions were performed manually by the authors during title, abstract and full-text assessment (figure done with Microsoft^®^ Excel and Microsoft^®^Visio Pro 2021).

**Figure A4:**
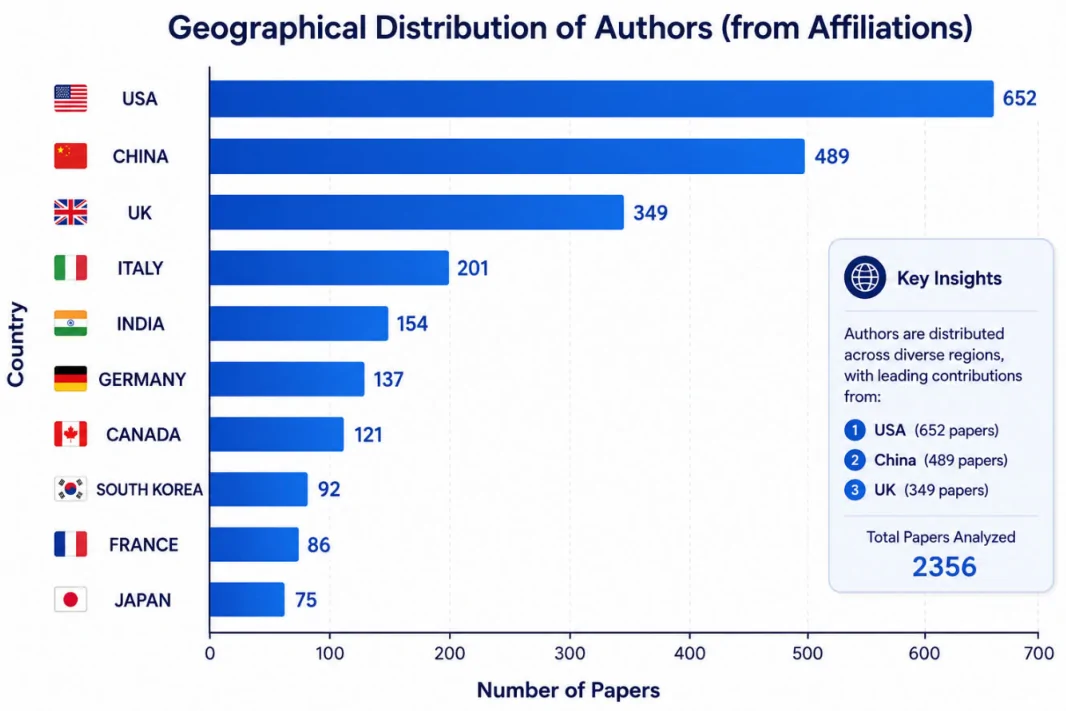
Geographical distribution of authors’ institutional affiliations across the publications included in the systematic review on biomaterials processed through additive-manufacturing routes, with particular emphasis on material classes, fabrication strategies, bioprinting approaches, post-processing requirements and biomedical applicability (figure done with Microsoft^®^ Excel and Microsoft^®^Visio Pro 2021).

**Figure A5:**
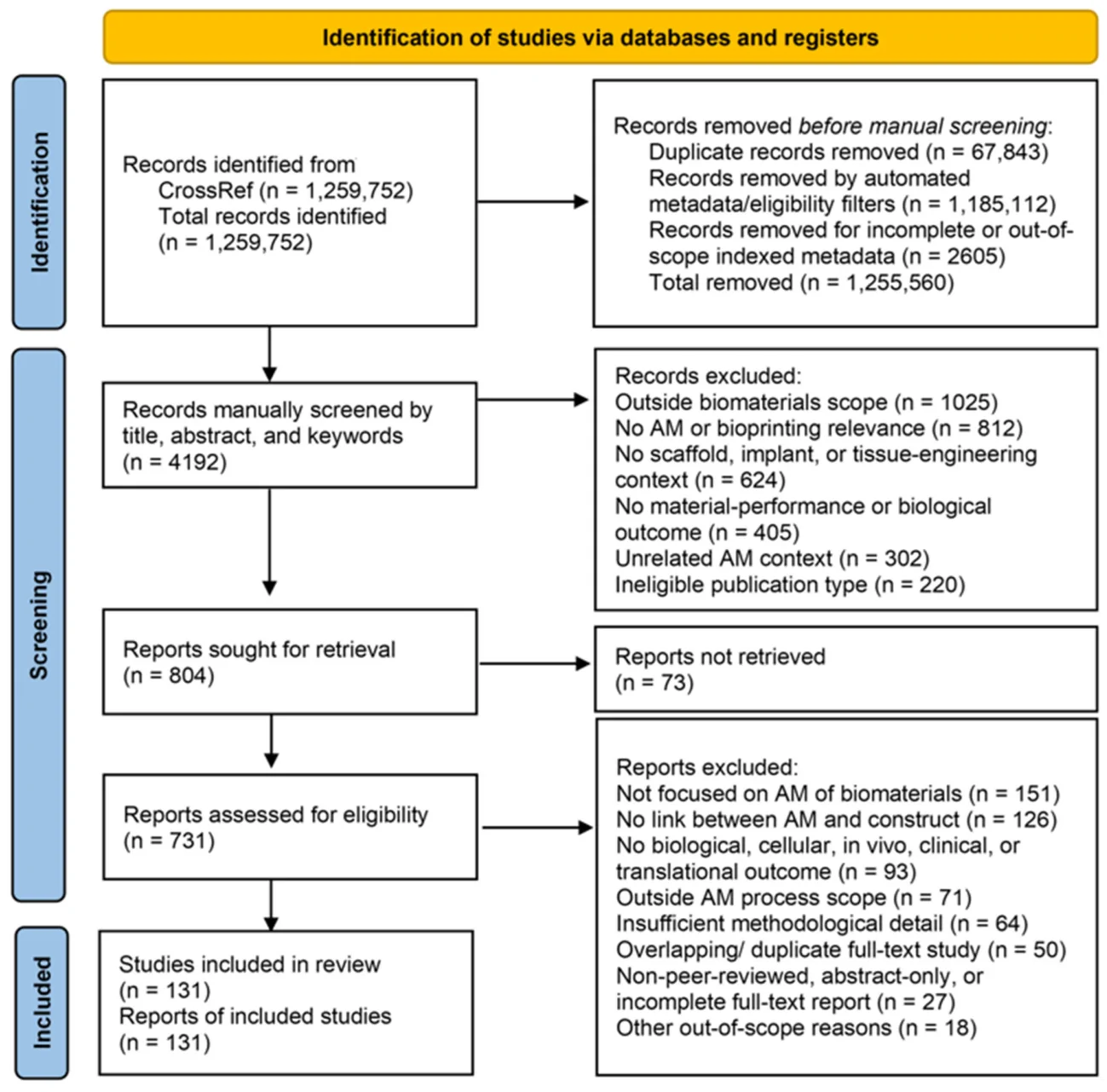
PRISMA 2020 flowchart summarising the identification, automated metadata-assisted filtering, manual screening, retrieval, eligibility assessment and final inclusion of studies in the review of AM of biomaterials. The initial database output represents the broad retrieval pool generated by the Boolean strategy. Automated processing was limited to deduplication, metadata harmonisation, language and document-type filtering and preliminary keyword-field eligibility checks. Final relevance and quality decisions were made manually by the authors through title/abstract/keyword screening and full-text eligibility assessment. Exclusion categories are reported as mutually exclusive groups according to the first applicable exclusion criterion (text and flowchart done in Microsoft^®^ Word and pasted as a figure).

## Data Availability

No new data were created or analysed in this study. Data sharing is not applicable to this article.

## Supplementary Materials

The following supporting information can be downloaded at: https://www.mdpi.com/article/10.3390/jfb17080365/s1. Supplementary Table S1. PRISMA 2020 Checklist.

## Appendix A

### Appendix A.1. Expanded Explanation on the Method of Research

This review was undertaken through a structured and transparent literature survey guided by the PRISMA 2020 framework [26,27], with the objective of ensuring methodological consistency, clarity and reproducibility throughout the processes of study identification, screening and selection. The retrieved publications were critically appraised according to their scientific relevance, technical contribution, methodological robustness and the academic standing of the publication outlets [131]. Bibliographic records were compiled in January 2026, principally from CrossRef, chosen for its extensive coverage of research relating to biomaterials, AM, tissue engineering and biomedical applications, and were complemented by additional searches in Dimensions.ai to broaden retrieval and enhance metadata completeness. To integrate records from the different databases, a bespoke MATLAB^®^ routine was used to automate data consolidation [132], standardise bibliographic fields and eliminate duplicate entries. This semi-automated approach improved both the efficiency and the reliability of the review process across the core topics addressed in the present study, including biomaterial classifications, hydrogel systems, cell viability, metallic implants, post-processing strategies and the wider clinical and technological implications of these approaches for precision medicine.

### Appendix A.2. Search Strategy and Information Sources

Building upon the PRISMA-based methodological framework outlined above, the literature search strategy was specifically designed to identify studies addressing AM in the field of biomaterials, with particular emphasis on biomaterial classes, processing routes, bioprinting approaches, biomedical applications and the principal technical and translational constraints affecting clinical implementation, rather than broader contributions on advanced manufacturing or biomedical engineering considered in isolation. The search protocol was structured to ensure thematic precision and to maximise the relevance of the retrieved literature through the use of three complementary conceptual groups of keywords.

Operationally, the retrieval strategy combined three Boolean blocks. The first block defined the biomaterial domain; the second block defined the AM or biofabrication route; and the third block defined the biomedical, biological or translational performance context. The search was therefore applied using the following logic:

(Biomaterial terms) AND (AM-technology terms) AND (biomedical/performance terms). The first group or biomaterial block included “*biomaterials*”, “*biocompatible materials*”, “*hydrogel systems*”, “*metallic implants*”, “*bioceramics*”, “*polymers*” and “*composites*”. The second group or AM-technology block included “*additive manufacturing*”, “*3D printing*”, “*bioprinting*”, “*binder jetting*”, “*directed energy deposition*”, “*material extrusion*”, “*material jetting*”, “*powder bed fusion*” and “*vat photopolymerisation*”. The third group or the biomedical/performance block included “*cell viability*”, “*post-processing*”, “*mechanical performance*”, “*scaffold fabrication*”, “*tissue engineering*”, “*clinical translation*” and “*precision medicine*”.

The Boolean search logic followed the general structure:

“Biomaterials” AND (“Hydrogel Systems” OR “Metallic Implants” OR “Bioceramics” OR “Polymers” OR “Composites”)

AND (“Additive Manufacturing” OR “3D Printing” OR “Bioprinting” OR “Binder Jetting” OR “Directed Energy Deposition” OR “Material Extrusion” OR “Material Jetting” OR “Powder Bed Fusion” OR “Vat Photopolymerisation”)

AND (“Cell Viability” OR “Post-Processing” OR “Mechanical Performance” OR “Scaffold Fabrication” OR “Tissue Engineering” OR “Clinical Translation” OR “Precision Medicine”).

Search queries were applied to the title, abstract and keyword fields of the selected databases. In order to ensure the scientific relevance, comparability and technical consistency of the collected literature, the following eligibility criteria were adopted:

- Publication period: 2010–2025
- Document type: peer-reviewed journal articles, review papers and selected conference papers of clear technical relevance
- Language: English

These criteria were introduced to ensure that the review captured both foundational and contemporary developments in the AM of biomaterials, particularly in relation to material selection, fabrication strategies, cell-compatible processing, post-processing requirements and the emerging role of these technologies in tissue engineering and precision medicine, whilst excluding publications falling outside the scope of biomaterial-based manufacturing and biomedical application.

### Appendix A.3. Study Selection and Screening

The identification and selection of relevant studies were undertaken in accordance with the PRISMA 2020 guidelines, thereby ensuring methodological transparency, traceability and reproducibility throughout the review process. The initial bibliographic search was conducted principally through the CrossRef database and supplemented by additional indexing platforms, most notably Dimensions.ai, yielding a substantial body of records spanning both foundational and contemporary contributions to biomaterials, additive-manufacturing technologies and biomedical fabrication. The large volume of initial results reflects, in part, the broad indexing coverage of CrossRef and related metadata repositories, which frequently include duplicate entries arising from publisher-level indexing, early-access records and overlaps between databases.

To address these redundancies, an automated consolidation procedure was implemented using a bespoke MATLAB^®^ script, supplemented by manual verification. This procedure harmonised bibliographic metadata across the different sources and enabled the systematic removal of duplicate records. The resulting dataset was then subjected to preliminary screening.

Screening and eligibility decisions were subsequently undertaken in accordance with predefined inclusion and exclusion criteria established prior to database interrogation, with the aim of minimising subjective bias and ensuring methodological consistency. Titles, abstracts and keywords were examined in order to assess their relevance to the AM of biomaterials, with particular emphasis on biomaterial classifications, fabrication strategies, bioprinting approaches, scaffold development, cell viability, post-processing requirements and the translational implications of these technologies for biomedical applications. Particular attention was given to studies addressing material–process compatibility, biological performance, mechanical behaviour, processing constraints, and the comparative capabilities of different additive-manufacturing routes in the fabrication of tissue-engineering constructs and implantable devices.

Publications lacking methodological clarity, those unrelated to biomaterial-based fabrication or studies that did not address additive-manufacturing techniques, biofabrication strategies, biomedical functionality or application-specific limitations were excluded at this stage. A considerable proportion of the discarded literature concerned either general manufacturing developments without direct biomedical relevance, or broader biomaterials studies that did not engage substantively with additive-manufacturing processes, scaffold fabrication, bioprinting performance or translational implementation.

The final stage involved a detailed full-text assessment of the remaining studies in order to determine their methodological robustness, technical relevance and conceptual coherence with the objectives of the present review. Following application of the established eligibility criteria, the final corpus comprised peer-reviewed publications considered sufficiently rigorous and relevant to support the analytical framework of the study. As a limited number of journals and publication venues accounted for a disproportionately large share of the retrieved literature, scientific relevance, technical depth and citation influence were also taken into consideration in order to reduce potential publication-source bias. The resulting dataset therefore represents a curated body of literature encompassing the most influential and methodologically robust studies concerning biomaterials, additive-manufacturing routes, scaffold fabrication, bioprinting systems and their biomedical applications.

During the review process, a recurrent challenge concerned the absence of fully standardised terminology within the literature. Identical or closely related processes are frequently described using different nomenclature, particularly in the case of additive-manufacturing techniques, biofabrication workflows and material classifications. For example, AM may also be reported as three-dimensional printing, whilst cellular fabrication strategies may appear under terms such as bioprinting, biofabrication or cell-laden printing. Comparable inconsistencies also arise in relation to biomaterial terminology, where expressions such as hydrogels, bioinks, polymeric systems, scaffold materials and tissue-engineering matrices are not always employed with strict definitional consistency. These variations required careful cross-verification of process descriptions, material functions and reported performance outcomes during the classification and interpretation of the retrieved studies. Minor orthographic differences between American and British English further introduced bibliographic inconsistencies in indexed metadata.

The formulation of precise research questions constitutes a fundamental element of any systematic literature review, as it defines the analytical scope and conceptual direction of the investigation. To ensure internal coherence and close alignment with the objectives of the present study, the Population–Intervention–Comparison–Outcome–Context framework was adapted as follows:

- Population: Experimental, computational and review studies concerned with biomaterials, additive-manufacturing technologies and biomedical fabrication, particularly those addressing scaffolds, tissue-engineering constructs, implantable devices and related clinical applications.
- Intervention: Additive-manufacturing and bioprinting strategies applied to biomaterials, including binder jetting, DED, ME, MJ, PBF, vat photopolymerisation and cellular fabrication approaches involving hydrogels and bioinks.
- Comparison: Comparative analyses between different biomaterial classes and additive-manufacturing routes, particularly with respect to cell viability, mechanical performance, resolution, post-processing demands, material compatibility and suitability for hard- and soft-tissue applications.
- Outcomes: Improvements in fabrication capability, scaffold quality, biological performance, structural integrity, and translational applicability, together with the identification of persistent technical limitations, processing constraints and unresolved research challenges affecting broader clinical implementation.
- Context: Peer-reviewed academic publications addressing biomaterials and additive-manufacturing technologies in biomedical engineering, tissue engineering and related translational domains.

Based on this framework, a structured and hierarchically organised set of research questions was established to guide the systematic extraction, critical evaluation and synthesis of the knowledge contained within the selected literature. These research questions are presented in Table A1 of the manuscript.

**Table A1:** PICOC RQs.

ID	Research Question
RQ1	What are the principal technical challenges affecting the manufacture, biological performance and practical applicability of biomaterials processed through additive-manufacturing routes?
RQ1.1	How do process-related limitations—such as restricted material–process compatibility, inadequate mechanical integrity, residual porosity, anisotropy, limited resolution, thermal degradation and reduced cell viability—manifest across different additive-manufacturing techniques, and how do they affect the functional performance of the resulting constructs?
RQ1.2	Which material characteristics, processing conditions and design-related factors, including rheology, viscosity, thermal sensitivity, powder morphology, curing behaviour, degradation profile, scaffold architecture and feedstock composition, exert the greatest influence on printability, structural fidelity, biological response and application suitability?
RQ2	How do material selection, fabrication route and post-processing strategy determine the final quality and usability of AMed biomaterial constructs?
RQ2.1	To what extent do biomaterial class, process selection and post-processing procedures improve dimensional fidelity, surface quality, mechanical performance, biological compatibility and confidence in the final construct for biomedical use?
RQ2.2	What are the principal trade-offs associated with AM of biomaterials, particularly in relation to accuracy, build rate, material versatility, cell viability, post-processing demands, cost and clinical applicability?
RQ3	How do process parameters, equipment configurations and technology selection influence the robustness, repeatability and biomedical performance of AMed biomaterials?
RQ3.1	Which operational variables—such as layer thickness, nozzle diameter, extrusion pressure, printing speed, laser or beam energy, powder-bed conditions, curing parameters and thermal history—most strongly affect print quality, mechanical behaviour, cell survival and scaffold integrity?
RQ3.2	How do different additive-manufacturing technologies and system architectures influence the applicability, effectiveness and limitations of biomaterial fabrication strategies used for hard- and soft-tissue engineering, implantable devices and related biomedical applications?
RQ4	What recent developments in biomaterial design, biofabrication methods and process optimisation strategies contribute to improved additive-manufacturing performance and translational potential?
RQ4.1	How can advances in hydrogel formulation, bioink engineering, multi-material processing, controlled porosity design and process-monitoring or optimisation approaches support improved printability, biological functionality, structural reliability and manufacturing efficiency?
RQ4.2	What technical, methodological and translational limitations currently hinder the broader implementation of advanced biofabrication strategies, particularly those involving cell-laden constructs, multi-material systems and patient-specific biomedical devices?
RQ5	What are the emerging research trends and future opportunities in the AM of biomaterials?
RQ5.1	Which advanced concepts—such as smart biomaterials, four-dimensional printing, bioactive and stimuli-responsive systems, hybrid scaffold architectures and patient-specific fabrication strategies—show the greatest potential for improving the clinical and technological relevance of this field?
RQ5.2	What key knowledge gaps remain regarding the integration of biomaterial design, additive-manufacturing process control, post-processing, biological validation and clinical translation in the development of next-generation biomedical constructs?

The present review considered peer-reviewed publications spanning both foundational and contemporary contributions to the AM of biomaterials, with particular emphasis on studies addressing biomaterial classes, fabrication routes, scaffold development, bioprinting strategies, post-processing requirements and biomedical performance. This temporal scope was selected in order to capture the technological evolution of biomaterial-based additive-manufacturing workflows, particularly with respect to acellular and cellular fabrication approaches, hydrogel-based systems, metallic implant production and the development of constructs intended for tissue engineering and related clinical applications. Particular attention was devoted to studies examining printability, cell viability, structural fidelity, mechanical competence, biological functionality, processing limitations and the suitability of different manufacturing routes for hard- and soft-tissue applications.

The literature search procedure, as illustrated in Figure A1, was implemented using structured Boolean queries designed to identify studies situated at the intersection of biomaterials research, additive-manufacturing technologies and biomedical application. The search strategy therefore incorporated combinations of keywords related to biomaterials and material systems, including “*biomaterials*”, “*hydrogel systems*”, “*metallic implants*”, “*bioceramics*”, “*polymers*” and “*composites*”, together with terms associated with additive-manufacturing technologies, such as “*additive manufacturing*”, “*3D printing*”, “*bioprinting*”, “*binder jetting*”, “*directed energy deposition*”, “*material extrusion*”, “*material jetting*”, “*powder bed fusion*” and “*vat photopolymerisation*”. A third group of terms addressed performance, processing and translational considerations, including “*cell viability*”, “*post-processing*”, “*mechanical performance*”, “*scaffold fabrication*”, “*tissue engineering*”, “*clinical translation*” and “*precision medicine*”.

The values reported in the corrected PRISMA workflow reflect the revised Boolean search strategy, in which “Biomaterials” was retained as the anchoring search term. The reduction from 1,259,752 identified records to 879,309 screened records resulted from automated metadata filtering using predefined eligibility criteria, whereas final relevance decisions were verified by the authors through title, abstract, keyword and full-text assessment.

This structured search strategy enabled the targeted retrieval of scientific contributions directly concerned with the AM of biomaterials, particularly studies addressing biomaterial classes, fabrication routes, scaffold development, bioprinting strategies, post-processing requirements and biomedical applicability. It was designed to capture literature relevant to material selection, process–material compatibility, construct fabrication, biological performance, and the comparative assessment of acellular and cellular manufacturing approaches. In this way, the search protocol supported a technically rigorous evaluation of the principal challenges, methodological trade-offs and emerging opportunities associated with the production of biomaterial-based constructs that are both functionally reliable and clinically relevant.

An examination of the publication records retrieved from the CrossRef repository indicates a marked increase in scholarly output between 2010 and 2025, as illustrated in Figure A2. This upward trajectory reflects the growing academic and industrial interest in biomaterial-based AM, particularly in relation to hydrogel systems, bioprinting platforms, scaffold fabrication, metallic implant production and the broader development of patient-specific biomedical constructs. The trend appears especially pronounced from the mid-2010s onwards, followed by a sustained period of elevated publication activity after 2020, thereby suggesting the consolidation of this field as an increasingly mature and strategically significant area of research and translational innovation.

Collectively, these developments underscore the growing recognition of AM with biomaterials as a critical enabling technology in tissue engineering, regenerative medicine and advanced biomedical device fabrication. Progress in material design, process control and post-fabrication optimisation is increasingly regarded as essential for improving structural fidelity, biological functionality, manufacturing reliability and the broader translational deployment of these technologies in clinically relevant settings.

### Appendix A.4. Study Selection and QA

The appraisal of methodological quality constitutes an essential component of any systematic review, since the credibility of the resulting synthesis depends directly upon the reliability, transparency and analytical robustness of the studies included. In the present investigation, a structured QA procedure was established to evaluate the scientific rigour, reporting clarity and technical relevance of each selected publication. The assessment strategy was conceived as a multidimensional framework capable of capturing not only academic robustness, but also the extent to which the reported findings contribute meaningfully to the understanding and advancement of biomaterials processed through additive-manufacturing routes.

The QA framework was organised into four domains intended to capture complementary dimensions of methodological and biomedical relevance: research design and conceptual coherence; definition of the biomaterial and manufacturing framework; methodological rigour and analytical transparency; and biomedical and translational relevance. Each domain was scored using a three-level ordinal scale, where 1 indicated that the criterion was fully satisfied, 0.5 indicated partial fulfilment and 0 indicated that the criterion was not adequately addressed. The final QA score was calculated as the sum of the four domain scores divided by the maximum possible score and expressed as a percentage.

Equal weighting was adopted because the review covered highly heterogeneous study types, biomaterial classes and AM routes, including metallic implants, ceramic scaffolds, polymeric constructs, composites, hydrogels and bioprinting systems. In this context, assigning a higher weighting to one domain could have favoured particular study types or technologies. The four domains were therefore treated as equally necessary for inclusion in a balanced biomaterials-oriented synthesis. Nevertheless, the QA framework should be interpreted as a structured appraisal rubric developed for the purposes of this review, rather than as a formally validated methodological instrument.

The four-domain structure was selected to ensure that each included study was assessed not only for general methodological clarity, but also for its relevance to biomaterial-based AM. This was necessary because the review includes heterogeneous evidence types, including experimental studies, computational analyses, process-oriented investigations, scaffold-fabrication studies, implant-focused studies and bioprinting-related contributions. The scoring system was therefore designed to capture whether each study provided: (i) a coherent research design; (ii) sufficient definition of the biomaterial system and AM route; (iii) transparent experimental, computational or analytical procedures; and (iv) clear biomedical or translational relevance. Equal weighting was retained to avoid favouring one material class, process route or study type over another.

A QA threshold of 60% was adopted as the minimum score for inclusion in the analytical synthesis. This threshold was selected because it required a study to satisfy more than half of the appraisal framework while still allowing inclusion of technically relevant contributions from emerging biomaterial systems and early-stage biofabrication routes, where reporting practices remain heterogeneous. Studies scoring between 50% and 60% were treated as borderline cases and were re-examined manually. These studies were retained only when they provided relevant technical, biological or translational information that was not adequately represented elsewhere in the corpus. Studies scoring below 50% were excluded because they lacked sufficient methodological transparency, biomaterial/process definition or biomedical relevance to support reliable synthesis.

To improve the transparency and reproducibility of the quality-assessment procedure, Table A2 summarises the score ranges used to classify the methodological and biomedical relevance of the studies considered for synthesis. The table clarifies how total QA percentages were interpreted, how inclusion decisions were made and how borderline studies were treated during manual reassessment.

**Table A2:** Summary of QA scoring categories applied to the studies retained for synthesis.

QA Score Range	Interpretation	Decision Rule	Role in Synthesis
≥80%	High methodological and biomedical relevance	Included	Used as core evidence for process–material–performance synthesis
60–79%	Acceptable methodological quality and relevance	Included	Used in the main synthesis when aligned with review scope
50–59%	Borderline methodological quality or incomplete reporting	Manually re-examined	Retained only when technically relevant and not duplicated elsewhere
<50%	Insufficient transparency, relevance or methodological detail	Excluded	Not used in the analytical synthesis

This classification was used as a structured appraisal framework rather than as a purely mechanical exclusion tool. Studies achieving scores of 60% or above were retained for synthesis, whereas borderline studies scoring between 50% and 60% were re-examined manually and included only when they provided relevant technical, biological or translational evidence not adequately represented elsewhere. The preliminary distribution generated during metadata-assisted filtering and keyword-based prioritisation is presented in Figure A3.

Figure A3 should therefore be interpreted as a preliminary screening and prioritisation output, not as evidence that full methodological quality assessment was performed automatically for all retrieved records.

The identification and selection of relevant publications followed a three-stage screening procedure consistent with the PRISMA 2020 framework, thereby ensuring a transparent and methodologically systematic progression from the initial retrieval of records to the final inclusion of eligible studies. In the first stage, all search results were consolidated into a single dataset, and duplicate records were removed through a combination of automated processing and manual verification using the customised MATLAB^®^ routine described previously.

As illustrated in Figure A4 the geographical distribution of authorship and institutional affiliation within the reviewed literature indicates a marked concentration of research activity in countries with established strengths in biomaterials science, biomedical engineering, advanced manufacturing and translational healthcare technologies. The United States of America emerges as the most prominent contributor, followed by China, United Kingdom and Italy, with India and Germany also occupying significant positions within the scholarly output on biofabrication, scaffold design, bioprinting and implant-oriented AM. This distribution reflects the scientific and clinical relevance of the field in sectors such as regenerative medicine, orthopaedics, dentistry, tissue engineering and patient-specific medical-device development, where demanding combinations of biological compatibility, structural integrity and manufacturing precision are required.

This geographical pattern is of particular significance, as it indicates that research on the AM of biomaterials is not confined to a narrow group of countries traditionally associated with technological innovation. Rather, the field exhibits an increasingly international research profile, reflecting the global importance of efforts to improve printability, biological functionality, structural fidelity, manufacturing reliability and the broader clinical translation of biomaterial-based constructs. Collectively, these trends suggest that biomaterial-centred AM has developed into a strategically important and internationally distributed area of research, supported by sustained contributions from both established and emerging centres of scientific and technological expertise.

### Appendix A.5. Generation and Interpretation of Descriptive and Conceptual Figures

The descriptive figures reporting publication trends, geographical distribution and preliminary quality-screening outputs were generated from the cleaned bibliographic dataset after duplicate removal, metadata harmonisation and eligibility-based filtering. Publication-year counts were extracted from the publication year recorded in the bibliographic metadata. Where multiple date fields were available, the final publication year was prioritised over early-access or online-first dates to avoid double counting. Each publication was counted once after DOI-based deduplication. When DOI information was absent or inconsistently indexed, duplicate detection was supported by normalised title, abstract and keyword similarity, followed by author verification.

Country-level distributions were derived from the institutional-affiliation metadata available in the bibliographic records. To avoid inflating counts for internationally co-authored papers, each record was assigned to a single country based on the first available institutional affiliation/country field in the exported metadata. Multi-country publications were therefore not counted multiple times in the geographical-distribution figure. This analysis was used only as contextual bibliographic information and did not influence study inclusion, quality assessment or interpretation of scientific findings.

The preliminary quality-screening distribution was generated during metadata-assisted filtering and keyword-based prioritisation. It should not be interpreted as a fully automated methodological quality assessment of all retrieved records. Final study-level QA decisions were performed manually by the authors during title, abstract and full-text assessment according to the predefined QA domains and inclusion criteria.

The descriptive graphical outputs were produced from the cleaned dataset using MATLAB^®^-assisted processing and spreadsheet-based verification, followed by visual formatting for publication. In contrast, the conceptual framework figures included in the main manuscript are author-developed interpretative schematics derived from the reviewed literature. They are not quantitative bibliometric outputs. Their purpose is to support conceptual integration of biomaterial selection, AM route, process–structure–property relationships, biological response, post-processing, quality control and translational readiness.

All figures were reviewed for readability during revision. Font sizes were increased where possible, dense panels were simplified, and the figures presenting complex conceptual frameworks were reformatted to improve contrast, spacing and interpretability.

### Appendix A.6. Descriptive Analysis and Taxonomic Synthesis

The descriptive synthesis of the final body of literature reveals a technically diverse and progressively expanding field of research, characterised by the increasing convergence of biomaterials science, AM, tissue engineering, materials processing and translational biomedical engineering. The selected studies were classified according to their technological orientation, material focus and degree of experimental, biological or application-driven depth, thereby enabling the identification of the principal trajectories along which contemporary research on the AM of biomaterials is developing. Overall, the reviewed literature may be understood as converging around two closely related research domains.

1. Biomaterial systems and process compatibility: This domain encompasses investigations concerned with the selection, formulation and functional behaviour of biomaterials intended for additive-manufacturing routes. Particular attention is given to the influence of material composition on printability, rheological behaviour, structural integrity, degradation characteristics and biological performance. Studies within this category examine the effects of variables such as viscosity, crosslinking behaviour, powder characteristics, thermal sensitivity, biocompatibility and feedstock composition on fabrication reliability and the quality of the resulting constructs. Research in this area is directed towards improving material–process compatibility, reproducibility and application-specific performance, whilst minimising defects, instability and limitations associated with processing-induced damage.
2. Fabrication performance, post-processing and biomedical applicability: The second domain concerns the manufacture, refinement and evaluation of biomaterial-based constructs intended for biomedical use. This body of work includes studies focused on scaffold fabrication, bioprinting, dimensional and structural fidelity, post-processing strategies, mechanical competence and biological functionality, together with investigations into their influence on the usability and translational relevance of the final constructs. These approaches are intended to overcome the limitations of as-fabricated parts and to support the reliable deployment of biomaterial-based additive-manufacturing routes in clinically relevant applications.

This classification highlights the inherently multidisciplinary character of current research in this field, in which biomaterials engineering, manufacturing science, cell biology, materials characterisation and biomedical design intersect in order to address the complex material, biological and procedural challenges associated with the fabrication of advanced biomedical constructs. Across the reviewed literature, four principal thematic directions may be distinguished.

1. Material limitations and construct quality: A substantial proportion of the literature is devoted to the identification, characterisation and mitigation of limitations affecting the AM of biomaterials, including insufficient mechanical strength, restricted printability, poor resolution, residual porosity, anisotropy, limited bioactivity and reduced cell viability. These studies seek to clarify the relationships between material properties, process conditions and final construct performance, with the objective of improving structural reliability and biomedical functionality.
2. Optimisation of process parameters and repeatability: Another major line of investigation concerns the optimisation of operational variables in order to improve fabrication quality, reproducibility and workflow stability. Particular emphasis is placed on the selection of process conditions capable of controlling layer formation, dimensional fidelity, curing behaviour, thermal history, deposition stability and construct architecture. In this context, increasing attention is also devoted to the repeatability and reproducibility of biomaterial-processing routes across different platforms, material systems and biomedical applications.
3. Biological evaluation, mechanical performance and translational applicability: Recent studies increasingly examine the capacity of different validation procedures to assess biological response, structural performance and practical usability. This includes analyses of cell viability, scaffold integrity, degradation behaviour, mechanical competence and tissue-specific suitability, as well as investigations into the technical constraints and translational relevance of different fabrication routes for regenerative medicine, implantology and related biomedical domains.
4. Advanced biofabrication and process innovation: A further area of development involves the use of improved hydrogel formulations, bioink engineering, multi-material strategies, controlled-porosity design and process-optimisation approaches to enhance fabrication efficiency and construct performance. These studies address material tailoring, architectural control, post-processing refinement and the integration of biologically functional components, thereby contributing to more robust, versatile and clinically relevant biofabrication systems.

Across the reviewed studies, several recurrent technical gains are reported, including improved printability and processing stability; enhanced structural fidelity and scaffold quality; improved mechanical and biological performance; greater confidence in application-specific suitability; a clearer understanding of the relationship between material properties and process behaviour; and broader biomedical applicability through integrated material and fabrication strategies.

Notwithstanding these advances, the literature also identifies several persistent challenges that continue to limit broader translational and industrial implementation. These include the difficulty of achieving simultaneously high structural fidelity and biological performance, the complexity of correlating material properties with final construct quality, the limited standardisation of biological validation procedures, the cost and technical constraints associated with advanced fabrication platforms, and the challenge of integrating material development, processing control, post-processing and clinical requirements into coherent and practically viable manufacturing workflows. Additional constraints arise from the variability of reported material formulations, fabrication settings, validation criteria and performance indicators across different technologies and application domains.

The PRISMA2020 flow diagram reports the complete transition from database-level retrieval to automated metadata-assisted filtering, manual screening, full-text eligibility assessment and final inclusion. The large initial retrieval value represents the database output generated by the Boolean search strategy, whereas final inclusion decisions were made manually after staged filtering and full-text assessment.

The review has been retrospectively registered on the Open Science Framework (OSF) (DOI: 10.17605/osf.io/u4cgs; url: https://osf.io/8qhj7; accessed on 18 July 2026).

### Appendix A.7. PRISMA 2020 Flow Diagram for New Systematic Reviews, Which Included Searches of Databases and Registers

This section presents the PRISMA 2020 flow diagram used to document the identification, screening, eligibility assessment and final inclusion of studies considered in the present systematic review. The diagram provides a transparent representation of the literature-selection pathway and supports methodological traceability, consistency and reproducibility in accordance with the PRISMA 2020 framework.

The systematic search strategy was designed to identify peer-reviewed literature published between 2010 and 2025 concerning the AM of biomaterials, including biomaterial classes, fabrication routes, scaffold development, bioprinting strategies, post-processing requirements, biological performance and biomedical applicability. To ensure thematic precision, the search incorporated three predefined keyword groups: biomaterial systems, additive-manufacturing technologies and biomedical or translational performance criteria. These terms were combined through Boolean logic and applied to the title, abstract and keyword fields of the selected databases. The complete study-selection procedure, including database-level retrieval, automated metadata-assisted filtering, manual screening, full-text eligibility assessment and final inclusion, is illustrated in Figure A5.

The initial retrieval value reported in Figure A5 therefore represents the broad database-level output generated by the Boolean search strategy, whereas the subsequent manual screening and full-text eligibility stages were conducted only after deduplication, metadata cleaning, language and document-type filtering and preliminary keyword-field eligibility checks.
