# Supplementary material for: Additive Manufacturing of Biomaterials: Integrated Translational Ecosystems, Process–Structure–Property Interactions and Emerging Paradigms for Next-Generation Biomedical Engineering
Source: J Funct Biomater. 2026 Jul 30;17(8):365. doi: 10.3390/jfb17080365 (PMC13514614; doi:10.3390/jfb17080365)
Supplement: Supplementary file 1 [file jfb-17-00365-s001.zip › jfb-4374891-supplementary.pdf]

## Table S1. Preferred Reporting Items for Systematic reviews and Meta-Analyses extension for Scoping Reviews (PRISMA-ScR) Checklist

| SECTION | ITEM | PRISMA-ScR CHECKLIST ITEM | REPORTED ON PAGE # |
|---|---|---|---|
| **TITLE** | | | |
| Title | 1 | Additive Manufacturing of Biomaterials: Integrated Translational Ecosystems, Process–Structure–Property Interactions and Emerging Paradigms for Next-Generation Biomedical Engineering. | p. 1 |
| **ABSTRACT** | | | |
| Structured summary | 2 | The abstract summarises the background, rationale, integrated ecosystem perspective, biomaterial classes and AM routes assessed, principal technical and translational constraints, and future priorities for clinically relevant AM-based biomaterials. | p. 2 |
| **INTRODUCTION** | | | |
| Rationale | 3 | The introduction establishes the biomedical relevance of biomaterials and AM, explains the limitations of isolated material- or process-centred reviews, and justifies an integrated biomaterial–process–structure–property–translation perspective. | pp. 2–8 |
| Objectives | 4 | The review aims to examine AM of biomaterials across material classes, acellular and cellular routes, post-processing, biological performance, process–structure–property relationships, quality assurance and translational readiness. | pp. 7–8; Appendix A |
| **METHODS** | | | |
| Protocol and registration | 5 | No formal review protocol was registered. The study followed a PRISMA-informed methodological framework to ensure transparency, traceability and reproducibility in study identification, screening, quality assessment and synthesis. | pp. 8–10; Appendix A.1–A.7 |
| Eligibility criteria | 6 | Eligibility criteria included English-language peer-reviewed publications from 2010 to 2025 addressing biomaterials in combination with AM, biofabrication or bioprinting processes and biomedical relevance. Exclusions covered non-peer-reviewed sources, grey literature, conventional manufacturing without AM relevance, insufficient methodological detail and studies outside the defined scope. | pp. 8–10; Appendix A.3 |
| Information sources* | 7 | Literature searches were conducted principally using CrossRef, with supplementary verification through Dimensions.ai to broaden retrieval and improve metadata completeness. A bespoke MATLAB® routine supported metadata standardisation and deduplication. | pp. 8–9; Appendix A.2 |
| Search | 8 | The search strategy combined three conceptual blocks: biomaterial systems, additive-manufacturing technologies, and biomedical or translational performance criteria. Boolean strings were applied to title, abstract and keyword fields. | pp. 8–9; Appendix A.2 |
| Selection of sources of evidence† | 9 | Records were consolidated using DOI-based and title/abstract/keyword similarity checks, then screened through title, abstract and keyword assessment, full-text eligibility review and quality assessment against predefined inclusion and exclusion criteria. | pp. 9–10; Appendix A.3 and A.7 |
| Data charting process‡ | 10 | Data charting involved structured extraction of information on biomaterial class, AM route, feedstock or formulation, processing conditions, construct architecture, post-processing, biological or mechanical performance and translational relevance. | Section 2; Appendix A.6 |
| Data items | 11 | Extracted data included biomaterial family, manufacturing route, process parameters, scaffold or construct characteristics, post-processing requirements, printability, mechanical behaviour, cell viability, biological response, application domain and reported limitations. | Sections 2–3; Appendix A.6 |
| Critical appraisal of individual sources of evidence§ | 12 | A structured QA framework evaluated research design, biomaterial and manufacturing definition, methodological rigour and biomedical or translational relevance. Studies scoring ≥60% were retained; borderline cases were manually reassessed. | pp. 9–10; Appendix A.4 |
| Synthesis of results | 13 | The selected evidence was synthesised by material class, acellular AM route, cellular bioprinting strategy, post-processing requirement, biological performance, process–structure–property relationship, and translational or regulatory constraint. | Sections 3–4; Appendix A.6 |
| **RESULTS** | | | |
| Selection of sources of evidence | 14 | The corrected workflow retained “Biomaterials” as the anchoring term. The search identified 1,259,752 records, reduced to 879,309 screened records after metadata filtering, with 126 studies retained for final synthesis. | Appendix A; Figures 16 and 20 |
| Characteristics of sources of evidence | 15 | The included sources cover metallic, ceramic, polymeric, hydrogel/bioink and composite biomaterials processed through BJ, DED, material extrusion and jetting, PBF, vat photopolymerisation and bioprinting routes. | Sections 3.1–3.2; Tables 2–4 |
| Critical appraisal within sources of evidence | 16 | QA results were used to preserve methodological reliability while retaining technically relevant contributions on emerging AM biomaterials, biofabrication strategies and translational biomedical applications. | Appendix A.4; Figure 18 |
| Results of individual sources of evidence | 17 | Results from individual studies are discussed across the technology-specific subsections, including binder jetting, directed energy deposition, material extrusion and jetting, powder bed fusion, vat photopolymerisation and bioprinting. | Sections 3.1.1–3.2 |
| Synthesis of results | 18 | Integrated synthesis identifies key material–process trade-offs, biological implications, process–structure–property interactions, emerging paradigms, regulatory bottlenecks and future requirements for clinically robust AM biomaterials. | Sections 4.1–4.3; Figures 13–15 |
| **DISCUSSION** | | | |

| SECTION | ITEM | PRISMA-ScR CHECKLIST ITEM | REPORTED ON PAGE # |
|---|---|---|---|
| Summary of evidence | 19 | The discussion and conclusions summarise the evidence that clinical impact depends on integrated optimisation of biomaterial selection, processing windows, structural fidelity, post-processing, biological validation and translational readiness. | Sections 4–5 |
| Limitations | 20 | Limitations include restricted material–process compatibility, limited prediction of process–structure–property relationships, post-processing effects on biological performance, insufficient standardised metrics, incomplete validation of cell-laden constructs and weak transfer from laboratory protocols to clinical workflows. | pp. 7–8; Sections 4.3 and 6 |
| Conclusions | 21 | The review establishes an ecosystem-level framework linking biomaterial chemistry, AM route, architecture, post-processing, biological response, quality assurance and translational readiness for next-generation biomedical constructs. | Section 5 |
| **FUNDING** | | | |
| Funding | 22 | No funding was received to carry out this study. | Funding section |

JBI = Joanna Briggs Institute; PRISMA-ScR = Preferred Reporting Items for Systematic reviews and Meta-Analyses extension for Scoping Reviews.

* Where sources of evidence are compiled from, such as bibliographic databases, social media platforms, and Web sites.

† A more inclusive/heterogeneous term used to account for different types of evidence or data sources that may be eligible in a scoping review.

‡ The frameworks by Arksey and O'Malley and Levac and colleagues, and the JBI guidance, refer to the process of data extraction in a scoping review as data charting.

§ The process of systematically examining research evidence to assess its validity.
